# Adverse perinatal outcomes attributable to HIV in sub-Saharan Africa from 1990 to 2020: Systematic review and meta-analyses

**DOI:** 10.1038/s43856-023-00331-8

**Published:** 2023-07-22

**Authors:** Claudia Murray, Clara Portwood, Harriet Sexton, Mary Kumarendran, Zoe Brandon, Shona Kirtley, Joris Hemelaar

**Affiliations:** 1 National Perinatal Epidemiology Unit, Oxford Population Health, Nuffield Department of Population Health, University of Oxford, Oxford, UK; 2 Centre for Statistics in Medicine, Nuffield Department of Orthopaedics, Rheumatology and Musculoskeletal Sciences, University of Oxford, Oxford, UK

**Keywords:** Epidemiology, Paediatric research

## Abstract

**Background:**

Maternal HIV infection and antiretroviral drugs (ARVs) are associated with increased risks of adverse perinatal outcomes. The vast majority of pregnant women living with HIV (WLHIV) reside in sub-Saharan Africa. We aimed to determine the burden of adverse perinatal outcomes attributable to HIV and ARVs in sub-Saharan Africa between 1990 and 2020.

**Methods:**

We conduct a systematic review of studies on the association of pregnant WLHIV with adverse perinatal outcomes in sub-Saharan Africa. We perform random-effects meta-analyses to determine the risk difference (attributable risk, AR) of perinatal outcomes among WLHIV receiving no ARVs, monotherapy, or combination antiretroviral therapy (cART) initiated antenatally or preconception, compared to HIV-negative women. We estimate numbers of perinatal outcomes attributable to HIV and ARVs by combining the AR values with numbers of WLHIV receiving different ARV regimens in each country in sub-Saharan Africa annually between 1990 and 2020.

**Results:**

We find that WLHIV receiving no ARVs or cART initiated antenatally or preconception, but not monotherapy, have an increased risk of preterm birth (PTB), low birthweight (LBW) and small for gestational age (SGA), compared to HIV-negative women. Between 1990 and 2020, 1,921,563 PTBs, 2,119,320 LBWs, and 2,049,434 SGAs are estimated to be attributable to HIV and ARVs in sub-Saharan Africa, mainly among WLHIV receiving no ARVs, while monotherapy and preconception and antenatal cART averted many adverse outcomes. In 2020, 64,585 PTBs, 58,608 LBWs, and 61,112 SGAs were estimated to be attributable to HIV and ARVs, the majority among WLHIV receiving preconception cART.

**Conclusions:**

As the proportion of WLHIV receiving preconception cART increases, the burden of adverse perinatal outcomes among WLHIV in sub-Saharan Africa is likely to remain high.

**Systematic review registration number:**

CRD42021248987

## Plain Language Summary

Pregnant women living with HIV (WLHIV) are at higher risk of adverse birth outcomes, such as babies born too soon (premature birth), babies born too small (low birthweight) or small-for-gestational-age (smaller than expected based on the weeks of pregnancy). It is unknown how many cases of these outcomes are attributable to HIV in sub-Saharan Africa, where most pregnant WLHIV reside. We conduct a search for published studies to determine the risk of adverse birth outcomes among WLHIV. We find that around 2 million premature births, low birthweight babies, and small-for-gestational-age babies are attributable to HIV in sub-Saharan Africa between 1990 and 2020. We conclude that adverse birth outcomes among WLHIV in sub-Saharan Africa are likely to remain high for the foreseeable future. Our findings could guide strategies to improve the health of WLHIV and their children in this region.

## Abstract (web summary)

Murray et al. conduct a systematic review, meta-analysis and modelling study to estimate the numbers of adverse perinatal outcomes attributable to HIV and antiretroviral therapy (ART) in sub-Saharan Africa from 1990 to 2020. They find that large numbers of adverse outcomes are attributable to HIV/ART, increasingly amongst women receiving preconception ART.

## Introduction

Neonatal morbidity and mortality are the leading cause of disability adjusted life years worldwide^1^. United Nations’s Sustainable Development Goal 3 (SDG3) target 3.2 aims to reduce neonatal and under-5 mortality to 12 and 25 per 1000 live births, respectively, by 2030^2^. sub-Saharan Africa has the highest neonatal and child mortality rates globally and most countries in this region are predicted not to reach these SDG3 targets^3^. Adverse perinatal outcomes are major contributors to neonatal and child morbidity and mortality. Preterm birth (PTB) is the most important cause of neonatal and child mortality globally^4^, with an estimated 14.8 million cases annually^5^. Small for gestational age (SGA) infants, with annual numbers of 23.3 million in low- and middle-income countries (LMICs), contribute towards 21.9% of neonatal deaths^6^. Both PTB and SGA contribute towards low birthweight (LBW), an outcome often used when gestational age is unknown, with 18 million LBW cases estimated annually in LMICs^7^.

37.7 million people worldwide were living with HIV in 2020, including 19.3 million women of childbearing age^8^. Each year, 1.3 million women living with HIV (WLHIV) are pregnant, with the vast majority residing in sub-Saharan Africa^8^. Antiretroviral therapy (ART) improves the health of WLHIV and prevents vertical transmission of HIV^9^. Antiretroviral drugs (ARVs) recommended for pregnant WLHIV have changed over time, as treatments and evidence evolved. The first treatment available for pregnant WLHIV was antenatal zidovudine (ZDV) monotherapy, which was demonstrated to reduce the risk of vertical HIV transmission^10^. In 2010, World Health Organization (WHO) guidelines recommended ZDV monotherapy in pregnant WLHIV to prevent vertical HIV transmission and combination antiretroviral therapy (cART) for pregnant WLHIV requiring treatment for their own health (Option A) or cART for prevention of vertical HIV transmission as well as maternal health (Option B)^11^. In 2013, the WHO recommended that all pregnant WLHIV should receive cART^12^. These guidelines led to a decrease in the global proportion of pregnant WLHIV receiving ZDV monotherapy from 31% to 0% between 2011 and 2020, while the proportion of pregnant WLHIV receiving cART increased from 27 to 83% during the same period^8^. Since 2015, WHO recommend that all people living with HIV should initiate lifelong cART as soon as possible after diagnosis, including pregnant WLHIV^13^. This resulted in a dramatic increase in the proportion of pregnant WLHIV who received cART at the time of conception, from 7% in 2010 to 89% in 2020, in the 21 focus countries^14^. These trends were accompanied by a 47% reduction in vertical HIV transmission globally between 2010 and 2020^15^.

Pregnancies in WLHIV without ARVs are associated with an increased risk of PTB, LBW, SGA and stillbirth compared to HIV-negative women^16^. While the benefits of ARVs for maternal health and prevention of vertical transmission of HIV are clear, several studies suggest ARV exposure during pregnancy is associated with adverse perinatal outcomes, but evidence is conflicting regarding different regimens^17,18^. A recent network meta-analysis of seven randomised controlled trials (RCTs), which compared ARV regimens initiated among WLHIV during pregnancy, showed that a number of cART regimens were associated with an increased risk of PTB, LBW, and very LBW, compared to ZDV monotherapy^19^. Some cohort studies report that cART exposure is associated with increased risk of PTB and LBW in pregnant WLHIV, while ZDV monotherapy is not^20,21^. However, others report no significant association^22^. Additionally, timing of cART initiation may play a role as a meta-analysis reported that preconception cART initiation was associated with an increased risk of PTB, compared to antenatal cART initiation^23^.

To reach SDG3 targets for reductions of neonatal and under-5 mortality it is crucial to estimate the magnitude and trends of factors that contribute to these outcomes^1,3^. As sub-Saharan Africa has the highest rates of neonatal and child morbidity and mortality^3^ and the vast majority of pregnant WLHIV reside in this region^8^, we aimed to estimate the burden of adverse perinatal outcomes of pregnant WLHIV in sub-Saharan Africa between 1990 and 2020. To this end, we determined the attributable risk of specific perinatal outcomes for WLHIV receiving different ARV regimens, compared to HIV negative women, and combined this with data on the annual numbers of pregnant WLHIV receiving the different ARV regimens in every country in sub-Saharan Africa between 1990 and 2020. We find that around 2 million preterm births, low birthweight babies, and small-for-gestational-age babies are attributable to HIV and ARVs in sub-Saharan Africa between 1990 and 2020.

## Methods

### Search strategy

The systematic review and meta-analyses were conducted according to a protocol based on the Cochrane guidelines^24^ and registered online (PROSPERO, number CRD42021248987). The systematic review is reported according to the Preferred Reporting Items for Systematic Reviews and Meta-Analyses (PRISMA) guidelines^25^. We searched PubMed, CINAHL (Ebscohost), Global Health (Ovid), and EMBASE (Ovid) for studies conducted in sub-Saharan Africa and published between January 1, 1980, and April 20, 2020, using a comprehensive search strategy adapted for each database, developed by a specialist librarian (SK). Both free text and controlled vocabulary search terms for “HIV”, “antiretroviral therapy”, “pregnancy outcome”, and “specific perinatal outcomes” were used. No methodological or language filters were applied. Full-text articles and abstracts were considered. For full search terms see Supplementary Note 1. Retrieved articles were imported into EndNote reference manager (EndNote X9; Clarivate Analytics, Pennsylvania, USA) and deduplicated.

### Eligibility criteria

Studies conducted in sub-Saharan Africa that contained information on the association of pregnant WLHIV with adverse perinatal outcomes were eligible. Inclusion criteria were study design (cohort studies), location (countries in sub-Saharan Africa), population (pregnant women), exposure (WLHIV with or without ARV exposure), and comparator (HIV-negative women). ARV exposures included antenatal monotherapy and cART initiated either antenatally or preconception. Monotherapy exposure was defined as receiving one antiretroviral drug (ZDV) during pregnancy. cART exposure was defined as receiving any combination of ≥ 3 antiretroviral drugs. Timing of cART initiation, i.e. antenatal or preconception, had to be specified and mixed initiation groups were excluded. Single dose nevirapine (NVP) at birth or antenatal ARV duration < 30 days were not considered ARV exposure. Studies were not included if less than 95% of women in an exposure or comparator group conformed to the exposure or comparator definition (e.g. < 95% WLHIV received monotherapy) or if additional treatment was received by one exposure or comparator group only (e.g., anti-tuberculosis treatment). Perinatal outcomes of interest were defined as follows: PTB (birth < 37^+0^ weeks gestation)^26^, very PTB (VPTB, birth < 32^+0^ weeks gestation)^26^, LBW ( < 2500 g)^7^, very LBW (VLBW, < 1500 g)^7^, SGA (birthweight for gestational age < 10th centile)^27^, very SGA (VSGA, birthweight for gestational age < 3rd centile)^27^ and neonatal death (NND, death of an infant in the first 28 days of life)^28^. Data for spontaneous PTB, term and preterm LBW, and stillbirth were also sought, but insufficient data was found to enable modelling analysis. We would like to acknowledge that not every pregnant person is a woman, despite the gendered language used throughout this article. We have used terms such as WLHIV for simplicity and consistency with their use in the studies and databases used for this analysis.

### Study selection and data extraction

Titles and abstracts of citations were reviewed, and full text manuscripts of selected citations assessed against the eligibility criteria by at least two independent investigators (CP, HS, MK, and ZB). If a cohort was reported on more than once, the study containing the most recent and complete data was included. If multiple publications reported different perinatal outcomes for the same cohort, each study was included. Studies were excluded if outcome data were not stratified according to exposure and comparator categories and if exposures and outcomes were not defined or defined differently from those detailed here. References of studies meeting the inclusion criteria were assessed for additional studies. Ambiguities regarding inclusion of studies were resolved through discussion with the senior investigator (JH). Data on study characteristics, HIV/ARV exposures and perinatal outcomes were extracted from eligible studies by at least two investigators (CP, HS, MK, and ZB) and reviewed by the senior investigator (JH).

### Quality assessment

The quality of individual studies was assessed using an adapted Newcastle-Ottawa Scale by at least two investigators (CP, HS, MK, and ZB) and reviewed by the senior investigator (JH)^29^. Nine criteria were assessed in three groups: selection of study participants, comparability of comparator groups, and assessment of outcomes of interest. Studies were defined as ‘good’, ‘average’, or ‘poor’ quality according to predefined criteria (Supplementary Notes 2 and 3).

### Statistical analysis

Perinatal outcomes of WLHIV receiving no ARVs, monotherapy, or cART initiated antenatally or preconception were compared to HIV-negative women. Dichotomous outcome data according to HIV/ARV exposure from individual studies were used to calculate attributable risk (AR) values (i.e. the absolute risk difference between WLHIV in each ARV category and HIV-negative women) and 95% confidence intervals (CIs). Pairwise meta-analyses were carried out if two or more studies reported data for the same perinatal outcome for HIV-negative women and WLHIV receiving no ARVs, monotherapy, antenatal cART or preconception cART. For all meta-analyses, a random-effects model was used to calculate a weighted summary effect estimate (AR) and 95% CI, which were represented in forest plots (Supplementary Figs. 1–24). The *I*^2^ statistic was used to quantify heterogeneity due to clinical and methodological variability between studies. Peters’ test was used to assess publication bias in meta-analyses containing ≥10 studies. We conducted subgroup analyses based on country income status and sensitivity analyses according to study quality (Supplementary Figs. 25–28). All statistical analyses were performed in Stata version 15 (College Station, Texas, USA).

### Modelling

For every country in sub-Saharan Africa estimates of annual numbers of pregnant WLHIV and ARV coverage between 1990 and 2020 were obtained from UNAIDS^8^. We consolidated different UNAIDS ARV categories into the four ARV categories (no ARVs, monotherapy, antenatal cART, and preconception cART) used in our meta-analysis. WLHIV receiving no ARVs included the UNAIDS categories ‘no ART’, ‘single dose nevirapine’, and ‘Option B+ started during current pregnancy < 4 weeks before delivery’. WLHIV receiving monotherapy included the UNAIDS categories ‘Option A (antenatal ZDV)’ and ‘dual ARV (antenatal ZDV and intrapartum NVP)’. WLHIV receiving antenatal cART included the UNAIDS categories ‘Option B triple prophylaxis from 14 weeks’ and ‘Option B+ started > 4 weeks before delivery’. WLHIV receiving preconception cART included the UNAIDS category ‘Option B + ART started before pregnancy’. This allowed us to calculate numbers of pregnant WLHIV in each ARV category in each year for every country in sub-Saharan Africa between 1990 and 2020. Estimates for the numbers of each perinatal outcome attributable to HIV and ARVs were calculated by multiplying the AR values for WLHIV in each ARV category by the estimates of the numbers of WLHIV in each ARV category. Only statistically significant AR values were used; AR values were set at 0 if meta-analysis results were not statistically significant. The number of adverse perinatal outcomes averted and contributed by ARV (compared to no ARVs) were estimated using the difference between AR values of WLHIV receiving no ARVs and the AR values of WLHIV receiving monotherapy, or cART initiated antenatally or preconception (each compared to HIV-negative women). Estimated numbers of adverse perinatal outcomes and confidence intervals are reported in Supplementary Data 1. To aid readability, only point estimates are used throughout the manuscript.

### Reporting summary

Further information on research design is available in the Nature Portfolio Reporting Summary linked to this article.

## Results

### Systematic review

**Fig. 1:**
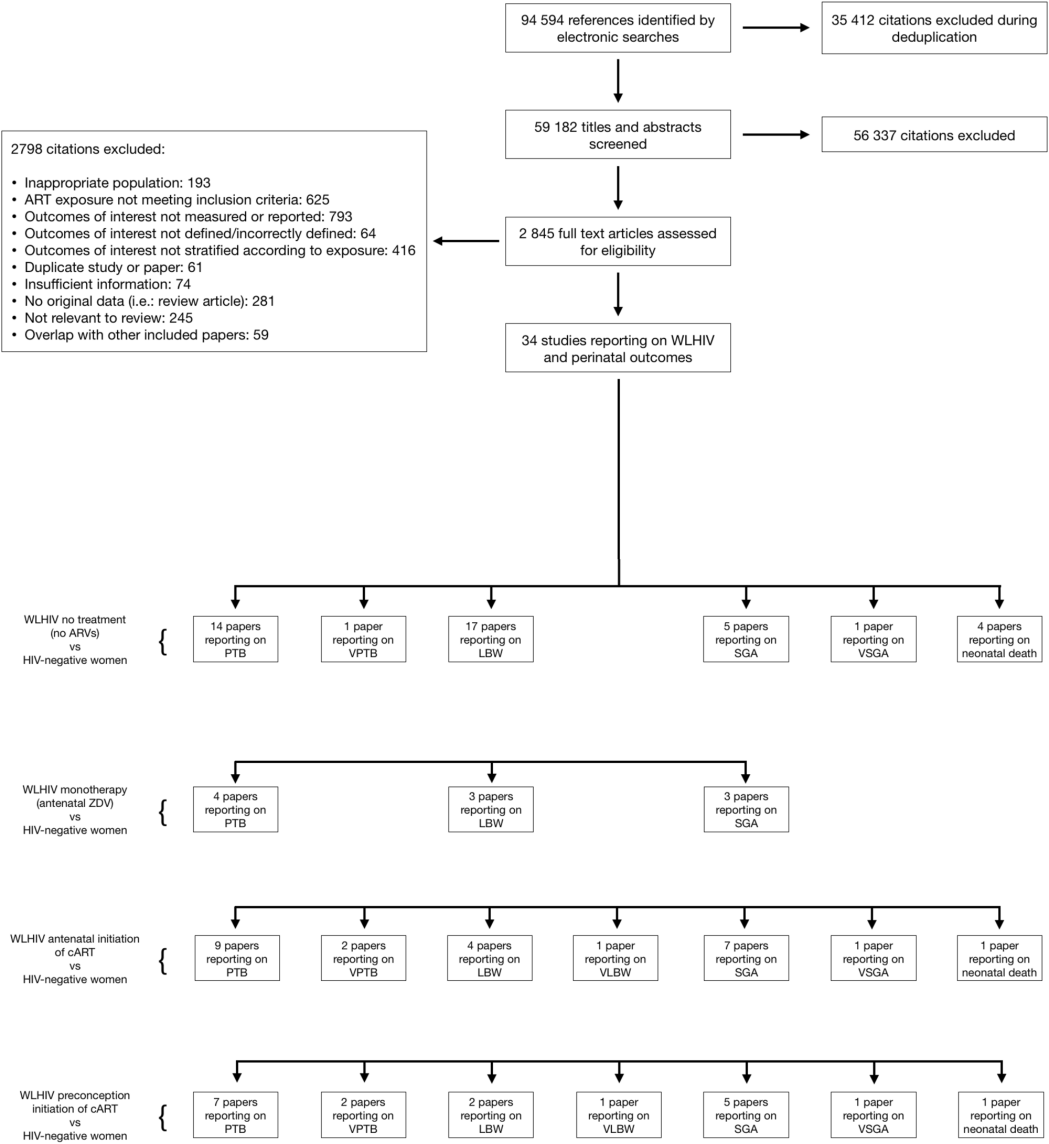
Study selection. Flow chart demonstrating the process of the literature review, showing the number of citations remaining and removed at each stage. Citations removed at the eligibility assessment stage are sorted by reason for elimination. The number of included studies reporting on each perinatal outcome for each exposure comparison are given. ARVs Antiretroviral drugs, cART Combination antiretroviral therapy, HIV Human immunodeficiency virus, LBW Low birthweight, NND Neonatal death, PTB Preterm birth, SGA Small for gestational age, VLBW Very low birthweight, VPTB Very preterm birth, VSGA Very small for gestational age, WLHIV Women living with HIV. See Methods for definitions of perinatal outcomes.

The literature search yielded 94,594 citations, of which 34 reported relevant data (Fig. 1). 21 studies reported on WLHIV receiving no ARVs, 5 on WLHIV receiving monotherapy, 11 on WLHIV receiving antenatal cART and 8 on WLHIV receiving preconception cART, each compared to HIV-negative women (Supplementary Data 2). Studies reporting on WLHIV receiving no ARVs and cART initiated antenatally or preconception reported on PTB, VPTB, LBW, VLBW, SGA, VSGA and NND, whereas studies reporting on WLHIV receiving monotherapy reported on PTB, LBW and SGA.

Study characteristics are summarised in Supplementary Data 3^30–63^. 19 prospective (56%) and 15 retrospective (44%) cohort studies analysed data from 399,558 women in 14 countries in sub-Saharan Africa. Quality assessments classified 19 (56%) studies as average quality and 15 (44%) studies as poor quality (Supplementary Data 3, Supplementary Data 4).

### Meta-analyses

**Fig. 2:**
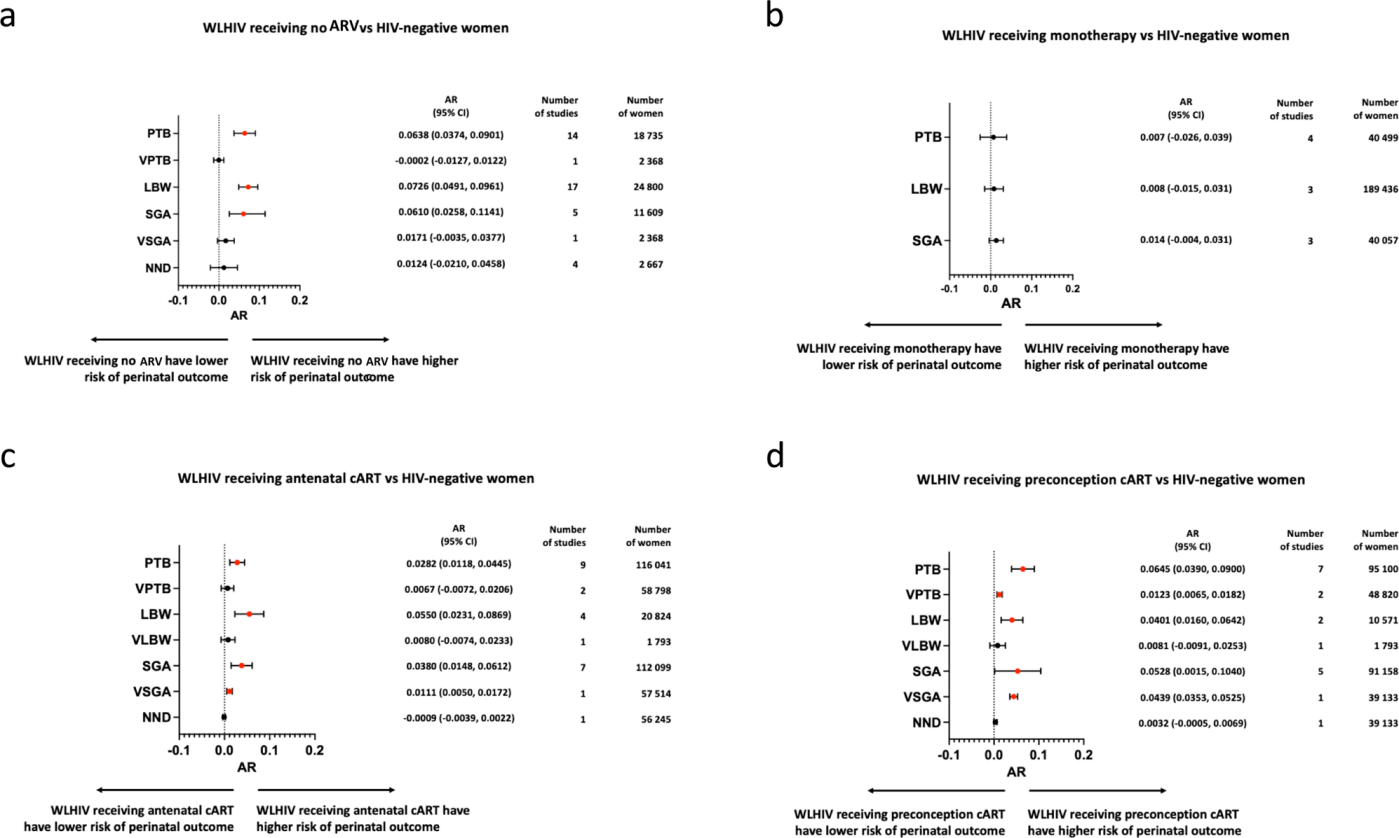
Perinatal outcomes of women living with HIV receiving different ARV regimens, compared to HIV-negative women. Results of random-effects meta-analyses to estimate attributable risk of perinatal outcomes associated with women living with HIV (WLHIV) receiving no ARVs (**a**), monotherapy (**b**) or cART initiated antenatally (**c**) and preconception (**d**), compared to HIV-negative women. Attributable risks, 95% confidence intervals and numbers of studies and women included in the analysis of each perinatal outcome for each comparison are displayed. Forest plots of meta-analyses can be found in Supplementary Figs. 1–24. Statistically significant results are presented with red dots and non-significant results with black dots. AR Attributable risk, ARVs Antiretroviral drugs, cART Combination antiretroviral therapy, CI Confidence interval, LBW Low birth weight, NND Neonatal death, PTB Preterm birth, SGA Small for gestational age, VPTB Very PTB, VLBW Very LBW, VSGA Very SGA, WLHIV Women living with HIV.

WLHIV receiving no ARVs had a significantly increased attributable risk of PTB (AR: 0.064 (i.e. 6.4% absolute risk increase); 95% CI: 0.037–0.090), LBW (0.073; 0.049–0.096) and SGA (0.061; 0.026–0.114) compared to HIV-negative women. WLHIV receiving no ARVs had an attributable risk of VPTB, VSGA and NND not significantly different from HIV-negative women (Fig. 2A).

WLHIV receiving monotherapy had an attributable risk of PTB, LBW or SGA not significantly different from HIV-negative women. No data was reported for VPTB, VLBW, VSGA or NND (Fig. 2B).

WLHIV receiving antenatal cART had a significantly increased attributable risk of PTB (0.028; 0.012–0.045), LBW (0.055; 0.023–0.087), SGA (0.038; 0.015–0.061) and VSGA (0.011; 0.005–0.017) when compared to HIV negative women. WLHIV receiving antenatal cART had an attributable risk of VPTB, VLBW and NND not significantly different from HIV-negative women (Fig. 2C).

WLHIV receiving preconception cART had a significantly increased attributable risk of PTB (0.065; 0.039–0.090), VPTB (0.012; 0.007–0.018), LBW (0.040; 0.016–0.064), SGA (0.053; 0.02–0.104) and VSGA (0.044; 0.035–0.053) when compared to HIV-negative women. WLHIV receiving preconception cART had an attributable risk of VLBW and NND not significantly different from HIV-negative women (Fig. 2D).

Peters’ test confirmed the absence of publication bias for meta-analyses containing ≥ 10 studies (Supplementary Figs. 1–24). Sensitivity and subgroup analyses showed that meta-analysis results of average quality studies were consistent with the overall results (Supplementary Figs. 25–28).

### ARV regimens received by pregnant WLHIV

**Fig. 3:**
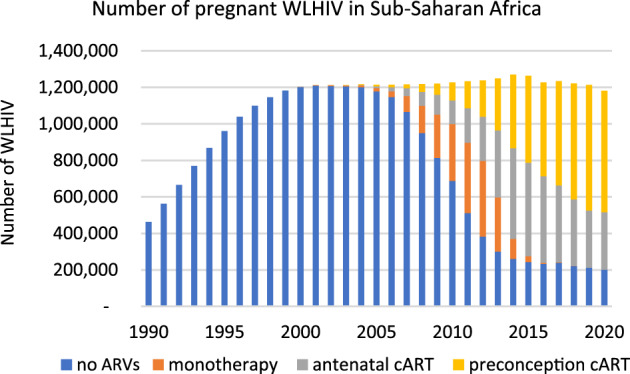
Pregnant women living with HIV receiving different ARV regimens in sub-Saharan Africa in 1990–2020. Number of pregnant women living with HIV receiving different ART regimens annually in sub-Saharan Africa between 1990 and 2020 (for data see Supplementary Data 1)^8^. ARVs Antiretroviral drugs, cART Combination antiretroviral therapy, WLHIV Women living with HIV.

Between 1990 and 2014, annual numbers of pregnant WLHIV in sub-Saharan Africa rose, reaching a peak of 1,269,829 in 2014, with numbers falling to 1,181,329 in 2020 (Fig. 3, Supplementary Data 1)^8^. Numbers of WLHIV receiving no ARVs increased from 1990, reaching a maximum of 1,209,649 in 2001, after which their number fell to 200,868 in 2020. Numbers of WLHIV receiving monotherapy increased from 2000, reaching 414,863 in 2012, before falling to 241 in 2020. From 2000, numbers of WLHIV receiving cART increased to 980,221 in 2020 (83% of WLHIV), with the majority receiving preconception cART in 2020 (664,898; 56% of WLHIV)(Fig. 3, Supplementary Data 1)^8^.

### Burden of adverse perinatal outcomes

#### Preterm birth

**Fig. 4:**
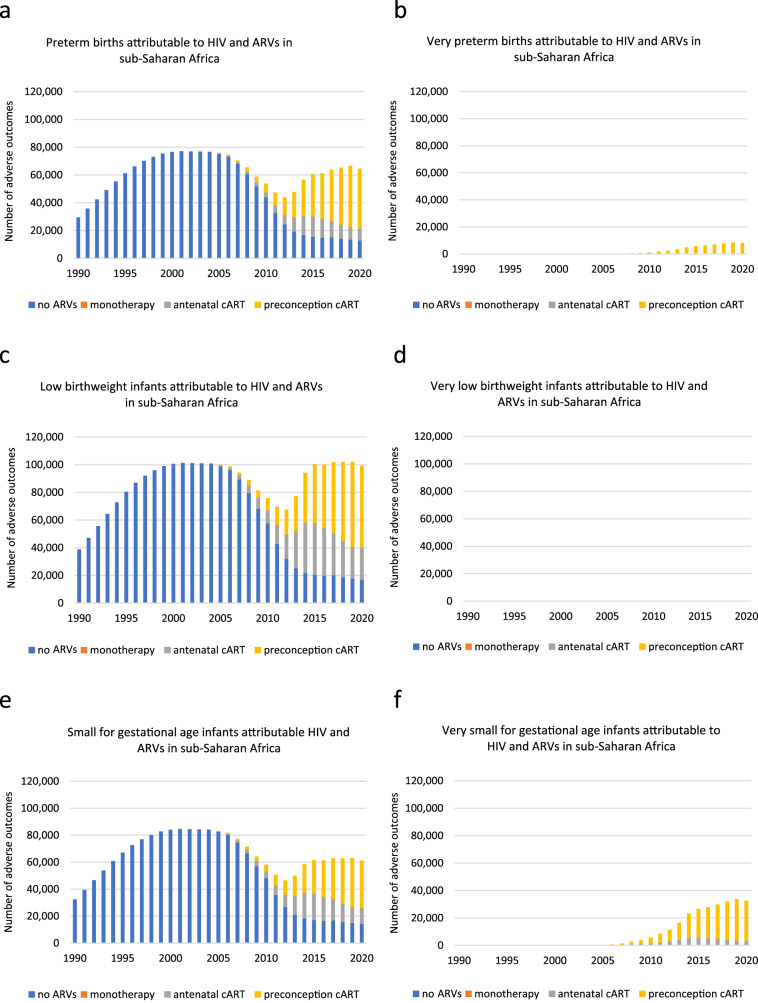
Adverse perinatal outcomes attributable to HIV and ARVs in sub-Saharan Africa in 1990–2020. Numbers of adverse perinatal outcomes attributable to HIV and ARVs annually between 1990 and 2020 (Supplementary Data 1). Pregnant women living with HIV received no ARVs, monotherapy, or cART initiated antenatally or preconception. Perinatal outcomes analysed: preterm birth (**a**), very preterm birth (**b**), low birthweight (**c**), very low birthweight (**d**), small for gestational age (**e**), very small for gestational age (**f**). Note that not all treatment groups of WLHIV had data for all perinatal outcomes and that not all comparisons to HIV-negative women were statistically significant (Fig. 2). Hence, not all categories in the legends (no ARVs, monotherapy, and antenatal and preconception cART) are represented in the graph for each perinatal outcome. Notably, data for very low birthweight and neonatal death was either not available or not statistically significant (Fig. 2a–d). ARVs Antiretroviral drugs, cART Combination antiretroviral therapy, HIV Human immunodeficiency virus.

**Fig. 5:**
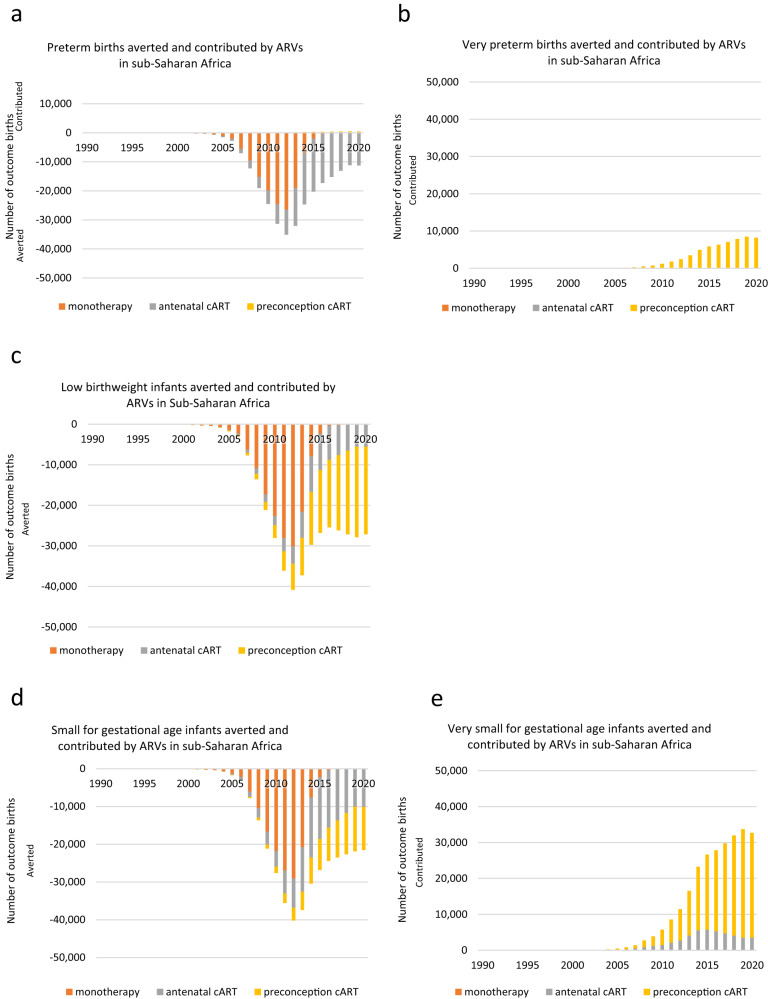
Adverse perinatal outcomes averted and contributed by ARVs in sub-Saharan Africa in 1990–2020. Numbers of adverse perinatal outcomes averted or contributed by ARVs annually between 1990 and 2020, compared to no ARVs. Positive numbers represent contribution by ARVs, i.e. if no ARVs were given, outcomes would not have occurred; negative numbers represent aversion by ART, i.e. prevented by ARVs, compared to no ARVs (Supplementary Data 1). Pregnant women living with HIV received monotherapy, cART initiated antenatally or preconception. Perinatal outcomes analysed: preterm birth (**a**), very preterm birth (**b**), low birthweight (**c**), small for gestational age (**d**), very small for gestational age (**e**). Note that not all treatment groups of WLHIV had data for all perinatal outcomes and that not all comparisons to WLHIV receiving no ARVs were statistically significant. Hence, not all categories in the legends (monotherapy, and antenatal and preconception cART) are represented in the graph for each perinatal outcome. Notably, data for very low birthweight and neonatal death was either not available or not statistically significant (Fig. 2). ARVs Antiretroviral drugs, cART Combination antiretroviral therapy.

Between 1990 and 2001, annual PTB cases attributable to HIV and ARVs increased, reaching a peak of 77,196 in 2001, mainly attributable to WLHIV receiving no ARVs (Fig. 4a, Supplementary Data 1). From 2002, annual PTB cases decreased, reaching a trough of 44,052 in 2012. Between 2000 and 2012, annual PTB cases averted by ARVs increased (compared to if no ARVs had been used), reaching a peak of 34,943 in 2012, mainly attributable to WLHIV receiving monotherapy (Fig. 5a, Supplementary Data 1). Between 2012 and 2019, annual PTB cases attributable to HIV and ARVs increased, reaching a peak of 66,728 in 2019, with the majority attributable to WLHIV receiving preconception cART (Fig. 4a, Supplementary Data 1).

**Fig. 6:**
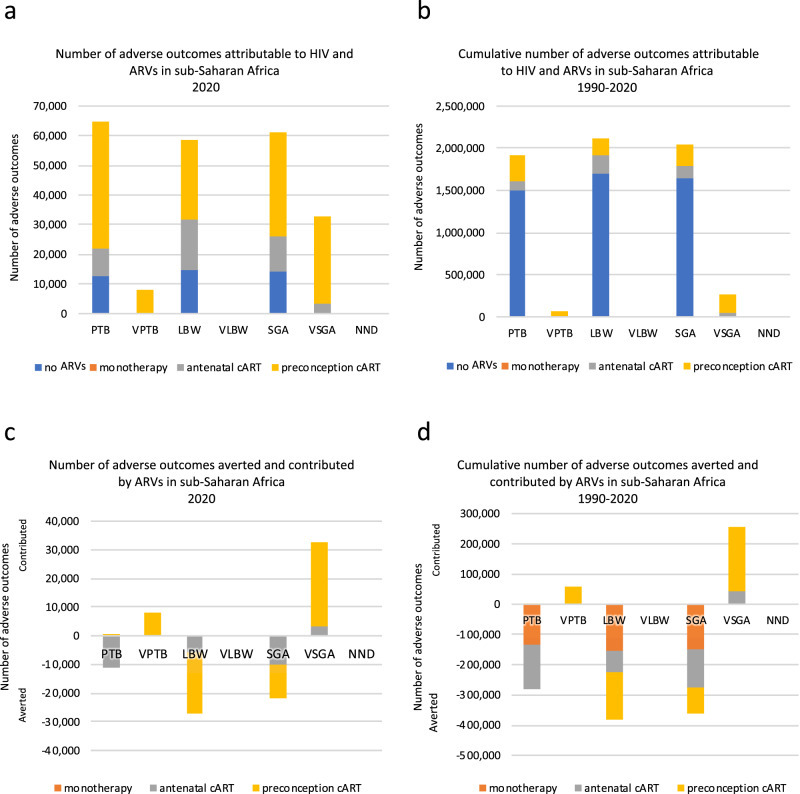
Adverse perinatal outcomes associated with HIV and ARVs, and averted and contributed by ARVs, in sub-Saharan Africa in 2020 and during 1990–2020. **a** Numbers of adverse perinatal outcomes attributable to HIV and ARVs in 2020 (Supplementary Data 1). **b** Cumulative number of adverse perinatal outcomes attributable to HIV and ARVs over 1990–2020 (Supplementary Data 1). **c** Numbers of adverse perinatal outcomes averted and contributed by ARVs in 2020 (Supplementary Data 1). **d** Cumulative number of adverse perinatal outcomes averted and contributed by ARVs over 1990–2020 (Supplementary Data 1). Note that not all treatment groups of WLHIV had data for all perinatal outcomes and that not all comparisons to HIV-negative women and WLHIV receiving no ARVs were statistically significant. Hence, not all categories in the legends (no ARVs, monotherapy, and antenatal and preconception cART) are represented in the graph for each perinatal outcome. Notably, data for very low birthweight and neonatal death was either not available or not statistically significant (Fig. 2a–d). ARVs Antiretroviral drugs, cART Combination antiretroviral therapy, HIV Human immunodeficiency virus, LBW Low birth weight, NND Neonatal death, PTB Preterm birth, SGA Small for gestational age, VPTB Very PTB, VLBW Very LBW, VSGA Very SGA.

Over the period 1990–2020, 1,921,563 PTBs were attributable to HIV and ARVs, with the majority attributable to WLHIV receiving no ARVs (1,494.370; 78%)(Fig. 6b, Supplementary Data 1). Over the same period, 276,703 PTBs were averted by ARVs, mainly attributable to WLHIV receiving monotherapy and antenatal cART. (Fig. 6d, Supplementary Data 1).

In 2020, 64,585 PTB cases were attributable to HIV and ARVs, with the majority attributable to WLHIV receiving preconception cART (42,892; 66%)(Fig. 6a, Supplementary Data 1). In 2020, ART averted 10,779 PTBs, mainly attributable to antenatal cART (Fig. 6c, Supplementary Data 1).

#### Very preterm birth

Between 2001 and 2019, annual VPTB cases attributable to HIV and ARVs increased, reaching a peak of 8497 in 2019, all attributable to WLHIV receiving preconception cART (Fig. 4b, Supplementary Data 1).

Over 1990–2020, 59,697 VPTBs were attributable to HIV and ARVs, and all were attributable to WLHIV receiving preconception cART (Fig. 6b, Supplementary Data 1), with no VPTBs averted by ARVs (Fig. 6d, Supplementary Data 1). In 2020, there were 8207 VPTB cases (Fig. 6a, Supplementary Data 1).

#### Low birthweight

Between 1990 and 2001, annual LBW cases attributable to HIV and ARVs increased, reaching a peak of 87,818 in 2001, attributable to WLHIV receiving no ARVs (Fig. 4c, Supplementary Data 1). From 2002, annual LBW cases decreased to 49,046 in 2012 (Fig. 4c, Supplementary Data 1). Between 2000 and 2012, annual cases averted by ARVs increased, reaching a peak of 40,825 in 2012, mainly attributable to WLHIV receiving monotherapy (Fig. 5c, Supplementary Data 1). Between 2012 and 2019, annual LBW cases attributable to HIV and ARVs increased, reaching a peak of 60,219 in 2019, with the majority attributable to WLHIV receiving preconception cART (Fig. 4c, Supplementary Data 1).

Over 1990–2020, 2,119,320 LBW infants were attributable to HIV and ARVs, with the majority attributable to WLHIV receiving no ARVs (1,700,122; 80%)(Fig. 6b, Supplementary Data 1). Over 1990–2020, there was a net aversion of LBW cases by ARVs of 381,612, mainly attributable to WLHIV receiving monotherapy and preconception cART. (Fig. 6d, Supplementary Data 1).

In 2020, 58,608 LBW cases were attributable to HIV and ARVs and the majority were attributable to WLHIV receiving preconception cART (26,681; 46%)(Fig. 6a, Supplementary Data 1). In 2020, there was a net aversion of 27,132 LBW cases by ARVs (Fig. 6c, Supplementary Data 1).

#### Very low birthweight

As the AR values of all the ARV categories of WLHIV were either absent or not significantly different from HIV-negative women in the meta-analyses for VLBW (Fig. 2), there were no VLBWs attributable to HIV and ARVs over the period 1990–2020 (Fig. 6).

#### Small for gestational age

Between 1990 and 2001, annual SGA cases attributable to HIV and ARVs increased, reaching a peak of 84,653 in 2001, the vast majority attributable to WLHIV receiving no ARVs (Fig. 4e, Supplementary Data 1). From 2002, annual SGA cases decreased to 46,447 cases in 2012 (Fig. 4e, Supplementary Data 1). Between 2000 and 2012, annual SGA cases averted by ARVs increased, reaching a peak of 40,183 in 2012, mainly attributable to WLHIV receiving monotherapy (Fig. 5d, Supplementary Data 1). Between 2012 and 2019, annual SGA cases attributable to HIV and ARVs increased, reaching a peak of 63,026 in 2019, the majority attributable to WLHIV receiving preconception cART (Fig. 4e, Supplementary Data 1).

Over 1990–2020, 2,049,434 SGA cases were attributable to HIV and ARVs, with the majority attributable to WLHIV receiving no ARVs (1,638,808; 80%)(Fig. 6b, Supplementary Data 1). Over 1990–2020, there was a net aversion of 361,303 cases, the majority attributable to WLHIV receiving monotherapy and antenatal cART. Figure 6d, Supplementary Data 1).

In 2020, 61,112 SGA cases were attributable to HIV and ARVs, with the majority attributable to WLHIV receiving preconception cART (35,076; 57%, Fig. 6a, Supplementary Data 1). In 2020, there was a net contribution of 21,536 SGA cases by ARVs, mainly attributable to WLHIV receiving preconception cART (Fig. 6c, Supplementary Data 1).

#### Very small for gestational age

Between 2000 and 2019, annual VSGA cases increased, reaching a peak of 33,714 in 2019, mainly attributable to WLHIV receiving preconception cART (Fig. 4f, Supplementary Data 1).

Over 1990–2020, 257,902 VSGA cases were attributable to HIV and ARVs, with the majority attributable to WLHIV receiving preconception cART (212,359; 82%)(Fig. 6b, Supplementary Data 1), while no VSGA cases were averted by ARVs (Fig. 6d, Supplementary Data 1).

In 2020, 32,706 VSGA cases were attributable to HIV and ARVs, with the majority attributable to WLHIV receiving preconception cART (29,196; 89%)(Fig. 6a, Supplementary Data 1).

#### Neonatal death

As the AR values of all the ARV categories of WLHIV were either absent or not significantly different from HIV-negative women in the meta-analyses for NND (Fig. 2), there were no NNDs attributable to HIV and ARVs over the period 1990–2020 (Fig. 6).

### Country analysis

**Fig. 7:**
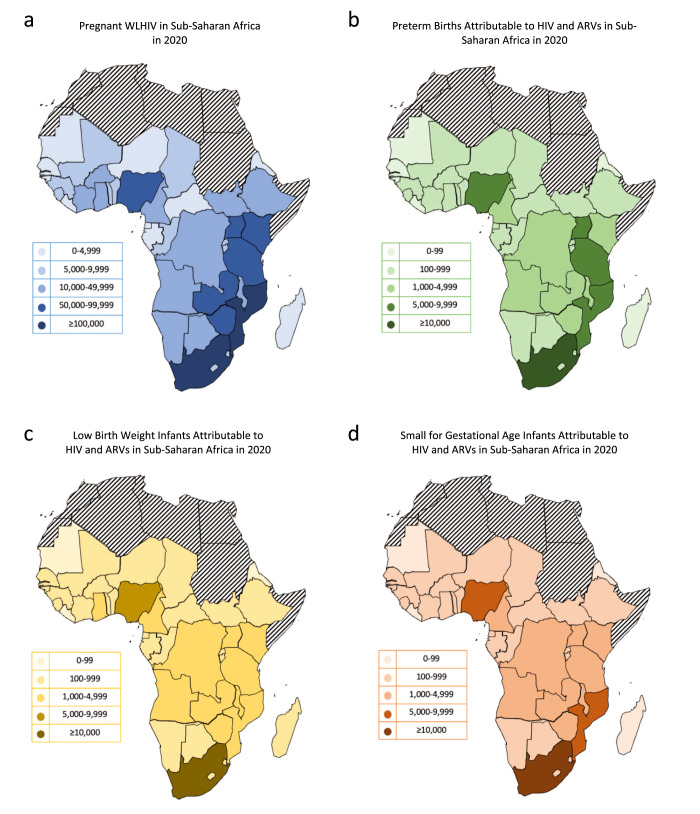
Pregnant women living with HIV and adverse perinatal outcomes associated with HIV and ARVs in countries in sub-Saharan Africa in 2020. **a** Number of pregnant women living with HIV in countries in sub-Saharan Africa in 2020 (Supplementary Data 1). **b** Number of preterm births in countries in sub-Saharan Africa in 2020 (Supplementary Data 1). **c** Number of low birthweight infants in countries in sub-Saharan Africa in 2020 (Supplementary Data 1). **d** Number of small for gestational age infants in countries in sub-Saharan Africa in 2020 (Supplementary Data 1). ARVs Antiretroviral drugs, WLHIV Women living with HIV.

In 2020, the country with the highest number of pregnant WLHIV was South Africa (309,083; 26%), followed by Mozambique (107,148; 9%), Tanzania (91,323; 8%), Uganda (90,941; 8%), and Nigeria (83,348; 7%) (Fig. 7a, Supplementary Data 1). South Africa also had the highest number of PTBs attributable to HIV and ARVs (16,705; 26%), followed by Mozambique (5374; 8%), Tanzania (5075; 8%), Nigeria (5071; 8%) and Uganda (5040; 8%) (Fig. 7b, Supplementary Data 1). South Africa had the highest number of LBWs attributable to HIV and ARVs (14,196; 24%), followed by Nigeria (5012; 9%) (Fig. 7c, Supplementary Data 1). Finally, South Africa also had the highest number of SGAs attributable to HIV and ARVs (15,248; 25%), followed by Nigeria (5112; 8%) and Mozambique (5022; 8%) (Fig. 7d, Supplementary Data 1).

## Discussion

To the best of our knowledge this is the first study estimating the burden of adverse perinatal outcomes associated with HIV and ARVs in sub-Saharan Africa. We found that WLHIV receiving no ARVs or cART initiated antenatally or preconception, but not monotherapy, are at increased risk of PTB, LBW and SGA, compared to HIV-negative women. WLHIV receiving preconception cART had the highest risk of PTB, VPTB and VSGA, and WLHIV receiving no ARVs had the highest risk of LBW and SGA. During the period 1990–2020, a total of 1,921,563 PTBs, 59,697 VPTBs, 2,119,320 LBWs, 2,049,434 SGAs and 257,902 VSGAs were attributable to HIV and ARVs in sub-Saharan Africa, mainly among WLHIV receiving no ARVs, while monotherapy and preconception and antenatal cART averted many adverse outcomes. In the year 2020, 64,585 PTBs, 8207 VPTBs, 58,608 LBWs, 61,112 SGAs and 32,706 VSGAs were attributable to HIV and ARVs, the majority among WLHIV receiving preconception cART. In 2020, South Africa had the highest number of PTBs, LBWs and SGAs attributable to HIV and ARVs.

The finding that WLHIV receiving no ARVs had an increased risk of adverse perinatal outcomes compared to HIV-negative women is consistent with a previous meta-analysis^16^. WLHIV receiving monotherapy had similar outcomes to HIV-negative women, which might reflect good general health and high CD4^+^ T cell counts in this group. In contrast, WLHIV on cART had higher risks of adverse outcomes compared to HIV-negative women, indicating that cART does not reverse the effect of HIV infection on adverse perinatal outcomes, or that cART does ameliorate effects of HIV infection but has other adverse drug effects. Furthermore, preconception cART initiation was associated with higher risks of adverse perinatal outcomes than antenatal cART initiation, a finding consistent with a previous meta-analysis that directly compared preconception and antenatal cART^23^. This finding might indicate that longer cART exposure during pregnancy is associated with a higher risk of adverse perinatal outcomes. However, it is difficult to exclude that selection bias has influenced these results as WLHIV receiving preconception cART have more time to experience adverse perinatal outcomes compared to WLHIV who initiate cART antenatally, often late in pregnancy^64^. The same timing considerations apply to the comparison of preconception cART with antenatal monotherapy. Moreover, indication bias could have affected studies taking place before the change in treatment recommendations in 2013^12^, as preconception cART would have been initiated for maternal health reasons, whereas antenatal monotherapy or cART could have been initiated for prevention of vertical HIV transmission (at high CD4^+^ T cell count) or antenatal cART for maternal health (at lower CD4^+^ T cell count). Additionally, characteristics of WLHIV receiving antenatal cART may differ from WLHIV starting cART preconception, e.g. newly diagnosed with HIV or having limited access to healthcare. Moreover, HIV-negative women may have characteristics and risk factors for adverse perinatal outcomes that differ from WLHIV. Taken together, confounders may be present in the comparator groups in our analyses (WLHIV receiving different ARVs and HIV negative women), but we were unable to correct for these in our meta-analysis. While adjusting for confounders is important to establish causality, unadjusted estimates are valid for our analysis to estimate the burden of disease associated with WLHIV on different ARV regimens. Although the associations found are consistent, causality cannot be inferred from our observational data.

We observed changes in the numbers of adverse perinatal outcomes attributable to HIV and ARVs in sub-Saharan Africa during 1990–2020, which resulted from changes in the numbers of WLHIV and the ARV regimens received, which are associated with different risks of adverse perinatal outcomes. The proportion of WLHIV receiving no ARVs decreased between 2002–2020, which is reflected in the decrease in the number of annual cases of PTB, LBW and SGA attributable to this group of WLHIV. Between 2002 and 2012 there was a decrease in the number of annual cases of PTB, LBW and SGA, which was attributable to WLHIV receiving monotherapy, who are associated with a lower risk of adverse perinatal outcomes compared to WLHIV receiving no ARVs. However, monotherapy was no longer recommended from 2013, resulting in a sharp decrease in the numbers of WLHIV receiving monotherapy from 2013 onwards. Around the same time, the number of WLHIV receiving antenatal and preconception cART increased, resulting in an increase of annual cases of PTB, VPTB, LBW, SGA and VSGA between 2013 and 2019. In 2020, the majority of adverse perinatal outcomes attributable to HIV and ARVs in sub-Saharan Africa were attributable to WLHIV receiving preconception cART, which form the largest group of pregnant WLHIV and are associated with elevated risks of adverse perinatal outcomes.

This study has several strengths. To our knowledge, this is the largest study on this topic to date, reporting on a range of adverse perinatal outcomes associated with WLHIV, including 399,558 women from 34 studies in 14 countries, with 24 studies (including 181,803 women) reporting on PTB, 22 studies (246,181 women) reporting on LBW, and 13 studies (170,735 women) reporting on SGA. For the first time, we were able to estimate the burden of adverse perinatal outcomes associated with HIV and ARVs in sub-Saharan Africa in 1990–2020, by combining attributable risks of adverse perinatal outcomes associated with different types of ARVs with the annual numbers of WLHIV receiving each form of ARVs in each country in sub-Saharan Africa. Our study was conducted according to Cochrane guidelines^24^, with exposures and outcomes clearly predefined to minimise misclassification bias and promote consistency across studies. Subgroup and sensitivity analyses supported our main findings. The systematic review and meta-analysis were limited to countries in sub-Saharan Africa, lending external validity to our findings. Where applicable, the Peters’ test confirmed an absence of publication bias, and the systematic review was reported according to PRISMA guidelines^25^.

This study has some limitations. All studies included were observational and therefore associated with an increased risk of bias. However, these cohort studies may be more representative of events in the real world compared to the few RCTs conducted in this field to date, which often have restricted participant inclusion criteria (higher CD4^+^ T cell counts) and in which ARVs are initiated during pregnancy, often in the second or third trimester, providing no evidence regarding preconception ARV initiation^19^. For WLHIV receiving monotherapy only three outcomes were assessed (PTB, LBW, and SGA) in few studies ( < 5 studies each), though the numbers of women analysed were high ( > 40,000 women each). Despite the clear associations of PTB and SGA with neonatal mortality^65^, our findings for PTB, LBW and SGA did not translate into an effect on neonatal death, for which the data were highly heterogeneous for no ARVs (4 studies) and extremely limited for preconception and antenatal cART (1 study each). Few studies reported on VPTB (4 studies), VLBW (1), and VSGA (4); the effect estimates observed for these outcomes were smaller than those observed for PTB, LBW, and SGA, respectively, leading to smaller numbers of these more severe outcomes, as would be expected. Overall the evidence available for VPTB, VLBW, VSGA and NND was limited, restricting our ability to draw conclusions for these outcomes. Vertical HIV transmission was not one of the outcomes of interest in this study.

We were unable to perform a more detailed analysis of the potential role of cART regimens containing different antiretroviral drugs or classes of drugs, as data on specific cART regimens received by pregnant WLHIV in different countries during 1990–2020 was not available. The evidence on the association of different cART regimens with perinatal outcomes is conflicting^17,66^. Among the cART regimens assessed in RCTs, cART regimens containing the protease inhibitor (PI) lopinavir/ritonavir (LPV/r) were associated with an increased risk of spontaneous PTB compared to zidovudine/lamivudine/abacavir (ZDV/3TC/ABC; a nucleoside reverse transcriptase inhibitor [NRTI] cART regimen which is no longer recommended), but no other significant differences in perinatal outcomes between the cART regimens assessed were found^19^. A recent large meta-analysis of cohort studies showed that PI-based cART is associated with an increased risk of SGA and VSGA, but not PTB or any other perinatal outcomes, compared with non-PI-based cART. Additionally, no significant differences between different PI drugs were found^67^. WHO guidance currently recommends dolutegravir (DTG)-containing cART as the preferred first-line therapy^9^. While a retrospective cohort study reported that perinatal outcomes were comparable between WLHIV receiving DTG-based and efavirenz (EFV)-based cART^63,68^, a recent RCT reported that a regimen containing DTG, emtricitabine (FTC), and tenofovir alafenamide fumarate (TAF) initiated antenatally had the lowest rate of adverse pregnancy outcomes, compared to DTG/FTC/tenofovir disoproxil fumarate (TDF) and EFV/FTC/TDF^69^.

The biological mechanisms contributing to the associations between HIV, ARVs and adverse perinatal outcomes remain unclear, in part due to conflicting epidemiological data^67,70^. HIV-infection may impact the immunological programme of pregnancy, by depletion of CD4^+^ T cells and chronic immune activation^71^. Several innate immune cells, including innate lymphoid cells and mucosal associated invariant T cells, are also depleted during early HIV infection and fail to recover with cART, and may be associated with increased risk of adverse perinatal outcomes^72,73^. WLHIV receiving cART were reported to have distinct systemic cytokine profiles throughout pregnancy, compared to HIV-negative women, which may be associated with SGA^74^. Additionally, PIs included in cART regimens may inhibit progesterone production by the placenta^70^, and reduced progesterone levels were associated with increased risk of SGA^75^. Interestingly, a recent RCT of progesterone supplementation in pregnant WLHIV on cART showed a reduction in VSGA, but not PTB or stillbirth^76^. In summary, the available data are limited and complex, and highlight the need for more mechanistic studies.

Estimating the burden of diseases and risk factors is crucial to determine the magnitude and trends of health problems, in order to enable appropriate allocation of resources for research and public health policy, and monitor their impact^1^. Most countries in sub-Saharan Africa are not on track to reach SDG Target 3.2 to reduce neonatal and child mortality^3^. We have shown that HIV and ARVs contribute significantly to the burden of adverse perinatal outcomes in sub-Saharan Africa in 1990–2020. An increasing proportion of pregnant WLHIV receive preconception cART, which has clear benefits for maternal health and prevention of HIV transmission to the child. However, we have shown that preconception cART is also associated with the highest risks of adverse perinatal outcomes among WLHIV. Further studies are therefore urgently needed to determine the optimal cART regimen(s) to minimise adverse perinatal outcomes, and develop preventative and therapeutic interventions to improve perinatal outcomes among WLHIV.

### Supplementary information

Supplementary Data 1

Supplementary Data 2

Supplementary Data 3

Supplementary Data 4

Description of Additional Supplementary Files

Supplementary Information

Reporting Summary

## Data Availability

All data generated or analysed during this study are included in this published article and its Supplementary Information files. Source data for the figures are available in the Supplementary Data files and in the Supplementary Information. The list of included studies is available in Supplementary Data 3.
