# Publisher Correction: Adverse perinatal outcomes attributable to HIV in sub-Saharan Africa from 1990 to 2020: systematic review and meta-analyses

**DOI:** 10.1038/s43856-023-00415-5

**Published:** 2023-12-12

**Authors:** Claudia Murray, Clara Portwood, Harriet Sexton, Mary Kumarendran, Zoe Brandon, Shona Kirtley, Joris Hemelaar

**Affiliations:** 1 National Perinatal Epidemiology Unit, Oxford Population Health, Nuffield Department of Population Health, University of Oxford, Oxford, UK; 2 Centre for Statistics in Medicine, Nuffield Department of Orthopaedics, Rheumatology and Musculoskeletal Sciences, University of Oxford, Oxford, UK

**Keywords:** Epidemiology, Paediatric research

Correction to: *Communications Medicine* 10.1038/s43856-023-00331-8, published online 22 July 2023.

There were errors in the *y*-axis labels of Figures 3 and 4 of this article. For both figures, commas were positioned or added incorrectly in the numbers on the *y*-axis. These errors have now been corrected in both the PDF and HTML versions of this article.

