# Supplementary material for: Adverse perinatal outcomes attributable to HIV in sub-Saharan Africa from 1990 to 2020: Systematic review and meta-analyses
Source: Commun Med (Lond). 2023 Jul 22;3:103. doi: 10.1038/s43856-023-00331-8 (PMC10363130; doi:10.1038/s43856-023-00331-8)
Supplement: Supplementary file 5 — Description of Additional Supplementary Files [file 43856_2023_331_MOESM5_ESM.pdf]

Description of Additional Supplementary Files

File Name: Supplementary Data 1

Description: Adverse perinatal outcomes data and confidence interval tables

File Name: Supplementary Data 2

Description: Treatment regimens

File Name: Supplementary Data 3

Description: Study characteristics.

Treatment of WLHIV: no treatment (no ARVs), zidovudine monotherapy (monotherapy), cART
initiated antenatally (antenatal cART) or preconception (preconception cART).
* Details on the inclusion of twins, recruitment centre, urban/rural setting, deliveries at home/hospital, smoking, alcohol use, and IDU were sought and reported here if provided by each study.
Abbreviations: ARVs = antiretroviral drugs, cART = combination ART, IDU = illicit drug use, low birth weight (LBW), neonatal death (NND), preterm birth (PTB), small for gestational age (SGA), very LBW (VLBW), very PTB (VPTB), very SGA (VSGA). WLHIV = women living with human immunodeficiency virus

File Name: Supplementary Data 4

Description: Quality assessments
