# Supplementary material for: Adverse perinatal outcomes attributable to HIV in sub-Saharan Africa from 1990 to 2020: Systematic review and meta-analyses
Source: Commun Med (Lond). 2023 Jul 22;3:103. doi: 10.1038/s43856-023-00331-8 (PMC10363130; doi:10.1038/s43856-023-00331-8)
Supplement: Supplementary file 6 — Supplementary Information [file 43856_2023_331_MOESM6_ESM.pdf]

Supplementary Information

Adverse pregnancy outcomes attributable to HIV in sub-Saharan Africa in 1990-2020: systematic review and meta-analyses.

Claudia Murray, Clara Portwood, Harriet Sexton, Mary Kumarendran, Zoe Brandon, Shona Kirtley, Joris Hemelaar

Index

Supplementary Note 1: Literature Search Strategies 2
Supplementary Note 2: Adapted Newcastle-Ottawa quality assessment tool 17
Supplementary Note 3: Classification of studies according to quality assessment 18
Supplementary Figures 1-7: Women living with HIV without treatment vs HIV-negative women 19-22
Supplementary Figures 8-10: Women living with HIV on monotherapy vs HIV-negative women 23-24
Supplementary Figures 11-17: Women living with HIV on antenatal cART vs HIV-negative women 25-28
Supplementary Figures 18-24: Women living with HIV on preconception cART vs HIV-negative women 29-32
Supplementary Figure 25: Subgroup analysis of women living with HIV receiving no treatment 33
Supplementary Figure 26: Subgroup analysis of women living with HIV receiving monotherapy 34
Supplementary Figure 27: Subgroup analyses of women living with HIV receiving antenatal cART 35
Supplementary Figure 28: Subgroup analysis of women living with HIV receiving preconception cART 36

Supplementary Note 1

Literature Search Strategies

Search strategy for "pregnancy outcome AND HIV OR antiretroviral therapy"
Database and platform: Pubmed (via https://www.ncbi.nlm.nih.gov/pubmed/)
Latest search date: 20 April 2020.

1. Pregnancy Outcome [Mesh] OR Pregnancy Complications, Infectious [Mesh] OR "pregnancy outcome*" [tiab] OR "pregnancy complication*" [tiab] OR pregnancy consequence* [tiab] OR "pregnancy characteristic" [tiab] OR "pregnancy characteristics" [tiab] OR pregnancy event* [tiab] OR pregnancy result* [tiab] OR pregnancy problem* [tiab] OR pregnancy morbidit* [tiab] OR pregnancy sequelae [tiab]

2. gestational outcome* [tiab] OR gestational complication* [tiab] OR gestational consequence* [tiab] OR gestational characteristic* [tiab] OR gestational event* [tiab] OR gestational result* [tiab] OR gestational problem* [tiab] OR gestational morbidit* [tiab] OR gestational sequelae [tiab]

3. "fetal outcome" [tiab] OR "fetal outcomes" [tiab] OR "fetal complication" [tiab] OR "fetal complications" [tiab] OR fetal consequence* [tiab] OR fetal characteristic* [tiab] OR fetal event* [tiab] OR fetal result* [tiab] OR fetal problem* [tiab] OR fetal morbidit* [tiab] OR fetal sequelae [tiab]

4. foetal outcome* [tiab] OR foetal complication* [tiab] OR foetal consequence* [tiab] OR foetal characteristic* [tiab] OR foetal event* [tiab] OR foetal result* [tiab] OR foetal problem* [tiab] OR foetal morbidit* [tiab] OR foetal sequelae [tiab]

5. "obstetric outcome" [tiab] OR "obstetric outcomes" [tiab] OR "obstetric complication" [tiab] OR "obstetric complications" [tiab] OR obstetric consequence* [tiab] OR obstetric characteristic* [tiab] OR obstetric event* [tiab] OR obstetric result* [tiab] OR "obstetric problem" [tiab] OR "obstetric problems" [tiab] OR obstetric morbidit* [tiab] OR obstetric sequelae [tiab]

6. obstetrical outcome* [tiab] OR "obstetrical complication" [tiab] OR "obstetrical complications" [tiab] OR obstetrical consequence* [tiab] OR obstetrical characteristic* [tiab] OR obstetrical event* [tiab] OR obstetrical result* [tiab] OR "obstetrical problem" [tiab] OR "obstetrical problems" [tiab] OR obstetrical morbidit* [tiab] OR obstetrical sequelae [tiab]

7. "labor outcome" [tiab] OR "labor outcomes" [tiab] OR "labor complication" [tiab] OR "labor complications" [tiab] OR "labor consequence" [tiab] OR "labor consequences" [tiab] OR labor characteristic* [tiab] OR labor event* [tiab] OR labor result* [tiab] OR labor problem* [tiab] OR labor morbidit* [tiab] OR labor sequelae [tiab]

8. labour outcome* [tiab] OR labour complication* [tiab] OR "labour consequence" [tiab] OR "labour consequences" [tiab] OR labour characteristic* [tiab] OR labour event* [tiab] OR labour result* [tiab] OR labour problem* [tiab] OR labour morbidit* [tiab] OR labour sequelae [tiab]

9. "birth outcome" [tiab] OR "birth outcomes" [tiab] OR birth complication* [tiab] OR birth consequence* [tiab] OR birth characteristic* [tiab] OR birth event* [tiab] OR birth result* [tiab] OR birth problem* [tiab] OR birth morbidit* [tiab] OR birth sequelae [tiab]

10. delivery outcome* [tiab] OR delivery complication* [tiab] OR delivery consequence* [tiab] OR delivery characteristic* [tiab] OR delivery event* [tiab] OR delivery result* [tiab] OR delivery problem* [tiab] OR delivery morbidit* [tiab] OR delivery sequelae [tiab]

11. neonate outcome* [tiab] OR neonate complication* [tiab] OR neonate consequence* [tiab] OR neonate characteristic* [tiab] OR neonate event* [tiab] OR neonate result* [tiab] OR neonate problem* [tiab] OR neonate morbidit* [tiab] OR neonate sequelae [tiab]

12. newborn outcome* [tiab] OR newborn complication* [tiab] OR newborn consequence* [tiab] OR newborn characteristic* [tiab] OR newborn event* [tiab] OR newborn result* [tiab] OR newborn problem* [tiab] OR newborn morbidit* [tiab] OR newborn sequelae [tiab]

13. new-born outcome* [tiab] OR new-born complication* [tiab] OR new-born consequence* [tiab] OR new-born characteristic* [tiab] OR new-born event* [tiab] OR new-born result* [tiab] OR new-born problem* [tiab] OR new-born morbidit* [tiab] OR new-born sequelae [tiab]

14. new born outcome* [tiab] OR new born complication* [tiab] OR new born consequence* [tiab] OR new born characteristic* [tiab] OR new born event* [tiab] OR new born result* [tiab] OR new born problem* [tiab] OR new born morbidit* [tiab] OR new born sequelae [tiab]

15. infant outcome* [tiab] OR infant complication* [tiab] OR infant consequence* [tiab] OR infant characteristic* [tiab] OR infant event* [tiab] OR infant result* [tiab] OR infant problem* [tiab] OR infant morbidit* [tiab] OR infant sequelae [tiab]

16. reproductive outcome* [tiab] OR reproductive complication* [tiab] OR reproductive consequence* [tiab] OR reproductive characteristic* [tiab] OR reproductive event* [tiab] OR reproductive result* [tiab] OR reproductive problem* [tiab] OR reproductive morbidit* [tiab] OR reproductive sequelae [tiab]

17. prelabour outcome* [tiab] OR prelabour complication* [tiab] OR prelabour consequence* [tiab] OR prelabour characteristic* [tiab] OR prelabour event* [tiab] OR prelabour result* [tiab] OR prelabour problem* [tiab] OR prelabour morbidit* [tiab] OR prelabour sequelae [tiab]

18. prelabor outcome* [tiab] OR prelabor complication* [tiab] OR prelabor consequence* [tiab] OR prelabor characteristic* [tiab] OR prelabor event* [tiab] OR prelabor result* [tiab] OR prelabor problem* [tiab] OR prelabor morbidit* [tiab] OR prelabor sequelae [tiab]

19. pre-labour outcome* [tiab] OR pre-labour complication* [tiab] OR pre-labour consequence* [tiab] OR pre-labour characteristic* [tiab] OR pre-labour event* [tiab] OR pre-labour result* [tiab] OR pre-labour problem* [tiab] OR pre-labour morbidit* [tiab] OR pre-labour sequelae [tiab]

20. pre-labor outcome* [tiab] OR pre-labor complication* [tiab] OR pre-labor consequence* [tiab] OR pre-labor characteristic* [tiab] OR pre-labor event* [tiab] OR pre-labor result* [tiab] OR pre-labor problem* [tiab] OR pre-labor morbidit* [tiab] OR pre-labor sequelae [tiab]

21. intrauterine outcome* [tiab] OR intrauterine complication* [tiab] OR intrauterine consequence* [tiab] OR intrauterine characteristic* [tiab] OR intrauterine event* [tiab] OR intrauterine result* [tiab] OR intrauterine problem* [tiab] OR intrauterine morbidit* [tiab] OR intrauterine sequelae [tiab]

22. intra-uterine outcome* [tiab] OR intra-uterine complication* [tiab] OR intra-uterine consequence* [tiab] OR intra-uterine characteristic* [tiab] OR intra-uterine event* [tiab] OR intra-uterine result* [tiab] OR intra-uterine problem* [tiab] OR intra-uterine morbidit* [tiab] OR intra-uterine sequelae [tiab]

23. antenatal outcome* [tiab] OR antenatal complication* [tiab] OR antenatal consequence* [tiab] OR antenatal characteristic* [tiab] OR antenatal event* [tiab] OR antenatal result* [tiab] OR antenatal problem* [tiab] OR antenatal morbidit* [tiab] OR antenatal sequelae [tiab]

24. ante-natal outcome* [tiab] OR ante-natal complication* [tiab] OR ante-natal consequence* [tiab] OR ante-natal characteristic* [tiab] OR ante-natal event* [tiab] OR ante-natal result* [tiab] OR ante-natal problem* [tiab] OR ante-natal morbidit* [tiab] OR ante-natal sequelae [tiab]

25. prenatal outcome* [tiab] OR prenatal complication* [tiab] OR prenatal consequence* [tiab] OR prenatal characteristic* [tiab] OR prenatal event* [tiab] OR prenatal result* [tiab] OR prenatal problem* [tiab] OR prenatal morbidit* [tiab] OR prenatal sequelae [tiab]

26. pre-natal outcome* [tiab] OR pre-natal complication* [tiab] OR pre-natal consequence* [tiab] OR pre-natal characteristic* [tiab] OR pre-natal event* [tiab] OR pre-natal result* [tiab] OR pre-natal problem* [tiab] OR pre-natal morbidit* [tiab] OR pre-natal sequelae [tiab]

27. perinatal outcome* [tiab] OR perinatal complication* [tiab] OR perinatal consequence* [tiab] OR perinatal characteristic* [tiab] OR perinatal event* [tiab] OR perinatal result* [tiab] OR perinatal problem* [tiab] OR perinatal morbidit* [tiab] OR perinatal sequelae [tiab]
28. peri-natal outcome* [tiab] OR peri-natal complication* [tiab] OR peri-natal consequence* [tiab] OR peri-natal characteristic* [tiab] OR peri-natal event* [tiab] OR peri-natal result* [tiab] OR peri-natal problem* [tiab] OR peri-natal morbidit* [tiab] OR peri-natal sequelae [tiab]

29. neonatal outcome* [tiab] OR neonatal complication* [tiab] OR neonatal consequence* [tiab] OR neonatal characteristic* [tiab] OR neonatal event* [tiab] OR neonatal result* [tiab] OR neonatal problem* [tiab] OR neonatal morbidit* [tiab] OR neonatal sequelae [tiab]

30. neo-natal outcome* [tiab] OR neo-natal complication* [tiab] OR neo-natal consequence* [tiab] OR neo-natal characteristic* [tiab] OR neo-natal event* [tiab] OR neo-natal result* [tiab] OR neo-natal problem* [tiab] OR neo-natal morbidit* [tiab] OR neo-natal sequelae [tiab]

31. postnatal outcome* [tiab] OR postnatal complication* [tiab] OR postnatal consequence* [tiab] OR postnatal characteristic* [tiab] OR postnatal event* [tiab] OR postnatal result* [tiab] OR postnatal problem* [tiab] OR postnatal morbidit* [tiab] OR postnatal sequelae [tiab]

32. post-natal outcome* [tiab] OR post-natal complication* [tiab] OR post-natal consequence* [tiab] OR post-natal characteristic* [tiab] OR post-natal event* [tiab] OR post-natal result* [tiab] OR post-natal problem* [tiab] OR post-natal morbidit* [tiab] OR post-natal sequelae [tiab]

33. antepartum outcome* [tiab] OR antepartum complication* [tiab] OR antepartum consequence* [tiab] OR antepartum characteristic* [tiab] OR antepartum event* [tiab] OR antepartum result* [tiab] OR antepartum problem* [tiab] OR antepartum morbidit* [tiab] OR antepartum sequelae [tiab]

34. ante-partum outcome* [tiab] OR ante-partum complication* [tiab] OR ante-partum consequence* [tiab] OR ante-partum characteristic* [tiab] OR ante-partum event* [tiab] OR ante-partum result* [tiab] OR ante-partum problem* [tiab] OR ante-partum morbidit* [tiab] OR ante-partum sequelae [tiab]

35. intrapartum outcome* [tiab] OR intrapartum complication* [tiab] OR intrapartum consequence* [tiab] OR intrapartum characteristic* [tiab] OR intrapartum event* [tiab] OR intrapartum result* [tiab] OR intrapartum problem* [tiab] OR intrapartum morbidit* [tiab] OR intrapartum sequelae [tiab]

36. intra-partum outcome* [tiab] OR intra-partum complication* [tiab] OR intra-partum consequence* [tiab] OR intra-partum characteristic* [tiab] OR intra-partum event* [tiab] OR intra-partum result* [tiab] OR intra-partum problem* [tiab] OR intra-partum morbidit* [tiab] OR intra-partum sequelae [tiab]

37. peripartum outcome* [tiab] OR peripartum complication* [tiab] OR peripartum consequence* [tiab] OR peripartum characteristic* [tiab] OR peripartum event* [tiab] OR peripartum result* [tiab] OR peripartum problem* [tiab] OR peripartum morbidit* [tiab] OR peripartum sequelae [tiab]
38. peri-partum outcome* [tiab] OR peri-partum complication* [tiab] OR peri-partum consequence* [tiab] OR peri-partum characteristic* [tiab] OR peri-partum event* [tiab] OR peri-partum result* [tiab] OR peri-partum problem* [tiab] OR peri-partum morbidit* [tiab] OR peri-partum sequelae [tiab]

39. postpartum outcome* [tiab] OR postpartum complication* [tiab] OR postpartum consequence* [tiab] OR postpartum characteristic* [tiab] OR postpartum event* [tiab] OR postpartum result* [tiab] OR postpartum problem* [tiab] OR postpartum morbidit* [tiab] OR postpartum sequelae [tiab]

40. post-partum outcome* [tiab] OR post-partum complication* [tiab] OR post-partum consequence* [tiab] OR post-partum characteristic* [tiab] OR post-partum event* [tiab] OR post-partum result* [tiab] OR post-partum problem* [tiab] OR post-partum morbidit* [tiab] OR post-partum sequelae [tiab]

41. #1 or #2 or #3 or #4 or #5 or #6 or #7 or #8 or #9 or #10 or #11 or #12 or #13 or #14 or #15 or #16 or #17 or #18 or #19 or #20 or #21 or #22 or #23 or #24 or #25 or #26 or #27 or #28 or #29 or #30 or #31 or #32 or #33 or #34 or #35 or #36 or #37 or #38 or #39 or #40

42. HIV [Mesh] OR HIV Seropositivity [Mesh] OR HIV Infections [Mesh] OR HIV-2 [Mesh] OR HIV-1 [Mesh] OR AIDS Serodiagnosis [Mesh] OR Acquired Immunodeficiency Syndrome [Mesh] OR AIDS Arteritis, Central Nervous System [Mesh] OR AIDS-Associated Nephropathy [Mesh] OR AIDS

Dementia Complex [Mesh] OR AIDS-Related Opportunistic Infections [Mesh] OR Lymphoma, AIDS-Related [Mesh]

43. HIV [tiab] OR HIV-1 [tiab] OR HIV-type-1 [tiab] OR ìHTLV IIIî [tiab] OR HTLV-III [tiab] OR ìHTLV type IIIî [tiab] OR HTLV-type-III [tiab] OR LAV [tiab] OR HTLV-III-LAV [tiab] OR LAV-HTLV-III [tiab] OR HIV-2 [tiab] OR HIV-type-2 [tiab] OR HIV-II [tiab] OR HTLV-IV [tiab] OR LAV-2 [tiab]

44. HIV-positive [tiab] OR HIV-1-positive [tiab] OR HIV-2-positive [tiab] OR HIV-infected [tiab] OR HIV-1-infected [tiab] OR HIV-type-1-infected [tiab] OR ìHTLV III-infectedî [tiab] OR HTLV-III-infected [tiab] OR ìHTLV type III-infectedî [tiab] OR HTLV-type-III-infected [tiab] OR LAV-infected [tiab]

45. HTLV-III-LAV-infected [tiab] OR LAV-HTLV-III-infected [tiab] OR HIV-2-infected [tiab] OR HIV-type-2-infected [tiab] OR HIV-II-infected [tiab] OR HTLV-IV-infected [tiab] OR LAV-2-infected [tiab] OR HIV-infection* [tiab] OR HIV-1-infection* [tiab] OR HIV-type-1-infection* [tiab] OR ìHTLV III-infection*î [tiab] OR HTLV-III-infection* [tiab] OR HTLV type III-infection* [tiab] OR HTLV-type-III-infection* [tiab]

46. LAV-infection* [tiab] OR HTLV-III-LAV-infection* [tiab] OR LAV-HTLV-III-infection* [tiab] OR HIV-2-infection* [tiab] OR HIV-type-2-infection* [tiab] OR HIV-II-infection* [tiab] OR HTLV-IV-infection* [tiab] OR LAV-2-infection* [tiab] OR Human Immunodeficiency Virus* [tiab] OR ìHuman Immune Deficiency Virus*î [tiab]

47. ìHuman T Cell Lymphotropic Virus Type IIIî [tiab] OR ìHuman T-Cell Lymphotropic Virus Type IIIî [tiab] OR ìHuman T Lymphotropic Virus Type IIIî [tiab] OR ìHuman T-Lymphotropic Virus Type IIIî [tiab] OR ìHuman T Lymphotropic Virus Type IVî [tiab] OR ìHuman T-Lymphotropic Virus Type IVî [tiab] OR ìHuman T Cell Leukemia Virus Type IIIî [tiab] OR ìHuman T-Cell Leukemia Virus Type IIIî [tiab]

48. ìLymphadenopathy-Associated Virus*î [tiab] OR ìLymphadenopathy Associated Virus*î [tiab] OR AIDS [tiab] OR ìAcquired Immune Deficiency Syndromeî [tiab] OR ìAcquired Immunodeficiency Syndromeî [tiab]

49. #42 or #43 or #44 or #45 or #46 or #47 or #489. #42 or #43 or #44 or #45 or #46 or #47 or #48

50. Anti-HIV Agents [Mesh] OR HIV Fusion Inhibitors [Mesh] OR HIV Integrase Inhibitors [Mesh] OR HIV Protease Inhibitors [Mesh]

51. antiretrovirals [tiab] OR ìantiretroviral treatment*î [tiab] OR ìantiretroviral therapyî [tiab] OR ìantiretroviral therapiesî [tiab] OR ìantiretroviral regimen*î [tiab] OR ìantiretroviral drug*î [tiab] OR ìantiretroviral agent*î [tiab]

52. anti-retrovirals [tiab] OR ìanti-retroviral treatment*î [tiab] OR ìanti-retroviral therapyî [tiab] OR ìanti-retroviral therapiesî [tiab] OR ìanti-retroviral regimen*î [tiab] OR ìanti-retroviral drug*î [tiab] OR ìanti-retroviral agent*î [tiab]

53. antivirals [tiab] OR ìantiviral treatment*î [tiab] OR ìantiviral therapyî [tiab] OR ìantiviral therapiesî [tiab] OR ìantiviral regimen*î [tiab] OR ìantiviral drug*î [tiab] OR ìantiviral agent*î [tiab]

54. anti-virals [tiab] OR ìanti-viral treatment*î [tiab] OR ìanti-viral therapyî [tiab] OR ìanti-viral therapiesî [tiab] OR ìanti-viral regimen*î [tiab] OR ìanti-viral drug*î [tiab] OR ìanti-viral agent*î [tiab]

55. ìanti-HIV treatment*î [tiab] OR ìanti-HIV therapyî [tiab] OR ìanti-HIV therapiesî [tiab] OR ìanti-HIV regimen*î [tiab] OR ìanti-HIV drug*î [tiab] OR ìanti-HIV agent*î [tiab]

56. ìHIV treatment*î [tiab] OR ìHIV therapyî [tiab] OR ìHIV therapiesî [tiab] OR ìHIV regimenî [tiab] OR ìHIV regimensî [tiab] OR ìHIV drug*î [tiab] OR ìHIV agent*î [tiab]

57. ìanti-HIV-1 treatment*î [tiab] OR ìanti-HIV-1 therapyî [tiab] OR ìanti-HIV-1 therapiesî [tiab] OR ìanti-HIV-1 regimenî [tiab] OR ìanti-HIV-1 regimensî [tiab] OR ìanti-HIV-1 drug*î [tiab] OR ìanti-HIV-1 agent*î [tiab]

58. ìHIV-1 treatment*î [tiab] OR ìHIV-1 therapyî [tiab] OR ìHIV-1 therapiesî [tiab] OR ìHIV-1 regimen*î [tiab] OR ìHIV-1 drug*î [tiab] OR ìHIV-1 agentî [tiab] OR ìHIV-1 agentsî [tiab]

59. ìanti-HIV-2 treatment*î [tiab] OR ìanti-HIV-2 therapyî [tiab] OR ìanti-HIV-2 therapiesî [tiab] OR ìanti-HIV-2 regimen*î [tiab] OR ìanti-HIV-2 drug*î [tiab] OR ìanti-HIV-2 agent*î [tiab] OR ìHIV-2 treatment*î [tiab] OR ìHIV-2 therapyî [tiab] OR ìHIV-2 therapiesî [tiab] OR HIV-2 regimen* [tiab] OR ìHIV-2 drug*î [tiab] OR HIV-2 agent* [tiab]

60. anti-AIDS treatment* [tiab] OR ìanti-AIDS therapyî [tiab] OR ìanti-AIDS therapiesî [tiab] OR anti-AIDS regimen* [tiab] OR ìanti-AIDS drug*î [tiab] OR ìanti-AIDS agent*î [tiab]

61. ìAIDS treatment*î [tiab] OR ìAIDS therapyî [tiab] OR ìAIDS therapiesî [tiab] OR ìAIDS regimen*î [tiab] OR ìAIDS drug*î [tiab] OR ìAIDS agent*î [tiab] OR HAART [tiab] OR HAART-exposed [tiab] OR HAART-treated [tiab] OR Mega-HAART [tiab]

62. ARV [tiab] OR ARVs [tiab] OR cARV [tiab] OR cARVs [tiab] OR ARV-exposed [tiab] OR ARV-treated [tiab] OR combination-ARV [tiab] OR combination-ARVs [tiab] OR combined-ARV [tiab] OR combined-ARVs [tiab]

63. ART [tiab] OR Multi-ART [tiab] OR Triple-ART [tiab] OR cART [tiab] OR ART-exposed [tiab] OR ART-treated [tiab] OR combination-ART [tiab] OR combined-ART [tiab] OR sc-ART [tiab]

64. ìshort-course-antiretroviral therapyî [tiab] OR ìshort-course-antiretroviral therapiesî [tiab] OR ìshort-course-anti-retroviral therapyî [tiab] OR ìshort-course-anti-retroviral therapiesî [tiab]

65. ìcombination treatment*î [tiab] OR ìcombination therapyî [tiab] OR ìcombination therapiesî [tiab] OR ìcombination regimen*î [tiab] OR ìcombination drug*î [tiab] OR ìcombination agent*î [tiab]

66. ìcombined treatment*î [tiab] OR ìcombined therapyî [tiab] OR ìcombined therapiesî [tiab] OR ìcombined regimen*î [tiab] OR ìcombined drug*î [tiab] OR ìcombined agent*î [tiab]

67. monotherapy [tiab] OR monotherapies [tiab] OR mono-therapy [tiab] OR mono-therapies [tiab] OR ìdual therapyî [tiab] OR ìdual therapiesî [tiab] OR ìdual drug therapyî [tiab] OR ìdual drug therapiesî [tiab] OR bitherap* [tiab]

68. PI [tiab] OR PIs [tiab] OR PI-based [tiab] OR PI-boosted [tiab] OR PI-containing [tiab] OR PI-therap* [tiab] OR PI-treatment* [tiab] OR PI-regimen* [tiab] OR Ritonavir-boosted [tiab] OR ìprotease inhibitor*î [tiab] OR NRTI [tiab] OR NRTIs [tiab] OR NRTI-based [tiab]

69. NRTI-containing [tiab] OR NRTI-therap* [tiab] OR NRTI-treatment* [tiab] OR NRTI-regimen* [tiab] OR ìnucleoside reverse transcriptase inhibitor*î [tiab] OR ìnucleoside analog reverse transcriptase inhibitor*î [tiab]

70. NNRTI [tiab] OR NNRTIs [tiab] OR NNRTI-based [tiab] OR NNRTI-containing [tiab] OR NNRTI-therap* [tiab] OR NNRTI-treatment* [tiab] OR NNRTI-regimen* [tiab]

71. ìnon nucleoside reverse transcriptase inhibitor*î [tiab] OR ìnon-nucleoside reverse transcriptase inhibitor*î [tiab] OR ìnonnucleoside reverse transcriptase inhibitor*î [tiab]
72. ìnon nucleoside analog reverse transcriptase inhibitor*î [tiab] OR ìnon-nucleoside analog reverse transcriptase inhibitor*î [tiab] OR ìnonnucleoside analog reverse transcriptase inhibitor*î [tiab]

73. NtRTI [tiab] OR NtRTIs [tiab] OR NtRTI-based [tiab] OR NtRTI-containing [tiab] OR NtRTI-therap* [tiab] OR NtRTI-treatment* [tiab] OR NtRTI-regimen* [tiab] OR ìnucleotide reverse transcriptase inhibitor*î [tiab] OR ìnucleotide analog reverse transcriptase inhibitor*î [tiab]

74. ìfusion inhibitor*î [tiab] OR ìCCR5 receptor antagonist*î [tiab] OR ìintegrase inhibitor*î [tiab] OR ìmaturation inhibitor*î [tiab] OR ìentry inhibitor*î [tiab]

75. Abacavir [tiab] OR ABC [tiab] OR Didanosine [tiab] OR ddI [tiab] OR Emtricitabine [tiab] OR FTC [tiab] OR Lamivudine [tiab] OR 3TC [tiab] OR Stavudine [tiab] OR d4T [tiab] OR Tenofovir [tiab] OR TFV [tiab] OR TDF [tiab]

76. Zidovudine [tiab] OR AZT [tiab] OR ZDV [tiab] OR Delavirdine [tiab] OR DLV [tiab] OR Efavirenz [tiab] OR EFV [tiab] OR Etravirine [tiab] OR ETR [tiab] OR Nevirapine [tiab] OR NVP [tiab] OR Rilpivirine [tiab] OR RPV [tiab]

77. Atazanavir [tiab] OR ATV [tiab] OR Atazanavir/Ritonavir [tiab] OR ATV/r [tiab] OR Darunavir [tiab] OR DRV [tiab] OR Darunavir/Ritonavir [tiab] OR DRV/r [tiab] OR Fosamprenavir [tiab] OR FPV [tiab] OR Fosamprenavir/Ritonavir [tiab] OR FPV/r [tiab]

78. Indinavir [tiab] OR IDV [tiab] OR Indinavir/Ritonavir [tiab] OR IDV/r [tiab] OR Lopinavir [tiab] OR LPV [tiab] OR Lopinavir/Ritonavir [tiab] OR LPV/r [tiab] OR Nelfinavir [tiab] OR NFV [tiab] OR Nelfinavir/Ritonavir [tiab] OR NFV/r [tiab]

79. Ritonavir [tiab] OR RTV [tiab] OR Saquinavir [tiab] OR SQV [tiab] OR Saquinavir/Ritonavir [tiab] OR SQV/r [tiab] OR Tipranavir [tiab] OR TPV [tiab] OR Tipranavir/Ritonavir [tiab] OR TPV/r [tiab] OR Enfuvirtide [tiab] OR T-20 [tiab]

80. Maraviroc [tiab] OR MVC [tiab] OR Rategravir [tiab] OR RAL [tiab] OR Elvitegravir [tiab] OR EVG [tiab] OR Zalcitabine [tiab] OR ddC [tiab] OR Combivir [tiab] OR Trizivir [tiab] OR Kaletra [tiab] OR Epzicom [tiab] OR Kivexa [tiab] OR Truvada [tiab] OR Atripla [tiab]

81. #50 or #51 or #52 or #53 or #54 or #55 or #56 or #57 or #58 or #59 or #60 or #61 or #62 or #63 or #64 or #65 or #66 or #67 or #68 or #69 or #70 or #71 or #72 or #73 or #74 or #75 or #76 or #77 or #78 or #79 or #80

82. #49 OR #81

83. #41 AND #82

84. 2018/04/29:2020/04/20 [dp]

85. #83 AND #84

Search strategy for "specific perinatal outcomes AND HIV OR antiretroviral therapy"
Database and platform: Pubmed (via https://www.ncbi.nlm.nih.gov/pubmed/)
Latest search date: 20 April 2020.

1. Premature Birth [Mesh] OR Fetal Membranes, Premature Rupture [Mesh] OR Obstetric Labor, Premature [Mesh] OR Infant, Extremely Premature [Mesh] OR Infant, Premature [Mesh]

2. prematurity [tiab] OR ìgestational age at birthî [tiab] OR ìgestational age at deliveryî [tiab] OR PTB [tiab] OR PTBs [tiab] OR VPTB [tiab] OR VPTBs [tiab]

3. pre-terms [tiab] OR preterms [tiab] OR pre-term birth* [tiab] OR ìpreterm birth*î [tiab] OR ìpremature birth*î [tiab] OR PTL [tiab] OR PTLs [tiab] OR VPTL [tiab] OR VPTLs [tiab]

4. ìpre-term labor*î [tiab] OR ìpre-term labour*î [tiab] OR ìpre-term obstetric labor*î [tiab] OR ìpre-term obstetric labour*î [tiab] OR ìpreterm labor*î [tiab] OR ìpreterm labour*î [tiab] OR preterm obstetric labor* [tiab] OR ìpreterm obstetric labour*î [tiab] OR ìpremature labor*î [tiab] OR ìpremature labour*î [tiab] OR premature obstetric labor* [tiab] OR ìpremature obstetric labour*î [tiab]

5. PTD [tiab] OR PTDs [tiab] OR VPTD [tiab] OR VPTDs [tiab] OR pre-term deliver* [tiab] OR preterm deliver* [tiab] OR ìpre-term infant*î [tiab] OR ìpreterm infant*î [tiab] OR premature deliver* [tiab] OR ìpremature infant*î [tiab] OR PROM [tiab] OR PPROM [tiab]

6. ìpreterm rupture of membranesî [tiab] OR preterm rupture of fetal membrane* [tiab] OR preterm rupture of foetal membrane* [tiab] OR pre-term rupture of membrane* [tiab] OR pre-term rupture of fetal membrane* [tiab] OR pre-term rupture of foetal membrane* [tiab] OR ìpremature rupture of membranesî [tiab] OR ìpremature rupture of fetal membranesî [tiab] OR ìpremature rupture of foetal membranesî [tiab]

7. #1 or #2 or #3 or #4 or #5 or #6

8. Fetal Growth Retardation [Mesh] OR Infant, Low Birth Weight [Mesh] OR Infant, Very Low Birth Weight [Mesh] OR Infant, Extremely Low Birth Weight [Mesh] OR Infant, Small for Gestational Age [Mesh]

9. IUGR [tiab] OR FGR [tiab] OR ìintrauterine growth restrictionî [tiab] OR ìintra-uterine growth restrictionî [tiab] OR ìintrauterine growth restrictedî [tiab] OR ìintra-uterine growth restrictedî [tiab] OR ìintrauterine growth retardationî [tiab] OR ìintra-uterine growth retardationî [tiab]

10. ìfetal growth restrictionî [tiab] OR ìfoetal growth restrictionî [tiab] OR ìfetal growth restrictedî [tiab] OR ìfoetal growth restrictedî [tiab] OR ìfetal growth retardationî [tiab] OR ìfoetal growth retardationî [tiab] OR SGA [tiab] OR SFGA [tiab] OR ìsmall for gestational ageî [tiab] OR ìsmall-for-gestational-ageî [tiab] OR ìsmall-for-gestational ageî [tiab] OR ìsmall for gestationî [tiab] OR ìsmall-for-gestationî [tiab]

11. VSGA [tiab] OR ìvery-small-for-gestational-ageî [tiab] OR ìvery-small-for-gestational ageî [tiab] OR SFD [tiab] OR ìsmall for datesî [tiab] OR ìsmall-for-datesî [tiab] OR ìweight for datesî [tiab] OR ìweight for gestational ageî [tiab] OR ìweight for age at deliveryî [tiab] OR ìweight at deliveryî [tiab]

12. ìbirthweight for datesî [tiab] OR ìbirthweight for gestational ageî [tiab] OR ìbirthweight for age at deliveryî [tiab] OR ìbirth weight for datesî [tiab] OR ìbirth weight for gestational ageî [tiab] OR ìbirth weight for age at deliveryî [tiab] OR ìbirth-weight for datesî [tiab] OR ìbirth-weight for gestational ageî [tiab] OR ìbirth-weight for age at deliveryî [tiab]

13. LBW [tiab] OR ìlow BWî [tiab] OR ìlow birth weightî [tiab] OR ìlow birth-weightî [tiab] OR ìlow-birth weightî [tiab] OR ìlow-birth-weightî [tiab] OR ìlow birthweightî [tiab] OR ìlow-birthweightî [tiab] OR ìlower BWî [tiab] OR ìlower birth weightî [tiab] OR ìlower birth-weightî [tiab] OR ìlower-birth weightî [tiab] OR ìlower-birth-weightî [tiab] OR ìlower birthweightî [tiab] OR ìlower-birthweightî [tiab]

14. ìreduced birth weightî [tiab] OR ìreduced birthweightî [tiab] OR ìreduced birth-weightî [tiab] OR VLBW [tiab] OR ìvery-low birthweightî [tiab] OR ìvery-low birth weightî [tiab] OR ìvery-low birth-weightî [tiab] OR ìvery-low-birthweightî [tiab] OR ìvery-low-birth-weightî [tiab] OR ELBW [tiab] OR ìextremely-low birthweightî [tiab] OR ìextremely-low birth weightî [tiab] OR ìextremely-low birth-weightî [tiab] OR ìextremely-low-birthweightî [tiab] OR ìextremely-low-birth-weightî [tiab]

15. #8 or #9 or #10 or #11 or #12 or #13 or #14

16. Stillbirth [Mesh] OR stillbirth* [tiab] OR still birth* [tiab] OR stillborn* [tiab] OR still born* [tiab] OR abortion* [tiab] OR miscarriage* [tiab] OR pregnancy death* [tiab] OR pregnancy loss* [tiab] OR pregnancy demise* [tiab] OR pregnancy mortalit* [tiab] OR gestational death* [tiab] OR gestational loss* [tiab] OR gestational demise* [tiab] OR "gestational mortalit*" [tiab]

17. Fetal Death [Mesh] OR "fetal death*" [tiab] OR "fetal loss*" [tiab] OR fetal demise* [tiab] OR fetal mortalit* [tiab] OR "foetal death*" [tiab] OR "foetal loss*" [tiab] OR foetal demise* [tiab] OR foetal mortalit* [tiab] OR "obstetric death*" [tiab] OR obstetric loss* [tiab] OR obstetric demise* [tiab] OR obstetric mortalit* [tiab] OR obstetrical death* [tiab] OR obstetrical loss* [tiab] OR obstetrical demise* [tiab] OR obstetrical mortalit* [tiab]

18. "labor death*" [tiab] OR labor loss* [tiab] OR labor demise* [tiab] OR labor mortalit* [tiab] OR labour death* [tiab] OR labour loss* [tiab] OR labour demise* [tiab] OR labour mortalit* [tiab] OR birth death* [tiab] OR birth loss* [tiab] OR birth demise* [tiab] OR birth mortalit* [tiab]

19. delivery death* [tiab] OR delivery loss* [tiab] OR delivery demise* [tiab] OR delivery mortalit* [tiab] OR neonate death* [tiab] OR neonate loss* [tiab] OR neonate demise* [tiab] OR neonate mortalit* [tiab] OR "newborn death*" [tiab] OR "newborn loss*" [tiab] OR "newborn demise*" [tiab] OR newborn mortalit* [tiab]

20. new-born death* [tiab] OR new-born loss* [tiab] OR new-born demise* [tiab] OR new-born mortalit* [tiab] OR "new born death*" [tiab] OR "new born loss*" [tiab] OR "new born demise*" [tiab] OR "new born mortalit*" [tiab] OR "infant death*" [tiab] OR infant loss* [tiab] OR infant demise* [tiab] OR infant mortalit* [tiab] OR reproductive death* [tiab] OR "reproductive loss*" [tiab] OR reproductive demise* [tiab] OR reproductive mortalit* [tiab]

21. prelabour death* [tiab] OR prelabour loss* [tiab] OR prelabour demise* [tiab] OR prelabour mortalit* [tiab] OR prelabor death* [tiab] OR prelabor loss* [tiab] OR prelabor

demise* [tiab] OR prelabor mortalit* [tiab] OR pre-labour death* [tiab] OR pre-labour loss* [tiab] OR pre-labour demise* [tiab] OR pre-labour mortalit* [tiab] OR pre-labor death* [tiab] OR pre-labor loss* [tiab] OR pre-labor demise* [tiab] OR pre-labor mortalit* [tiab]

22. "intrauterine death*" [tiab] OR intrauterine loss* [tiab] OR intrauterine demise* [tiab] OR intrauterine mortalit* [tiab] OR intra-uterine death* [tiab] OR intra-uterine loss* [tiab] OR intra-uterine demise* [tiab] OR intra-uterine mortalit* [tiab] OR antenatal death* [tiab] OR antenatal loss* [tiab] OR antenatal demise* [tiab] OR antenatal mortalit* [tiab] OR ante-natal death* [tiab] OR ante-natal loss* [tiab] OR ante-natal demise* [tiab] OR ante-natal mortalit* [tiab]

23. prenatal death* [tiab] OR prenatal loss* [tiab] OR prenatal demise* [tiab] OR prenatal mortalit* [tiab] OR pre-natal death* [tiab] OR pre-natal loss* [tiab] OR pre-natal demise* [tiab] OR pre-natal mortalit* [tiab] OR "perinatal death" [tiab] OR "perinatal deaths" [tiab] OR perinatal loss* [tiab] OR perinatal demise* [tiab] OR "perinatal mortality" [tiab] OR "perinatal mortalities" [tiab] OR peri-natal death* [tiab] OR peri-natal loss* [tiab] OR peri-natal demise* [tiab] OR peri-natal mortalit* [tiab]

24. "neonatal death" [tiab] OR "neonatal deaths" [tiab] OR neonatal loss* [tiab] OR neonatal demise* [tiab] OR "neonatal mortality" [tiab] OR "neonatal mortalities" [tiab] OR neo-natal death* [tiab] OR neo-natal loss* [tiab] OR neo-natal demise* [tiab] OR neo-natal mortalit* [tiab] OR postnatal death* [tiab] OR postnatal loss* [tiab] OR postnatal demise* [tiab] OR postnatal mortalit* [tiab] OR post-natal death* [tiab] OR post-natal loss* [tiab] OR post-natal demise* [tiab] OR post-natal mortalit* [tiab]

25. antepartum death* [tiab] OR antepartum loss* [tiab] OR antepartum demise* [tiab] OR antepartum mortalit* [tiab] OR ante-partum death* [tiab] OR ante-partum loss* [tiab] OR ante-partum demise* [tiab] OR ante-partum mortalit* [tiab] OR intrapartum death* [tiab] OR intrapartum loss* [tiab] OR intrapartum demise* [tiab] OR intrapartum mortalit* [tiab] OR intra-partum death* [tiab] OR intra-partum loss* [tiab] OR intra-partum demise* [tiab] OR intra-partum mortalit* [tiab]

26. peripartum death* [tiab] OR peripartum loss* [tiab] OR peripartum demise* [tiab] OR peripartum mortalit* [tiab] OR peri-partum death* [tiab] OR peri-partum loss* [tiab] OR peri-partum demise* [tiab] OR peri-partum mortalit* [tiab] OR postpartum death* [tiab] OR postpartum loss* [tiab] OR postpartum demise* [tiab] OR postpartum mortalit* [tiab] OR post-partum death* [tiab] OR post-partum loss* [tiab] OR post-partum demise* [tiab] OR post-partum mortalit* [tiab]

27. #16 or #17 or #18 or #19 or #20 or #21 or #22 or #23 or #24 or #25 or #26

28. HIV [Mesh] OR HIV Seropositivity [Mesh] OR HIV Infections [Mesh] OR HIV-2 [Mesh] OR HIV-1 [Mesh] OR AIDS Serodiagnosis [Mesh] OR Acquired Immunodeficiency Syndrome [Mesh] OR AIDS Arteritis, Central Nervous System [Mesh] OR AIDS-Associated Nephropathy [Mesh] OR AIDS Dementia Complex [Mesh] OR AIDS-Related Opportunistic Infections [Mesh] OR Lymphoma, AIDS-Related [Mesh]

29. HIV [tiab] OR HIV-1 [tiab] OR HIV-type-1 [tiab] OR ìHTLV IIIî [tiab] OR HTLV-III [tiab] OR ìHTLV type IIIî [tiab] OR HTLV-type-III [tiab] OR LAV [tiab] OR HTLV-III-LAV [tiab] OR LAV-HTLV-III [tiab] OR HIV-2 [tiab] OR HIV-type-2 [tiab] OR HIV-II [tiab] OR HTLV-IV [tiab] OR LAV-2 [tiab]

30. HIV-positive [tiab] OR HIV-1-positive [tiab] OR HIV-2-positive [tiab] OR HIV-infected [tiab] OR HIV-1-infected [tiab] OR HIV-type-1-infected [tiab] OR ìHTLV III-infectedî [tiab] OR HTLV-III-infected [tiab] OR ìHTLV type III-infectedî [tiab] OR HTLV-type-III-infected [tiab] OR LAV-infected [tiab]

31. HTLV-III-LAV-infected [tiab] OR LAV-HTLV-III-infected [tiab] OR HIV-2-infected [tiab] OR HIV-type-2-infected [tiab] OR HIV-II-infected [tiab] OR HTLV-IV-infected [tiab] OR LAV-2-infected [tiab] OR HIV-infection* [tiab] OR HIV-1-infection* [tiab] OR HIV-type-1-infection* [tiab] OR ìHTLV III-infection*î [tiab] OR HTLV-III-infection* [tiab] OR HTLV type III-infection* [tiab] OR HTLV-type-III-infection* [tiab]

32. LAV-infection* [tiab] OR HTLV-III-LAV-infection* [tiab] OR LAV-HTLV-III-infection* [tiab] OR HIV-2-infection* [tiab] OR HIV-type-2-infection* [tiab] OR HIV-II-infection* [tiab] OR HTLV-IV-infection* [tiab] OR LAV-2-infection* [tiab] OR Human Immunodeficiency Virus* [tiab] OR ìHuman Immune Deficiency Virus*î [tiab]

33. ìHuman T Cell Lymphotropic Virus Type IIIî [tiab] OR ìHuman T-Cell Lymphotropic Virus Type IIIî [tiab] OR ìHuman T Lymphotropic Virus Type IIIî [tiab] OR ìHuman T-Lymphotropic Virus Type IIIî [tiab] OR ìHuman T Lymphotropic Virus Type IVî [tiab] OR ìHuman T-Lymphotropic Virus Type IVî [tiab] OR ìHuman T Cell Leukemia Virus Type IIIî [tiab] OR ìHuman T-Cell Leukemia Virus Type IIIî [tiab]

34. ìLymphadenopathy-Associated Virus*î [tiab] OR ìLymphadenopathy Associated Virus*î [tiab] OR AIDS [tiab] OR ìAcquired Immune Deficiency Syndromeî [tiab] OR ìAcquired Immunodeficiency Syndromeî [tiab]

35. #28 or #29 or #30 or #31 or #32 or #33 or #34

36. Anti-HIV Agents [Mesh] OR HIV Fusion Inhibitors [Mesh] OR HIV Integrase Inhibitors [Mesh] OR HIV Protease Inhibitors [Mesh]

37. antiretrovirals [tiab] OR ìantiretroviral treatment*î [tiab] OR ìantiretroviral therapyî [tiab] OR ìantiretroviral therapiesî [tiab] OR ìantiretroviral regimen*î [tiab] OR ìantiretroviral drug*î [tiab] OR ìantiretroviral agent*î [tiab]

38. anti-retrovirals [tiab] OR ìanti-retroviral treatment*î [tiab] OR ìanti-retroviral therapyî [tiab] OR ìanti-retroviral therapiesî [tiab] OR ìanti-retroviral regimen*î [tiab] OR ìanti-retroviral drug*î [tiab] OR ìanti-retroviral agent*î [tiab]

39. antivirals [tiab] OR ìantiviral treatment*î [tiab] OR ìantiviral therapyî [tiab] OR ìantiviral therapiesî [tiab] OR ìantiviral regimen*î [tiab] OR ìantiviral drug*î [tiab] OR ìantiviral agent*î [tiab]

40. anti-virals [tiab] OR ìanti-viral treatment*î [tiab] OR ìanti-viral therapyî [tiab] OR ìanti-viral therapiesî [tiab] OR ìanti-viral regimen*î [tiab] OR ìanti-viral drug*î [tiab] OR ìanti-viral agent*î [tiab]

41. ìanti-HIV treatment*î [tiab] OR ìanti-HIV therapyî [tiab] OR ìanti-HIV therapiesî [tiab] OR ìanti-HIV regimen*î [tiab] OR ìanti-HIV drug*î [tiab] OR ìanti-HIV agent*î [tiab]

42. ìHIV treatment*î [tiab] OR ìHIV therapyî [tiab] OR ìHIV therapiesî [tiab] OR ìHIV regimenî [tiab] OR ìHIV regimensî [tiab] OR ìHIV drug*î [tiab] OR ìHIV agent*î [tiab]

43. ìanti-HIV-1 treatment*î [tiab] OR ìanti-HIV-1 therapyî [tiab] OR ìanti-HIV-1 therapiesî [tiab] OR ìanti-HIV-1 regimenî [tiab] OR ìanti-HIV-1 regimensî [tiab] OR ìanti-HIV-1 drug*î [tiab] OR ìanti-HIV-1 agent*î [tiab]

44. ìHIV-1 treatment*î [tiab] OR ìHIV-1 therapyî [tiab] OR ìHIV-1 therapiesî [tiab] OR ìHIV-1 regimen*î [tiab] OR ìHIV-

1 drug*î [tiab] OR ìHIV-1 agentî [tiab] OR ìHIV-1 agentsî [tiab]

45. ìanti-HIV-2 treatment*î [tiab] OR ìanti-HIV-2 therapyî [tiab] OR ìanti-HIV-2 therapiesî [tiab] OR ìanti-HIV-2 regimen*î [tiab] OR ìanti-HIV-2 drug*î [tiab] OR ìanti-HIV-2 agent*î [tiab] OR ìHIV-2 treatment*î [tiab] OR ìHIV-2 therapyî [tiab] OR ìHIV-2 therapiesî [tiab] OR HIV-2 regimen* [tiab] OR ìHIV-2 drug*î [tiab] OR HIV-2 agent* [tiab]

46. anti-AIDS treatment* [tiab] OR ìanti-AIDS therapyî [tiab] OR ìanti-AIDS therapiesî [tiab] OR anti-AIDS regimen* [tiab] OR ìanti-AIDS drug*î [tiab] OR ìanti-AIDS agent*î [tiab]

47. ìAIDS treatment*î [tiab] OR ìAIDS therapyî [tiab] OR ìAIDS therapiesî [tiab] OR ìAIDS regimen*î [tiab] OR ìAIDS drug*î [tiab] OR ìAIDS agent*î [tiab] OR HAART [tiab] OR HAART-exposed [tiab] OR HAART-treated [tiab] OR Mega-HAART [tiab]

48. ARV [tiab] OR ARVs [tiab] OR cARV [tiab] OR cARVs [tiab] OR ARV-exposed [tiab] OR ARV-treated [tiab] OR combination-ARV [tiab] OR combination-ARVs [tiab] OR combined-ARV [tiab] OR combined-ARVs [tiab]

49. ART [tiab] OR Multi-ART [tiab] OR Triple-ART [tiab] OR cART [tiab] OR ART-exposed [tiab] OR ART-treated [tiab] OR combination-ART [tiab] OR combined-ART [tiab] OR sc-ART [tiab]

50. ìshort-course-antiretroviral therapyî [tiab] OR ìshort-course-antiretroviral therapiesî [tiab] OR ìshort-course-anti-retroviral therapyî [tiab] OR ìshort-course-anti-retroviral therapiesî [tiab]

51. ìcombination treatment*î [tiab] OR ìcombination therapyî [tiab] OR ìcombination therapiesî [tiab] OR ìcombination regimen*î [tiab] OR ìcombination drug*î [tiab] OR ìcombination agent*î [tiab]

52. ìcombined treatment*î [tiab] OR ìcombined therapyî [tiab] OR ìcombined therapiesî [tiab] OR ìcombined regimen*î [tiab] OR ìcombined drug*î [tiab] OR ìcombined agent*î [tiab]

53. monotherapy [tiab] OR monotherapies [tiab] OR mono-therapy [tiab] OR mono-therapies [tiab] OR ìdual therapyî [tiab] OR ìdual therapiesî [tiab] OR ìdual drug therapyî [tiab] OR ìdual drug therapiesî [tiab] OR bitherap* [tiab]

54. PI [tiab] OR PIs [tiab] OR PI-based [tiab] OR PI-boosted [tiab] OR PI-containing [tiab] OR PI-therap* [tiab] OR PI-treatment* [tiab] OR PI-regimen* [tiab] OR Ritonavir-boosted [tiab] OR ìprotease inhibitor*î [tiab] OR NRTI [tiab] OR NRTIs [tiab] OR NRTI-based [tiab]

55. NRTI-containing [tiab] OR NRTI-therap* [tiab] OR NRTI-treatment* [tiab] OR NRTI-regimen* [tiab] OR ìnucleoside reverse transcriptase inhibitor*î [tiab] OR ìnucleoside analog reverse transcriptase inhibitor*î [tiab]

56. NNRTI [tiab] OR NNRTIs [tiab] OR NNRTI-based [tiab] OR NNRTI-containing [tiab] OR NNRTI-therap* [tiab] OR NNRTI-treatment* [tiab] OR NNRTI-regimen* [tiab]

57. ìnon nucleoside reverse transcriptase inhibitor*î [tiab] OR ìnon-nucleoside reverse transcriptase inhibitor*î [tiab] OR ìnonnucleoside reverse transcriptase inhibitor*î [tiab]

58. ìnon nucleoside analog reverse transcriptase inhibitor*î [tiab] OR ìnon-nucleoside analog reverse transcriptase inhibitor*î [tiab] OR ìnonnucleoside analog reverse transcriptase inhibitor*î [tiab]

59. NtRTI [tiab] OR NtRTIs [tiab] OR NtRTI-based [tiab] OR NtRTI-containing [tiab] OR NtRTI-therap* [tiab] OR NtRTI-treatment* [tiab] OR NtRTI-regimen* [tiab] OR ìnucleotide reverse transcriptase inhibitor*î [tiab] OR ìnucleotide analog reverse transcriptase inhibitor*î [tiab]

60. ìfusion inhibitor*î [tiab] OR ìCCR5 receptor antagonist*î [tiab] OR ìintegrase inhibitor*î [tiab] OR ìmaturation inhibitor*î [tiab] OR ìentry inhibitor*î [tiab]

61. Abacavir [tiab] OR ABC [tiab] OR Didanosine [tiab] OR ddI [tiab] OR Emtricitabine [tiab] OR FTC [tiab] OR Lamivudine [tiab] OR 3TC [tiab] OR Stavudine [tiab] OR d4T [tiab] OR Tenofovir [tiab] OR TFV [tiab] OR TDF [tiab]

62. Zidovudine [tiab] OR AZT [tiab] OR ZDV [tiab] OR Delavirdine [tiab] OR DLV [tiab] OR Efavirenz [tiab] OR EFV [tiab] OR Etravirine [tiab] OR ETR [tiab] OR Nevirapine [tiab] OR NVP [tiab] OR Rilpivirine [tiab] OR RPV [tiab]

63. Atazanavir [tiab] OR ATV [tiab] OR Atazanavir/Ritonavir [tiab] OR ATV/r [tiab] OR Darunavir [tiab] OR DRV [tiab] OR Darunavir/Ritonavir [tiab] OR DRV/r [tiab] OR Fosamprenavir [tiab] OR FPV [tiab] OR Fosamprenavir/Ritonavir [tiab] OR FPV/r [tiab]

64. Indinavir [tiab] OR IDV [tiab] OR Indinavir/Ritonavir [tiab] OR IDV/r [tiab] OR Lopinavir [tiab] OR LPV [tiab] OR Lopinavir/Ritonavir [tiab] OR LPV/r [tiab] OR Nelfinavir [tiab] OR NFV [tiab] OR Nelfinavir/Ritonavir [tiab] OR NFV/r [tiab]

65. Ritonavir [tiab] OR RTV [tiab] OR Saquinavir [tiab] OR SQV [tiab] OR Saquinavir/Ritonavir [tiab] OR SQV/r [tiab] OR Tipranavir [tiab] OR TPV [tiab] OR Tipranavir/Ritonavir [tiab] OR TPV/r [tiab] OR Enfuvirtide [tiab] OR T-20 [tiab]

66. Maraviroc [tiab] OR MVC [tiab] OR Raltegravir [tiab] OR RAL [tiab] OR Elvitegravir [tiab] OR EVG [tiab] OR Zalcitabine [tiab] OR ddC [tiab] OR Combivir [tiab] OR Trizivir [tiab] OR Kaletra [tiab] OR Epzicom [tiab] OR Kivexa [tiab] OR Truvada [tiab] OR Atripla [tiab]

67. #36 or #37 or #38 or #39 or #40 or #41 or #42 or #43 or #44 or #45 or #46 or #47 or #48 or #49 or #50 or #51 or #52 or #53 or #54 or #55 or #56 or #57 or #58 or #59 or #60 or #61 or #62 or #63 or #64 or #65 or #66

68. #7 OR #15 OR #27

69. #35 OR #67

70. #68 AND #69

71. 2018/04/29:2020/04/20 [dp]

72. #70 AND #71

Search strategy for "pregnancy outcome OR specific perinatal outcomes AND new antiretroviral therapy"
Database and platform: Pubmed (via https://www.ncbi.nlm.nih.gov/pubmed/)
Latest search date: 20 April 2020.

1. Pregnancy Outcome [Mesh] OR Pregnancy Complications, Infectious [Mesh] OR "pregnancy outcome*" [tiab] OR "pregnancy complication*" [tiab] OR pregnancy consequence* [tiab] OR "pregnancy characteristic" [tiab] OR "pregnancy characteristics" [tiab] OR pregnancy event* [tiab] OR pregnancy result* [tiab] OR pregnancy problem* [tiab] OR pregnancy morbidit* [tiab] OR pregnancy sequelae [tiab]

2. gestational outcome* [tiab] OR gestational complication* [tiab] OR gestational consequence* [tiab] OR gestational characteristic* [tiab] OR gestational event* [tiab] OR gestational result* [tiab] OR gestational problem* [tiab] OR gestational morbidit* [tiab] OR gestational sequelae [tiab]

3. "fetal outcome" [tiab] OR "fetal outcomes" [tiab] OR "fetal complication" [tiab] OR "fetal complications" [tiab] OR fetal consequence* [tiab] OR fetal characteristic* [tiab] OR fetal event* [tiab] OR fetal result* [tiab] OR fetal problem* [tiab] OR fetal morbidit* [tiab] OR fetal sequelae [tiab]

4. foetal outcome* [tiab] OR foetal complication* [tiab] OR foetal consequence* [tiab] OR foetal characteristic* [tiab] OR foetal event* [tiab] OR foetal result* [tiab] OR foetal problem* [tiab] OR foetal morbidit* [tiab] OR foetal sequelae [tiab]

5. "obstetric outcome" [tiab] OR "obstetric outcomes" [tiab] OR "obstetric complication" [tiab] OR "obstetric complications" [tiab] OR obstetric consequence* [tiab] OR obstetric characteristic* [tiab] OR obstetric event* [tiab] OR obstetric result* [tiab] OR "obstetric problem" [tiab] OR "obstetric problems" [tiab] OR obstetric morbidit* [tiab] OR obstetric sequelae [tiab]

6. obstetrical outcome* [tiab] OR "obstetrical complication" [tiab] OR "obstetrical complications" [tiab] OR obstetrical consequence* [tiab] OR obstetrical characteristic* [tiab] OR obstetrical event* [tiab] OR obstetrical result* [tiab] OR "obstetrical problem" [tiab] OR "obstetrical problems" [tiab] OR obstetrical morbidit* [tiab] OR obstetrical sequelae [tiab]

7. "labor outcome" [tiab] OR "labor outcomes" [tiab] OR "labor complication" [tiab] OR "labor complications" [tiab] OR "labor consequence" [tiab] OR "labor consequences" [tiab] OR labor characteristic* [tiab] OR labor event* [tiab] OR labor result* [tiab] OR labor problem* [tiab] OR labor morbidit* [tiab] OR labor sequelae [tiab]

8. labour outcome* [tiab] OR labour complication* [tiab] OR "labour consequence" [tiab] OR "labour consequences" [tiab] OR labour characteristic* [tiab] OR labour event* [tiab] OR labour result* [tiab] OR labour problem* [tiab] OR labour morbidit* [tiab] OR labour sequelae [tiab]

9. "birth outcome" [tiab] OR "birth outcomes" [tiab] OR birth complication* [tiab] OR birth consequence* [tiab] OR birth characteristic* [tiab] OR birth event* [tiab] OR birth result* [tiab] OR birth problem* [tiab] OR birth morbidit* [tiab] OR birth sequelae [tiab]

10. delivery outcome* [tiab] OR delivery complication* [tiab] OR delivery consequence* [tiab] OR delivery characteristic* [tiab] OR delivery event* [tiab] OR delivery result* [tiab] OR delivery problem* [tiab] OR delivery morbidit* [tiab] OR delivery sequelae [tiab]

11. neonate outcome* [tiab] OR neonate complication* [tiab] OR neonate consequence* [tiab] OR neonate characteristic* [tiab] OR neonate event* [tiab] OR neonate result* [tiab] OR neonate problem* [tiab] OR neonate morbidit* [tiab] OR neonate sequelae [tiab]

12. newborn outcome* [tiab] OR newborn complication* [tiab] OR newborn consequence* [tiab] OR newborn characteristic* [tiab] OR newborn event* [tiab] OR newborn result* [tiab] OR newborn problem* [tiab] OR newborn morbidit* [tiab] OR newborn sequelae [tiab]

13. new-born outcome* [tiab] OR new-born complication* [tiab] OR new-born consequence* [tiab] OR new-born characteristic* [tiab] OR new-born event* [tiab] OR new-born result* [tiab] OR new-born problem* [tiab] OR new-born morbidit* [tiab] OR new-born sequelae [tiab]

14. new born outcome* [tiab] OR new born complication* [tiab] OR new born consequence* [tiab] OR new born characteristic* [tiab] OR new born event* [tiab] OR new born result* [tiab] OR new born problem* [tiab] OR new born morbidit* [tiab] OR new born sequelae [tiab]

15. infant outcome* [tiab] OR infant complication* [tiab] OR infant consequence* [tiab] OR infant characteristic* [tiab] OR infant event* [tiab] OR infant result* [tiab] OR infant problem* [tiab] OR infant morbidit* [tiab] OR infant sequelae [tiab]

16. reproductive outcome* [tiab] OR reproductive complication* [tiab] OR reproductive consequence* [tiab] OR reproductive characteristic* [tiab] OR reproductive event* [tiab] OR reproductive result* [tiab] OR reproductive problem* [tiab] OR reproductive morbidit* [tiab] OR reproductive sequelae [tiab]

17. prelabour outcome* [tiab] OR prelabour complication* [tiab] OR prelabour consequence* [tiab] OR prelabour characteristic* [tiab] OR prelabour event* [tiab] OR prelabour result* [tiab] OR prelabour problem* [tiab] OR prelabour morbidit* [tiab] OR prelabour sequelae [tiab]

18. prelabor outcome* [tiab] OR prelabor complication* [tiab] OR prelabor consequence* [tiab] OR prelabor characteristic* [tiab] OR prelabor event* [tiab] OR prelabor result* [tiab] OR prelabor problem* [tiab] OR prelabor morbidit* [tiab] OR prelabor sequelae [tiab]

19. pre-labour outcome* [tiab] OR pre-labour complication* [tiab] OR pre-labour consequence* [tiab] OR pre-labour characteristic* [tiab] OR pre-labour event* [tiab] OR pre-labour result* [tiab] OR pre-labour problem* [tiab] OR pre-labour morbidit* [tiab] OR pre-labour sequelae [tiab]

20. pre-labor outcome* [tiab] OR pre-labor complication* [tiab] OR pre-labor consequence* [tiab] OR pre-labor characteristic* [tiab] OR pre-labor event* [tiab] OR pre-labor result* [tiab] OR pre-labor problem* [tiab] OR pre-labor morbidit* [tiab] OR pre-labor sequelae [tiab]

21. intrauterine outcome* [tiab] OR intrauterine complication* [tiab] OR intrauterine consequence* [tiab] OR intrauterine

characteristic* [tiab] OR intrauterine event* [tiab] OR intrauterine result* [tiab] OR intrauterine problem* [tiab] OR intrauterine morbidit* [tiab] OR intrauterine sequelae [tiab]

22. intra-uterine outcome* [tiab] OR intra-uterine complication* [tiab] OR intra-uterine consequence* [tiab] OR intra-uterine characteristic* [tiab] OR intra-uterine event* [tiab] OR intra-uterine result* [tiab] OR intra-uterine problem* [tiab] OR intra-uterine morbidit* [tiab] OR intra-uterine sequelae [tiab]

23. antenatal outcome* [tiab] OR antenatal complication* [tiab] OR antenatal consequence* [tiab] OR antenatal characteristic* [tiab] OR antenatal event* [tiab] OR antenatal result* [tiab] OR antenatal problem* [tiab] OR antenatal morbidit* [tiab] OR antenatal sequelae [tiab]

24. ante-natal outcome* [tiab] OR ante-natal complication* [tiab] OR ante-natal consequence* [tiab] OR ante-natal characteristic* [tiab] OR ante-natal event* [tiab] OR ante-natal result* [tiab] OR ante-natal problem* [tiab] OR ante-natal morbidit* [tiab] OR ante-natal sequelae [tiab]

25. prenatal outcome* [tiab] OR prenatal complication* [tiab] OR prenatal consequence* [tiab] OR prenatal characteristic* [tiab] OR prenatal event* [tiab] OR prenatal result* [tiab] OR prenatal problem* [tiab] OR prenatal morbidit* [tiab] OR prenatal sequelae [tiab]

26. pre-natal outcome* [tiab] OR pre-natal complication* [tiab] OR pre-natal consequence* [tiab] OR pre-natal characteristic* [tiab] OR pre-natal event* [tiab] OR pre-natal result* [tiab] OR pre-natal problem* [tiab] OR pre-natal morbidit* [tiab] OR pre-natal sequelae [tiab]

27. perinatal outcome* [tiab] OR perinatal complication* [tiab] OR perinatal consequence* [tiab] OR perinatal characteristic* [tiab] OR perinatal event* [tiab] OR perinatal result* [tiab] OR perinatal problem* [tiab] OR perinatal morbidit* [tiab] OR perinatal sequelae [tiab]

28. peri-natal outcome* [tiab] OR peri-natal complication* [tiab] OR peri-natal consequence* [tiab] OR peri-natal characteristic* [tiab] OR peri-natal event* [tiab] OR peri-natal result* [tiab] OR peri-natal problem* [tiab] OR peri-natal morbidit* [tiab] OR peri-natal sequelae [tiab]
29. neonatal outcome* [tiab] OR neonatal complication* [tiab] OR neonatal consequence* [tiab] OR neonatal characteristic* [tiab] OR neonatal event* [tiab] OR neonatal result* [tiab] OR neonatal problem* [tiab] OR neonatal morbidit* [tiab] OR neonatal sequelae [tiab]

30. neo-natal outcome* [tiab] OR neo-natal complication* [tiab] OR neo-natal consequence* [tiab] OR neo-natal characteristic* [tiab] OR neo-natal event* [tiab] OR neo-natal result* [tiab] OR neo-natal problem* [tiab] OR neo-natal morbidit* [tiab] OR neo-natal sequelae [tiab]

31. postnatal outcome* [tiab] OR postnatal complication* [tiab] OR postnatal consequence* [tiab] OR postnatal characteristic* [tiab] OR postnatal event* [tiab] OR postnatal result* [tiab] OR postnatal problem* [tiab] OR postnatal morbidit* [tiab] OR postnatal sequelae [tiab]

32. post-natal outcome* [tiab] OR post-natal complication* [tiab] OR post-natal consequence* [tiab] OR post-natal characteristic* [tiab] OR post-natal event* [tiab] OR post-natal result* [tiab] OR post-natal problem* [tiab] OR post-natal morbidit* [tiab] OR post-natal sequelae [tiab]

33. antepartum outcome* [tiab] OR antepartum complication* [tiab] OR antepartum consequence* [tiab] OR antepartum characteristic* [tiab] OR antepartum event* [tiab] OR antepartum result* [tiab] OR antepartum problem* [tiab] OR antepartum morbidit* [tiab] OR antepartum sequelae [tiab]

34. ante-partum outcome* [tiab] OR ante-partum complication* [tiab] OR ante-partum consequence* [tiab] OR ante-partum characteristic* [tiab] OR ante-partum event* [tiab] OR ante-partum result* [tiab] OR ante-partum problem* [tiab] OR ante-partum morbidit* [tiab] OR ante-partum sequelae [tiab]

35. intrapartum outcome* [tiab] OR intrapartum complication* [tiab] OR intrapartum consequence* [tiab] OR intrapartum characteristic* [tiab] OR intrapartum event* [tiab] OR intrapartum result* [tiab] OR intrapartum problem* [tiab] OR intrapartum morbidit* [tiab] OR intrapartum sequelae [tiab]

36. intra-partum outcome* [tiab] OR intra-partum complication* [tiab] OR intra-partum consequence* [tiab] OR intra-partum characteristic* [tiab] OR intra-partum event* [tiab] OR intra-partum result* [tiab] OR intra-partum problem* [tiab] OR intra-partum morbidit* [tiab] OR intra-partum sequelae [tiab]

37. peripartum outcome* [tiab] OR peripartum complication* [tiab] OR peripartum consequence* [tiab] OR peripartum characteristic* [tiab] OR peripartum event* [tiab] OR peripartum result* [tiab] OR peripartum problem* [tiab] OR peripartum morbidit* [tiab] OR peripartum sequelae [tiab]

38. peri-partum outcome* [tiab] OR peri-partum complication* [tiab] OR peri-partum consequence* [tiab] OR peri-partum characteristic* [tiab] OR peri-partum event* [tiab] OR peri-partum result* [tiab] OR peri-partum problem* [tiab] OR peri-partum morbidit* [tiab] OR peri-partum sequelae [tiab]
39. postpartum outcome* [tiab] OR postpartum complication* [tiab] OR postpartum consequence* [tiab] OR postpartum characteristic* [tiab] OR postpartum event* [tiab] OR postpartum result* [tiab] OR postpartum problem* [tiab] OR postpartum morbidit* [tiab] OR postpartum sequelae [tiab]

40. post-partum outcome* [tiab] OR post-partum complication* [tiab] OR post-partum consequence* [tiab] OR post-partum characteristic* [tiab] OR post-partum event* [tiab] OR post-partum result* [tiab] OR post-partum problem* [tiab] OR post-partum morbidit* [tiab] OR post-partum sequelae [tiab]

41. Premature Birth [Mesh] OR Fetal Membranes, Premature Rupture [Mesh] OR Obstetric Labor, Premature [Mesh] OR Infant, Extremely Premature [Mesh] OR Infant, Premature [Mesh]

42. prematurity [tiab] OR ìgestational age at birthî [tiab] OR ìgestational age at deliveryî [tiab] OR PTB [tiab] OR PTBs [tiab] OR VPTB [tiab] OR VPTBs [tiab]

43. pre-terms [tiab] OR preterms [tiab] OR pre-term birth* [tiab] OR ìpreterm birth*î [tiab] OR ìpremature birth*î [tiab] OR PTL [tiab] OR PTLs [tiab] OR VPTL [tiab] OR VPTLs [tiab]

44. ìpre-term labor*î [tiab] OR ìpre-term labour*î [tiab] OR ìpre-term obstetric labor*î [tiab] OR ìpre-term obstetric labour*î [tiab] OR ìpreterm labor*î [tiab] OR ìpreterm labour*î [tiab] OR preterm obstetric labor* [tiab] OR ìpreterm obstetric labour*î [tiab] OR ìpremature labor*î [tiab] OR ìpremature

labour*î [tiab] OR premature obstetric labor* [tiab] OR ìpremature obstetric labour*î [tiab]

45. PTD [tiab] OR PTDs [tiab] OR VPTD [tiab] OR VPTDs [tiab] OR pre-term deliver* [tiab] OR preterm deliver* [tiab] OR ìpre-term infant*î [tiab] OR ìpreterm infant*î [tiab] OR premature deliver* [tiab] OR ìpremature infant*î [tiab] OR PROM [tiab] OR PPROM [tiab]

46. ìpreterm rupture of membranesî [tiab] OR preterm rupture of fetal membrane* [tiab] OR preterm rupture of foetal membrane* [tiab] OR pre-term rupture of membrane* [tiab] OR pre-term rupture of fetal membrane* [tiab] OR pre-term rupture of foetal membrane* [tiab] OR ìpremature rupture of membranesî [tiab] OR ìpremature rupture of fetal membranesî [tiab] OR ìpremature rupture of foetal membranesî [tiab]

47. Fetal Growth Retardation [Mesh] OR Infant, Low Birth Weight [Mesh] OR Infant, Very Low Birth Weight [Mesh] OR Infant, Extremely Low Birth Weight [Mesh] OR Infant, Small for Gestational Age [Mesh]

48. IUGR [tiab] OR FGR [tiab] OR ìintrauterine growth restrictionî [tiab] OR ìintra-uterine growth restrictionî [tiab] OR ìintrauterine growth restrictedî [tiab] OR ìintra-uterine growth restrictedî [tiab] OR ìintrauterine growth retardationî [tiab] OR ìintra-uterine growth retardationî [tiab]

49. ìfetal growth restrictionî [tiab] OR ìfoetal growth restrictionî [tiab] OR ìfetal growth restrictedî [tiab] OR ìfoetal growth restrictedî [tiab] OR ìfetal growth retardationî [tiab] OR ìfoetal growth retardationî [tiab] OR SGA [tiab] OR SFGA [tiab] OR ìsmall for gestational ageî [tiab] OR ìsmall-for-gestational-ageî [tiab] OR ìsmall-for-gestational ageî [tiab] OR ìsmall for gestationî [tiab] OR ìsmall-for-gestationî [tiab]

50. VSGA [tiab] OR ìvery-small-for-gestational-ageî [tiab] OR ìvery-small-for-gestational ageî [tiab] OR SFD [tiab] OR ìsmall for datesî [tiab] OR ìsmall-for-datesî [tiab] OR ìweight for datesî [tiab] OR ìweight for gestational ageî [tiab] OR ìweight for age at deliveryî [tiab] OR ìweight at deliveryî [tiab]

51. ìbirthweight for datesî [tiab] OR ìbirthweight for gestational ageî [tiab] OR ìbirthweight for age at deliveryî [tiab] OR ìbirth weight for datesî [tiab] OR ìbirth weight for gestational ageî [tiab] OR ìbirth weight for age at deliveryî [tiab] OR ìbirth-weight for datesî [tiab] OR ìbirth-weight for gestational ageî [tiab] OR ìbirth-weight for age at deliveryî [tiab]

52. LBW [tiab] OR ìlow BWî [tiab] OR ìlow birth weightî [tiab] OR ìlow birth-weightî [tiab] OR ìlow-birth weightî [tiab] OR ìlow-birth-weightî [tiab] OR ìlow birthweightî [tiab] OR ìlow-birthweightî [tiab] OR ìlower BWî [tiab] OR ìlower birth weightî [tiab] OR ìlower birth-weightî [tiab] OR ìlower-birth weightî [tiab] OR ìlower-birth-weightî [tiab] OR ìlower birthweightî [tiab] OR ìlower-birthweightî [tiab]

53. ìreduced birth weightî [tiab] OR ìreduced birthweightî [tiab] OR ìreduced birth-weightî [tiab] OR VLBW [tiab] OR ìvery-low birthweightî [tiab] OR ìvery-low birth weightî [tiab] OR ìvery-low birth-weightî [tiab] OR ìvery-low-birthweightî [tiab] OR ìvery-low-birth-weightî [tiab] OR ELBW [tiab] OR ìextremely-low birthweightî [tiab] OR ìextremely-low birth weightî [tiab] OR ìextremely-low birth-weightî [tiab] OR ìextremely-low-birthweightî [tiab] OR ìextremely-low-birth-weightî [tiab]

54. Stillbirth [Mesh] OR stillbirth* [tiab] OR still birth* [tiab] OR stillborn* [tiab] OR still born* [tiab] OR abortion* [tiab] OR miscarriage* [tiab] OR pregnancy death* [tiab] OR pregnancy loss* [tiab] OR pregnancy demise* [tiab] OR pregnancy mortalit* [tiab] OR gestational death* [tiab] OR gestational loss* [tiab] OR gestational demise* [tiab] OR "gestational mortalit*" [tiab]

55. Fetal Death [Mesh] OR "fetal death*" [tiab] OR "fetal loss*" [tiab] OR fetal demise* [tiab] OR fetal mortalit* [tiab] OR "foetal death*" [tiab] OR "foetal loss*" [tiab] OR foetal demise* [tiab] OR foetal mortalit* [tiab] OR "obstetric death*" [tiab] OR obstetric loss* [tiab] OR obstetric demise* [tiab] OR obstetric mortalit* [tiab] OR obstetrical death* [tiab] OR obstetrical loss* [tiab] OR obstetrical demise* [tiab] OR obstetrical mortalit* [tiab]

56. "labor death*" [tiab] OR labor loss* [tiab] OR labor demise* [tiab] OR labor mortalit* [tiab] OR labour death* [tiab] OR labour loss* [tiab] OR labour demise* [tiab] OR labour mortalit* [tiab] OR birth death* [tiab] OR birth loss* [tiab] OR birth demise* [tiab] OR birth mortalit* [tiab]

57. delivery death* [tiab] OR delivery loss* [tiab] OR delivery demise* [tiab] OR delivery mortalit* [tiab] OR neonate death* [tiab] OR neonate loss* [tiab] OR neonate demise* [tiab] OR neonate mortalit* [tiab] OR "newborn death*" [tiab] OR "newborn loss*" [tiab] OR "newborn demise*" [tiab] OR newborn mortalit* [tiab]

58. new-born death* [tiab] OR new-born loss* [tiab] OR new-born demise* [tiab] OR new-born mortalit* [tiab] OR "new born death*" [tiab] OR "new born loss*" [tiab] OR "new born demise*" [tiab] OR "new born mortalit*" [tiab] OR "infant death*" [tiab] OR infant loss* [tiab] OR infant demise* [tiab] OR infant mortalit* [tiab] OR reproductive death* [tiab] OR "reproductive loss*" [tiab] OR reproductive demise* [tiab] OR reproductive mortalit* [tiab]

59. prelabour death* [tiab] OR prelabour loss* [tiab] OR prelabour demise* [tiab] OR prelabour mortalit* [tiab] OR prelabor death* [tiab] OR prelabor loss* [tiab] OR prelabor demise* [tiab] OR prelabor mortalit* [tiab] OR pre-labour death* [tiab] OR pre-labour loss* [tiab] OR pre-labour demise* [tiab] OR pre-labour mortalit* [tiab] OR pre-labor death* [tiab] OR pre-labor loss* [tiab] OR pre-labor demise* [tiab] OR pre-labor mortalit* [tiab]

60. "intrauterine death*" [tiab] OR intrauterine loss* [tiab] OR intrauterine demise* [tiab] OR intrauterine mortalit* [tiab] OR intra-uterine death* [tiab] OR intra-uterine loss* [tiab] OR intra-uterine demise* [tiab] OR intra-uterine mortalit* [tiab] OR antenatal death* [tiab] OR antenatal loss* [tiab] OR antenatal demise* [tiab] OR antenatal mortalit* [tiab] OR ante-natal death* [tiab] OR ante-natal loss* [tiab] OR ante-natal demise* [tiab] OR ante-natal mortalit* [tiab]

61. prenatal death* [tiab] OR prenatal loss* [tiab] OR prenatal demise* [tiab] OR prenatal mortalit* [tiab] OR pre-natal death* [tiab] OR pre-natal loss* [tiab] OR pre-natal demise* [tiab] OR pre-natal mortalit* [tiab] OR "perinatal death" [tiab] OR "perinatal deaths" [tiab] OR perinatal loss* [tiab] OR perinatal demise* [tiab] OR "perinatal mortality" [tiab] OR "perinatal mortalities" [tiab] OR peri-natal death* [tiab] OR peri-natal loss* [tiab] OR peri-natal demise* [tiab] OR peri-natal mortalit* [tiab]

62. "neonatal death" [tiab] OR "neonatal deaths" [tiab] OR neonatal loss* [tiab] OR neonatal demise* [tiab] OR "neonatal mortality" [tiab] OR "neonatal mortalities" [tiab] OR neo-natal

death* [tiab] OR neo-natal loss* [tiab] OR neo-natal demise* [tiab] OR neo-natal mortalit* [tiab] OR postnatal death* [tiab] OR postnatal loss* [tiab] OR postnatal demise* [tiab] OR postnatal mortalit* [tiab] OR post-natal death* [tiab] OR post-natal loss* [tiab] OR post-natal demise* [tiab] OR post-natal mortalit* [tiab]

63. antepartum death* [tiab] OR antepartum loss* [tiab] OR antepartum demise* [tiab] OR antepartum mortalit* [tiab] OR ante-partum death* [tiab] OR ante-partum loss* [tiab] OR ante-partum demise* [tiab] OR ante-partum mortalit* [tiab] OR intrapartum death* [tiab] OR intrapartum loss* [tiab] OR intrapartum demise* [tiab] OR intrapartum mortalit* [tiab] OR intra-partum death* [tiab] OR intra-partum loss* [tiab] OR intra-partum demise* [tiab] OR intra-partum mortalit* [tiab]

64. peripartum death* [tiab] OR peripartum loss* [tiab] OR peripartum demise* [tiab] OR peripartum mortalit* [tiab] OR peri-partum death* [tiab] OR peri-partum loss* [tiab] OR peri-partum demise* [tiab] OR peri-partum mortalit* [tiab] OR postpartum death* [tiab] OR postpartum loss* [tiab] OR postpartum demise* [tiab] OR postpartum mortalit* [tiab] OR post-partum death* [tiab] OR post-partum loss* [tiab] OR post-partum demise* [tiab] OR post-partum mortalit* [tiab]

65. #1 or #2 or #3 or #4 or #5 or #6 or #7 or #8 or #9 or #10 or #11 or #12 or #13 or #14 or #15 or #16 or #17 or #18 or #19 or #20 or #21 or #22 or #23 or #24 or #25 or #26 or #27 or #28 or #29 or #30 or #31 or #32 or #33 or #34 or #35 or #36 or #37 or #38 or #39 or #40 or #41 or #42 or #43 or #44 or #45 or #46 or #47 or #48 or #49 or #50 or #51 or #52 or #53 or #54 or #55 or #56 or #57 or #58 or #59 or #60 or #61 or #62 or #63 or #64

66. "Integrase strand transfer inhibitor" [tiab] OR INSTI [tiab] OR Dolutegravir [tiab] OR DTG [tiab] OR Tivicay [tiab] OR Isentress [tiab] OR Vitekta [tiab]

67. "Formyl peptide receptor 1" [tiab] OR Fuzeon [tiab] OR FPR1 [tiab] OR ENF [tiab] OR Seizentry [tiab] OR Celsentri [tiab]

68. Ziagen [tiab] OR Videx [tiab] OR Emtriva [tiab] OR Coviracil [tiab] OR Zerit [tiab] OR Viread [tiab] OR Vemlidy [tiab] OR Retrovir [tiab] OR Azidothymidine [tiab]

69. "Diarylpyrimidine analogue" [tiab] OR Rescriptor [tiab] OR Sustiva [tiab] OR Intelence [tiab] OR TMC125 [tiab] OR DAPY [tiab] OR Viramune [tiab] OR Edurant [tiab] OR TMC278 [tiab]

70. Reyataz [tiab] OR APV [tiab] OR Agenerase [tiab] OR Prezista [tiab] OR Lexiva [tiab] OR Telzir [tiab] OR Crixivan [tiab] OR LPV/r [tiab] OR ABT-378 [tiab] OR Kaletra [tiab] OR Norvir [tiab] OR Viracept [tiab] OR AG1343 [tiab] OR Invirase [tiab] OR Fortovase [tiab] OR Aptivus [tiab]

71. "Rilpivirine plus dolutegravir" [tiab] OR "Raltegravir plus lamivudine" [tiab] OR "Abacavir plus lamivudine plus dolutegravir" [tiab] OR "Emtricitabine plus tenofovir alafenamide" [tiab] OR "Emtricitabine plus rilpivirine plus tenofovir alafenamide" [tiab] OR "Atazanavir plus cobicistat" [tiab] OR "Darunavir plus cobicistat" [tiab]

72. Juluca [tiab] OR Dutrebis [tiab] OR Stribild [tiab] OR Triumeq [tiab] OR Odefsey [tiab] OR Complera [tiab] OR Descovy [tiab] OR Genvoya [tiab] OR Evotaz [tiab] OR Prezcobix [tiab]

73. "Elvitegravir, Cobicistat, Emtricitabine, Tenofovir Disoproxil Fumarate Drug Combination" [mh] OR "Raltegravir Potassium" [mh] OR Zidovudine [mh] OR Delavirdine [mh] OR "Atazanavir Sulfate" [mh] OR "Emtricitabine, Rilpivirine, Tenofovir Drug Combination" [mh]

74. #66 or #67 or #68 or #69 or #70 or #71 or #72 or #73

75. #65 AND #74

76. 2018/04:2020/04 [dp]

77. #75 AND #76

Search strategy for “pregnancy outcome OR specific perinatal outcomes AND new new antiretroviral therapy”
Database and platform: Pubmed (via https://www.ncbi.nlm.nih.gov/pubmed/)
Latest search date: 30 June 2020.

1. Pregnancy Outcome [Mesh] OR Pregnancy Complications, Infectious [Mesh] OR "pregnancy outcome*" [tiab] OR "pregnancy complication*" [tiab] OR pregnancy consequence* [tiab] OR "pregnancy characteristic" [tiab] OR "pregnancy characteristics" [tiab] OR pregnancy event* [tiab] OR pregnancy result* [tiab] OR pregnancy problem* [tiab] OR pregnancy morbidit* [tiab] OR pregnancy sequelae [tiab]

2. gestational outcome* [tiab] OR gestational complication* [tiab] OR gestational consequence* [tiab] OR gestational characteristic* [tiab] OR gestational event* [tiab] OR gestational result* [tiab] OR gestational problem* [tiab] OR gestational morbidit* [tiab] OR gestational sequelae [tiab]

3. "fetal outcome" [tiab] OR "fetal outcomes" [tiab] OR "fetal complication" [tiab] OR "fetal complications" [tiab] OR fetal consequence* [tiab] OR fetal characteristic* [tiab] OR fetal event* [tiab] OR fetal result* [tiab] OR fetal problem* [tiab] OR fetal morbidit* [tiab] OR fetal sequelae [tiab]

4. foetal outcome* [tiab] OR foetal complication* [tiab] OR foetal consequence* [tiab] OR foetal characteristic* [tiab] OR foetal event* [tiab] OR foetal result* [tiab] OR foetal problem* [tiab] OR foetal morbidit* [tiab] OR foetal sequelae [tiab]

5. "obstetric outcome" [tiab] OR "obstetric outcomes" [tiab] OR "obstetric complication" [tiab] OR "obstetric complications" [tiab] OR obstetric consequence* [tiab] OR obstetric characteristic* [tiab] OR obstetric event* [tiab] OR obstetric result* [tiab] OR "obstetric problem" [tiab] OR "obstetric problems" [tiab] OR obstetric morbidit* [tiab] OR obstetric sequelae [tiab]

6. obstetrical outcome* [tiab] OR "obstetrical complication" [tiab] OR "obstetrical complications" [tiab] OR obstetrical consequence* [tiab] OR obstetrical characteristic* [tiab] OR obstetrical event* [tiab] OR obstetrical result* [tiab] OR "obstetrical problem" [tiab] OR "obstetrical problems" [tiab] OR obstetrical morbidit* [tiab] OR obstetrical sequelae [tiab]

7. "labor outcome" [tiab] OR "labor outcomes" [tiab] OR "labor complication" [tiab] OR "labor complications" [tiab] OR "labor consequence" [tiab] OR "labor consequences" [tiab] OR labor characteristic* [tiab] OR labor event* [tiab] OR labor result* [tiab] OR labor problem* [tiab] OR labor morbidit* [tiab] OR labor sequelae [tiab]

8. labour outcome* [tiab] OR labour complication* [tiab] OR "labour consequence" [tiab] OR "labour consequences" [tiab] OR labour characteristic* [tiab] OR labour event* [tiab] OR labour result* [tiab] OR labour problem* [tiab] OR labour morbidit* [tiab] OR labour sequelae [tiab]

9. "birth outcome" [tiab] OR "birth outcomes" [tiab] OR birth complication* [tiab] OR birth consequence* [tiab] OR birth characteristic* [tiab] OR birth event* [tiab] OR birth result* [tiab] OR birth problem* [tiab] OR birth morbidit* [tiab] OR birth sequelae [tiab]

10. delivery outcome* [tiab] OR delivery complication* [tiab] OR delivery consequence* [tiab] OR delivery characteristic* [tiab] OR delivery event* [tiab] OR delivery result* [tiab] OR delivery problem* [tiab] OR delivery morbidit* [tiab] OR delivery sequelae [tiab]

11. neonate outcome* [tiab] OR neonate complication* [tiab] OR neonate consequence* [tiab] OR neonate characteristic* [tiab] OR neonate event* [tiab] OR neonate result* [tiab] OR neonate problem* [tiab] OR neonate morbidit* [tiab] OR neonate sequelae [tiab]

12. newborn outcome* [tiab] OR newborn complication* [tiab] OR newborn consequence* [tiab] OR newborn characteristic* [tiab] OR newborn event* [tiab] OR newborn result* [tiab] OR newborn problem* [tiab] OR newborn morbidit* [tiab] OR newborn sequelae [tiab]

13. new-born outcome* [tiab] OR new-born complication* [tiab] OR new-born consequence* [tiab] OR new-born characteristic* [tiab] OR new-born event* [tiab] OR new-born result* [tiab] OR new-born problem* [tiab] OR new-born morbidit* [tiab] OR new-born sequelae [tiab]

14. new born outcome* [tiab] OR new born complication* [tiab] OR new born consequence* [tiab] OR new born characteristic* [tiab] OR new born event* [tiab] OR new born result* [tiab] OR new born problem* [tiab] OR new born morbidit* [tiab] OR new born sequelae [tiab]

15. infant outcome* [tiab] OR infant complication* [tiab] OR infant consequence* [tiab] OR infant characteristic* [tiab] OR infant event* [tiab] OR infant result* [tiab] OR infant problem* [tiab] OR infant morbidit* [tiab] OR infant sequelae [tiab]

16. reproductive outcome* [tiab] OR reproductive complication* [tiab] OR reproductive consequence* [tiab] OR reproductive characteristic* [tiab] OR reproductive event* [tiab] OR reproductive result* [tiab] OR reproductive problem* [tiab] OR reproductive morbidit* [tiab] OR reproductive sequelae [tiab]

17. prelabour outcome* [tiab] OR prelabour complication* [tiab] OR prelabour consequence* [tiab] OR prelabour characteristic* [tiab] OR prelabour event* [tiab] OR prelabour result* [tiab] OR prelabour problem* [tiab] OR prelabour morbidit* [tiab] OR prelabour sequelae [tiab]

18. prelabor outcome* [tiab] OR prelabor complication* [tiab] OR prelabor consequence* [tiab] OR prelabor characteristic* [tiab] OR prelabor event* [tiab] OR prelabor result* [tiab] OR prelabor problem* [tiab] OR prelabor morbidit* [tiab] OR prelabor sequelae [tiab]

19. pre-labour outcome* [tiab] OR pre-labour complication* [tiab] OR pre-labour consequence* [tiab] OR pre-labour characteristic* [tiab] OR pre-labour event* [tiab] OR pre-labour result* [tiab] OR pre-labour problem* [tiab] OR pre-labour morbidit* [tiab] OR pre-labour sequelae [tiab]

20. pre-labor outcome* [tiab] OR pre-labor complication* [tiab] OR pre-labor consequence* [tiab] OR pre-labor characteristic* [tiab] OR pre-labor event* [tiab] OR pre-labor result* [tiab] OR pre-labor problem* [tiab] OR pre-labor morbidit* [tiab] OR pre-labor sequelae [tiab]

21. intrauterine outcome* [tiab] OR intrauterine complication* [tiab] OR intrauterine consequence* [tiab] OR intrauterine characteristic* [tiab] OR intrauterine event* [tiab] OR

intrauterine result* [tiab] OR intrauterine problem* [tiab] OR intrauterine morbidit* [tiab] OR intrauterine sequelae [tiab]

22. intra-uterine outcome* [tiab] OR intra-uterine complication* [tiab] OR intra-uterine consequence* [tiab] OR intra-uterine characteristic* [tiab] OR intra-uterine event* [tiab] OR intra-uterine result* [tiab] OR intra-uterine problem* [tiab] OR intra-uterine morbidit* [tiab] OR intra-uterine sequelae [tiab]

23. antenatal outcome* [tiab] OR antenatal complication* [tiab] OR antenatal consequence* [tiab] OR antenatal characteristic* [tiab] OR antenatal event* [tiab] OR antenatal result* [tiab] OR antenatal problem* [tiab] OR antenatal morbidit* [tiab] OR antenatal sequelae [tiab]

24. ante-natal outcome* [tiab] OR ante-natal complication* [tiab] OR ante-natal consequence* [tiab] OR ante-natal characteristic* [tiab] OR ante-natal event* [tiab] OR ante-natal result* [tiab] OR ante-natal problem* [tiab] OR ante-natal morbidit* [tiab] OR ante-natal sequelae [tiab]

25. prenatal outcome* [tiab] OR prenatal complication* [tiab] OR prenatal consequence* [tiab] OR prenatal characteristic* [tiab] OR prenatal event* [tiab] OR prenatal result* [tiab] OR prenatal problem* [tiab] OR prenatal morbidit* [tiab] OR prenatal sequelae [tiab]

26. pre-natal outcome* [tiab] OR pre-natal complication* [tiab] OR pre-natal consequence* [tiab] OR pre-natal characteristic* [tiab] OR pre-natal event* [tiab] OR pre-natal result* [tiab] OR pre-natal problem* [tiab] OR pre-natal morbidit* [tiab] OR pre-natal sequelae [tiab]

27. perinatal outcome* [tiab] OR perinatal complication* [tiab] OR perinatal consequence* [tiab] OR perinatal characteristic* [tiab] OR perinatal event* [tiab] OR perinatal result* [tiab] OR perinatal problem* [tiab] OR perinatal morbidit* [tiab] OR perinatal sequelae [tiab]

28. peri-natal outcome* [tiab] OR peri-natal complication* [tiab] OR peri-natal consequence* [tiab] OR peri-natal characteristic* [tiab] OR peri-natal event* [tiab] OR peri-natal result* [tiab] OR peri-natal problem* [tiab] OR peri-natal morbidit* [tiab] OR peri-natal sequelae [tiab]
29. neonatal outcome* [tiab] OR neonatal complication* [tiab] OR neonatal consequence* [tiab] OR neonatal characteristic* [tiab] OR neonatal event* [tiab] OR neonatal result* [tiab] OR neonatal problem* [tiab] OR neonatal morbidit* [tiab] OR neonatal sequelae [tiab]

30. neo-natal outcome* [tiab] OR neo-natal complication* [tiab] OR neo-natal consequence* [tiab] OR neo-natal characteristic* [tiab] OR neo-natal event* [tiab] OR neo-natal result* [tiab] OR neo-natal problem* [tiab] OR neo-natal morbidit* [tiab] OR neo-natal sequelae [tiab]

31. postnatal outcome* [tiab] OR postnatal complication* [tiab] OR postnatal consequence* [tiab] OR postnatal characteristic* [tiab] OR postnatal event* [tiab] OR postnatal result* [tiab] OR postnatal problem* [tiab] OR postnatal morbidit* [tiab] OR postnatal sequelae [tiab]

32. post-natal outcome* [tiab] OR post-natal complication* [tiab] OR post-natal consequence* [tiab] OR post-natal characteristic* [tiab] OR post-natal event* [tiab] OR post-natal result* [tiab] OR post-natal problem* [tiab] OR post-natal morbidit* [tiab] OR post-natal sequelae [tiab]

33. antepartum outcome* [tiab] OR antepartum complication* [tiab] OR antepartum consequence* [tiab] OR antepartum characteristic* [tiab] OR antepartum event* [tiab] OR antepartum result* [tiab] OR antepartum problem* [tiab] OR antepartum morbidit* [tiab] OR antepartum sequelae [tiab]

34. ante-partum outcome* [tiab] OR ante-partum complication* [tiab] OR ante-partum consequence* [tiab] OR ante-partum characteristic* [tiab] OR ante-partum event* [tiab] OR ante-partum result* [tiab] OR ante-partum problem* [tiab] OR ante-partum morbidit* [tiab] OR ante-partum sequelae [tiab]

35. intrapartum outcome* [tiab] OR intrapartum complication* [tiab] OR intrapartum consequence* [tiab] OR intrapartum characteristic* [tiab] OR intrapartum event* [tiab] OR intrapartum result* [tiab] OR intrapartum problem* [tiab] OR intrapartum morbidit* [tiab] OR intrapartum sequelae [tiab]

36. intra-partum outcome* [tiab] OR intra-partum complication* [tiab] OR intra-partum consequence* [tiab] OR intra-partum characteristic* [tiab] OR intra-partum event* [tiab] OR intra-partum result* [tiab] OR intra-partum problem* [tiab] OR intra-partum morbidit* [tiab] OR intra-partum sequelae [tiab]

37. peripartum outcome* [tiab] OR peripartum complication* [tiab] OR peripartum consequence* [tiab] OR peripartum characteristic* [tiab] OR peripartum event* [tiab] OR peripartum result* [tiab] OR peripartum problem* [tiab] OR peripartum morbidit* [tiab] OR peripartum sequelae [tiab]

38. peri-partum outcome* [tiab] OR peri-partum complication* [tiab] OR peri-partum consequence* [tiab] OR peri-partum characteristic* [tiab] OR peri-partum event* [tiab] OR peri-partum result* [tiab] OR peri-partum problem* [tiab] OR peri-partum morbidit* [tiab] OR peri-partum sequelae [tiab]
39. postpartum outcome* [tiab] OR postpartum complication* [tiab] OR postpartum consequence* [tiab] OR postpartum characteristic* [tiab] OR postpartum event* [tiab] OR postpartum result* [tiab] OR postpartum problem* [tiab] OR postpartum morbidit* [tiab] OR postpartum sequelae [tiab]

40. post-partum outcome* [tiab] OR post-partum complication* [tiab] OR post-partum consequence* [tiab] OR post-partum characteristic* [tiab] OR post-partum event* [tiab] OR post-partum result* [tiab] OR post-partum problem* [tiab] OR post-partum morbidit* [tiab] OR post-partum sequelae [tiab]

41. Premature Birth [Mesh] OR Fetal Membranes, Premature Rupture [Mesh] OR Obstetric Labor, Premature [Mesh] OR Infant, Extremely Premature [Mesh] OR Infant, Premature [Mesh]

42. prematurity [tiab] OR ìgestational age at birthî [tiab] OR ìgestational age at deliveryî [tiab] OR PTB [tiab] OR PTBs [tiab] OR VPTB [tiab] OR VPTBs [tiab]

43. pre-terms [tiab] OR preterms [tiab] OR pre-term birth* [tiab] OR ìpreterm birth*î [tiab] OR ìpremature birth*î [tiab] OR PTL [tiab] OR PTLs [tiab] OR VPTL [tiab] OR VPTLs [tiab]

44. ìpre-term labor*î [tiab] OR ìpre-term labour*î [tiab] OR ìpre-term obstetric labor*î [tiab] OR ìpre-term obstetric labour*î [tiab] OR ìpreterm labor*î [tiab] OR ìpreterm labour*î [tiab] OR preterm obstetric labor* [tiab] OR ìpreterm obstetric labour*î [tiab] OR ìpremature labor*î [tiab] OR ìpremature

labour*î [tiab] OR premature obstetric labor* [tiab] OR ìpremature obstetric labour*î [tiab]

45. PTD [tiab] OR PTDs [tiab] OR VPTD [tiab] OR VPTDs [tiab] OR pre-term deliver* [tiab] OR preterm deliver* [tiab] OR ìpre-term infant*î [tiab] OR ìpreterm infant*î [tiab] OR premature deliver* [tiab] OR ìpremature infant*î [tiab] OR PROM [tiab] OR PPROM [tiab]

46. ìpreterm rupture of membranesî [tiab] OR preterm rupture of fetal membrane* [tiab] OR preterm rupture of foetal membrane* [tiab] OR pre-term rupture of membrane* [tiab] OR pre-term rupture of fetal membrane* [tiab] OR pre-term rupture of foetal membrane* [tiab] OR ìpremature rupture of membranesî [tiab] OR ìpremature rupture of fetal membranesî [tiab] OR ìpremature rupture of foetal membranesî [tiab]

47. Fetal Growth Retardation [Mesh] OR Infant, Low Birth Weight [Mesh] OR Infant, Very Low Birth Weight [Mesh] OR Infant, Extremely Low Birth Weight [Mesh] OR Infant, Small for Gestational Age [Mesh]

48. IUGR [tiab] OR FGR [tiab] OR ìintrauterine growth restrictionî [tiab] OR ìintra-uterine growth restrictionî [tiab] OR ìintrauterine growth restrictedî [tiab] OR ìintra-uterine growth restrictedî [tiab] OR ìintrauterine growth retardationî [tiab] OR ìintra-uterine growth retardationî [tiab]

49. ìfetal growth restrictionî [tiab] OR ìfoetal growth restrictionî [tiab] OR ìfetal growth restrictedî [tiab] OR ìfoetal growth restrictedî [tiab] OR ìfetal growth retardationî [tiab] OR ìfoetal growth retardationî [tiab] OR SGA [tiab] OR SFGA [tiab] OR ìsmall for gestational ageî [tiab] OR ìsmall-for-gestational-ageî [tiab] OR ìsmall-for-gestational ageî [tiab] OR ìsmall for gestationî [tiab] OR ìsmall-for-gestationî [tiab]

50. VSGA [tiab] OR ìvery-small-for-gestational-ageî [tiab] OR ìvery-small-for-gestational ageî [tiab] OR SFD [tiab] OR ìsmall for datesî [tiab] OR ìsmall-for-datesî [tiab] OR ìweight for datesî [tiab] OR ìweight for gestational ageî [tiab] OR ìweight for age at deliveryî [tiab] OR ìweight at deliveryî [tiab]

51. ìbirthweight for datesî [tiab] OR ìbirthweight for gestational ageî [tiab] OR ìbirthweight for age at deliveryî [tiab] OR ìbirth weight for datesî [tiab] OR ìbirth weight for gestational ageî [tiab] OR ìbirth weight for age at deliveryî [tiab] OR ìbirth-weight for datesî [tiab] OR ìbirth-weight for gestational ageî [tiab] OR ìbirth-weight for age at deliveryî [tiab]

52. LBW [tiab] OR ìlow BWî [tiab] OR ìlow birth weightî [tiab] OR ìlow birth-weightî [tiab] OR ìlow-birth weightî [tiab] OR ìlow-birth-weightî [tiab] OR ìlow birthweightî [tiab] OR ìlow-birthweightî [tiab] OR ìlower BWî [tiab] OR ìlower birth weightî [tiab] OR ìlower birth-weightî [tiab] OR ìlower-birth weightî [tiab] OR ìlower-birth-weightî [tiab] OR ìlower birthweightî [tiab] OR ìlower-birthweightî [tiab]

53. ìreduced birth weightî [tiab] OR ìreduced birthweightî [tiab] OR ìreduced birth-weightî [tiab] OR VLBW [tiab] OR ìvery-low birthweightî [tiab] OR ìvery-low birth weightî [tiab] OR ìvery-low birth-weightî [tiab] OR ìvery-low-birthweightî [tiab] OR ìvery-low-birth-weightî [tiab] OR ELBW [tiab] OR ìextremely-low birthweightî [tiab] OR ìextremely-low birth weightî [tiab] OR ìextremely-low birth-weightî [tiab] OR ìextremely-low-birthweightî [tiab] OR ìextremely-low-birth-weightî [tiab]

54. Stillbirth [Mesh] OR stillbirth* [tiab] OR still birth* [tiab] OR stillborn* [tiab] OR still born* [tiab] OR abortion* [tiab] OR miscarriage* [tiab] OR pregnancy death* [tiab] OR pregnancy loss* [tiab] OR pregnancy demise* [tiab] OR pregnancy mortalit* [tiab] OR gestational death* [tiab] OR gestational loss* [tiab] OR gestational demise* [tiab] OR "gestational mortalit*" [tiab]

55. Fetal Death [Mesh] OR "fetal death*" [tiab] OR "fetal loss*" [tiab] OR fetal demise* [tiab] OR fetal mortalit* [tiab] OR "foetal death*" [tiab] OR "foetal loss*" [tiab] OR foetal demise* [tiab] OR foetal mortalit* [tiab] OR "obstetric death*" [tiab] OR obstetric loss* [tiab] OR obstetric demise* [tiab] OR obstetric mortalit* [tiab] OR obstetrical death* [tiab] OR obstetrical loss* [tiab] OR obstetrical demise* [tiab] OR obstetrical mortalit* [tiab]

56. "labor death*" [tiab] OR labor loss* [tiab] OR labor demise* [tiab] OR labor mortalit* [tiab] OR labour death* [tiab] OR labour loss* [tiab] OR labour demise* [tiab] OR labour mortalit* [tiab] OR birth death* [tiab] OR birth loss* [tiab] OR birth demise* [tiab] OR birth mortalit* [tiab]

57. delivery death* [tiab] OR delivery loss* [tiab] OR delivery demise* [tiab] OR delivery mortalit* [tiab] OR neonate death* [tiab] OR neonate loss* [tiab] OR neonate demise* [tiab] OR neonate mortalit* [tiab] OR "newborn death*" [tiab] OR "newborn loss*" [tiab] OR "newborn demise*" [tiab] OR newborn mortalit* [tiab]

58. new-born death* [tiab] OR new-born loss* [tiab] OR new-born demise* [tiab] OR new-born mortalit* [tiab] OR "new born death*" [tiab] OR "new born loss*" [tiab] OR "new born demise*" [tiab] OR "new born mortalit*" [tiab] OR "infant death*" [tiab] OR infant loss* [tiab] OR infant demise* [tiab] OR infant mortalit* [tiab] OR reproductive death* [tiab] OR "reproductive loss*" [tiab] OR reproductive demise* [tiab] OR reproductive mortalit* [tiab]

59. prelabour death* [tiab] OR prelabour loss* [tiab] OR prelabour demise* [tiab] OR prelabour mortalit* [tiab] OR prelabor death* [tiab] OR prelabor loss* [tiab] OR prelabor demise* [tiab] OR prelabor mortalit* [tiab] OR pre-labour death* [tiab] OR pre-labour loss* [tiab] OR pre-labour demise* [tiab] OR pre-labour mortalit* [tiab] OR pre-labor death* [tiab] OR pre-labor loss* [tiab] OR pre-labor demise* [tiab] OR pre-labor mortalit* [tiab]

60. "intrauterine death*" [tiab] OR intrauterine loss* [tiab] OR intrauterine demise* [tiab] OR intrauterine mortalit* [tiab] OR intra-uterine death* [tiab] OR intra-uterine loss* [tiab] OR intra-uterine demise* [tiab] OR intra-uterine mortalit* [tiab] OR antenatal death* [tiab] OR antenatal loss* [tiab] OR antenatal demise* [tiab] OR antenatal mortalit* [tiab] OR ante-natal death* [tiab] OR ante-natal loss* [tiab] OR ante-natal demise* [tiab] OR ante-natal mortalit* [tiab]

61. prenatal death* [tiab] OR prenatal loss* [tiab] OR prenatal demise* [tiab] OR prenatal mortalit* [tiab] OR pre-natal death* [tiab] OR pre-natal loss* [tiab] OR pre-natal demise* [tiab] OR pre-natal mortalit* [tiab] OR "perinatal death" [tiab] OR "perinatal deaths" [tiab] OR perinatal loss* [tiab] OR perinatal demise* [tiab] OR "perinatal mortality" [tiab] OR "perinatal mortalities" [tiab] OR peri-natal death* [tiab] OR peri-natal loss* [tiab] OR peri-natal demise* [tiab] OR peri-natal mortalit* [tiab]

62. "neonatal death" [tiab] OR "neonatal deaths" [tiab] OR neonatal loss* [tiab] OR neonatal demise* [tiab] OR "neonatal mortality" [tiab] OR "neonatal mortalities" [tiab] OR neo-natal

death* [tiab] OR neo-natal loss* [tiab] OR neo-natal demise* [tiab] OR neo-natal mortalit* [tiab] OR postnatal death* [tiab] OR postnatal loss* [tiab] OR postnatal demise* [tiab] OR postnatal mortalit* [tiab] OR post-natal death* [tiab] OR post-natal loss* [tiab] OR post-natal demise* [tiab] OR post-natal mortalit* [tiab]

63. antepartum death* [tiab] OR antepartum loss* [tiab] OR antepartum demise* [tiab] OR antepartum mortalit* [tiab] OR ante-partum death* [tiab] OR ante-partum loss* [tiab] OR ante-partum demise* [tiab] OR ante-partum mortalit* [tiab] OR intrapartum death* [tiab] OR intrapartum loss* [tiab] OR intrapartum demise* [tiab] OR intrapartum mortalit* [tiab] OR intra-partum death* [tiab] OR intra-partum loss* [tiab] OR intra-partum demise* [tiab] OR intra-partum mortalit* [tiab]

64. peripartum death* [tiab] OR peripartum loss* [tiab] OR peripartum demise* [tiab] OR peripartum mortalit* [tiab] OR peri-partum death* [tiab] OR peri-partum loss* [tiab] OR peri-partum demise* [tiab] OR peri-partum mortalit* [tiab] OR postpartum death* [tiab] OR postpartum loss* [tiab] OR postpartum demise* [tiab] OR postpartum mortalit* [tiab] OR post-partum death* [tiab] OR post-partum loss* [tiab] OR post-partum demise* [tiab] OR post-partum mortalit* [tiab]

65. #1 or #2 or #3 or #4 or #5 or #6 or #7 or #8 or #9 or #10 or #11 or #12 or #13 or #14 or #15 or #16 or #17 or #18 or #19 or #20 or #21 or #22 or #23 or #24 or #25 or #26 or #27 or #28 or #29 or #30 or #31 or #32 or #33 or #34 or #35 or #36 or #37 or #38 or #39 or #40 or #41 or #42 or #43 or #44 or #45 or #46 or #47 or #48 or #49 or #50 or #51 or #52 or #53 or #54 or #55 or #56 or #57 or #58 or #59 or #60 or #61 or #62 or #63 or #64

66. Cobicistat [tiab] OR Tubost [tiab] OR COBI [tiab] OR Rezolsta [tiab] OR QUAD [tiab] OR Epivir [tiab] OR Temixys [tiab] OR Cimduo [tiab] OR Selzentry [tiab] OR Doravirine [tiab] OR DOR [tiab] OR Pifeltro [tiab] OR Ibalizumab-uiyk [tiab] OR Hu5A8 [tiab] OR IBA [tiab] OR Ibalizumab [tiab] OR "TMB-355" [tiab] OR "TNX-355" [tiab] OR Trogarzo [tiab]

67. Bictegravir [tiab] OR BIC [tiab] OR "Bictegravir, emtricitabine, and tenofovir alafenamide fumarate" [tiab] OR "bictegravir sodium/emtricitabine/tenofovir alafenamide fumarate" [tiab] OR "BIC/FTC/TAF" [tiab] OR Biktarvy [tiab] OR Darunavir [tiab] OR Emtricitabine [tiab] OR "tenofovir alafenamide fumarate" [tiab] OR "darunavir ethanolate, cobicistat, emtricitabine, tenofovir alafenamide fumarate" [tiab] OR "DRV/COBI/FTC/TAF" [tiab]

68. Symtuza [tiab] OR "Dolutegravir and lamivudine" [tiab] OR "dolutegravir sodium/lamivudine" [tiab] OR "DTG/3TC" [tiab] OR Dovato [tiab] OR "Doravirine, lamivudine, and tenofovir disoproxil fumarate" [tiab] OR "doravirine/lamivudine/tenofovir disoproxil fumarate" [tiab] OR "DOR/3TC/TDF" [tiab] OR Delstrigo [tiab] OR "Efavirenz, lamivudine, and tenofovir disoproxil fumarate" [tiab] OR "EFV/3TC/TDF" [tiab]

69. Symfi [tiab] OR "Symfi Lo" [tiab] OR "Elvitegravir, cobicistat, emtricitabine, and tenofovir alafenamide fumarate" [tiab] OR "elvitegravir/cobicistat/emtricitabine/tenofovir alafenamide fumarate" [tiab] OR "EVG/COBI/FTC/TAF" [tiab] OR Genvoya [tiab] OR "TMC-114" [tiab] OR "TMC114" [tiab] OR Dideoxyinosine [tiab] OR Racivir [tiab] OR Heptovir [tiab] OR Hepitec [tiab]

70. Zerut [tiab] OR Estavudina [tiab] OR Sanilvudine [tiab] OR Stavudine [tiab] OR Apropovir [tiab] OR Stocrin [tiab] OR Zrivada [tiab] OR Aluvia [tiab] OR Aluviran [tiab] OR Koletra [tiab] OR Pentafuside [tiab] OR Didanosine [tiab] OR Lamivudine [tiab] OR Enfuvirtide [tiab] OR Maraviroc [tiab]

71. Cobicistat [mh] OR Darunavir [mh] OR Emtricitabine [mh] OR "Elvitegravir, Cobicistat, Emtricitabine, Tenofovir Disoproxil Fumarate Drug Combination" [mh] OR Didanosine [mh] OR Lamivudine [mh] OR Stavudine [mh] OR Enfuvirtide [mh] OR Maraviroc [mh]

72. Doravirine [nm] OR Ibalizumab [nm] OR Bictegravir [nm] OR "Bictegravir, Embitcitabine, Tenofovir Alafenamide" [nm] OR Symtuza [nm]

73. "Efavirenz, Lamivudine, Tenofovir Disoproxil Fumarate drug combination" [nm] OR Genvoya [nm] OR Racivir [nm] OR "Lopinavir-Ritonavir drug combination" [nm]

74. #66 or #67 or #68 or #69 or #70 or #71 or #72 or #73
75. #65 AND #74

76. 1980:2020 [dp]

77. #75 AND #

Supplementary Note 2

Quality Assessment of Studies

Adapted Newcastle-Ottawa quality assessment tool

A study can be awarded a maximum of one point (for items indicated with an asterisk) for each numbered criterion within the "Selection" and "Outcome" categories.

Selection (maximum 4 points)

1) Representativeness of the exposed cohort.
    - a) Truly representative of the pregnant population in the community. *
    - b) Somewhat representative of the pregnant population in the community.
    - c) Selected group of users, e.g. nurses, volunteers, teenage mothers.
    - d) No description of the derivation of the cohort.
2) Selection of the comparator cohort.
    - a) The comparator cohort is drawn from the same community as the exposed cohort. *
    - b) The comparator cohort is drawn from a different source than the exposed cohort.
    - c) No description of the derivation of the comparator cohort.
3) Ascertainment of exposure.
    - a) ART intake monitored as part of study. *
    - b) ART intake confirmed from secure medical records (e.g. hospital records). *
    - c) Structured interview-participant reported ART intake.
    - d) Written self-report.
    - e) No description.
4) Demonstration that outcome of interest was not present at start of study.
    - a) Yes. *
    - b) No.

Comparability (maximum 2 points)

1) Comparability of cohorts on the basis of the analysis. In the analysis:
    - a) Study controls for BMI, smoking, parity, and maternal age. *
    - b) Study controls for one or more additional factors: e.g. prior history of adverse pregnancy outcome, maternal hypertension, anaemia, illicit drug or alcohol use in pregnancy. *
    - c) Confounding factors not controlled for.

Outcome (maximum 3 points)

1) Ascertainment of outcome.
    - a) Outcome was confirmed following clinical observation of outcome by clinician, midwife or trained birth attendant. *
    - b) Medical records. *
    - c) Self-report.
    - d) No description.
2) Method used to assess gestational age.
    - a) Gestational age was determined according to early ultrasound (<14 weeks). *
    - b) Gestational age was determined by: late ultrasound (≥14 weeks 'gestation) or last normal menstrual period or neonatal assessment, e.g. Ballard score, or a combination of these methods.
    - c) No description.
3) Follow up of cohorts
    - a) Complete follow up - all subjects accounted for. *
    - b) Subjects lost to follow up unlikely to introduce bias, i.e. < 20 % lost to follow up. *
    - c) Follow up rate < 80% (lost to follow-up > 20%).
    - d) No description.

Supplementary Note 3

Classification of studies according to quality assessment

Good Quality	9 points - all requirements met
Average Quality	3 points in “Selection” and 3 points in “Outcome” sections
	≥2 points in the “Selection” and “Outcome” sections, as well as ≥1 point in the “Comparability” section.
Poor Quality	< 2 points in the “Selection” and/or “Outcome” sections.
	2 points in the “Selection” and “Outcome” sections, but no points in the “Comparability” section.

Supplementary Figures

Random-effects meta-analyses of risk of perinatal outcomes associated with pregnant women living with HIV, compared to HIV-negative women

Forest plots showing random-effects meta-analyses of risk of perinatal outcomes associated with pregnant women living with HIV receiving either no treatment (no ART), zidovudine monotherapy (monotherapy), cART initiated antenatally (antenatal cART) or preconception (preconception cART), each compared to HIV-negative women. Risk difference (RD) and 95% confidence intervals (CIs).

Women living with HIV receiving no treatment vs HIV-negative women

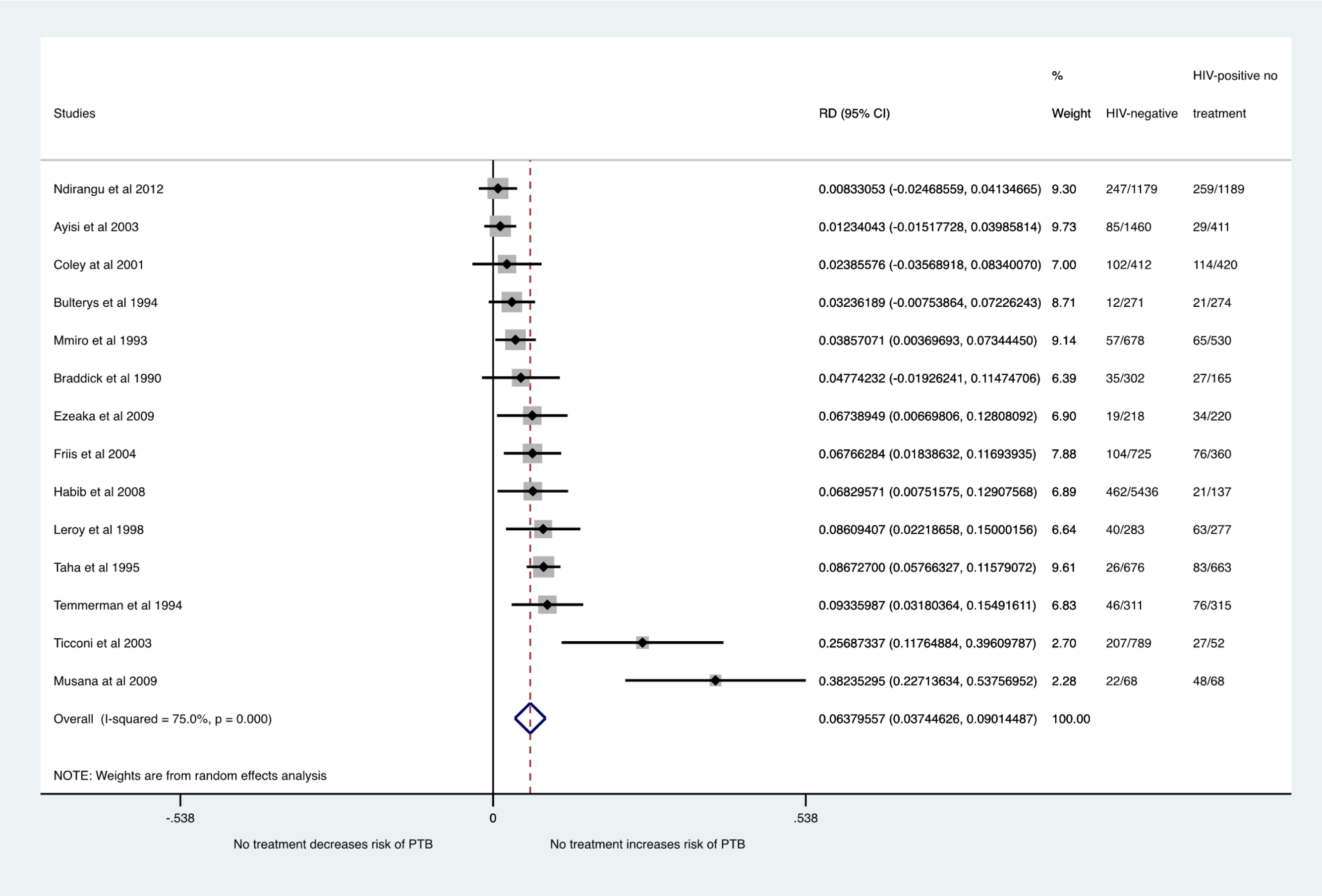

Figure 1: Preterm birth (PTB) in women living with HIV (WLHIV) receiving no treatment vs. HIV-negative women

Peters test for small-study effects: p = 0.168

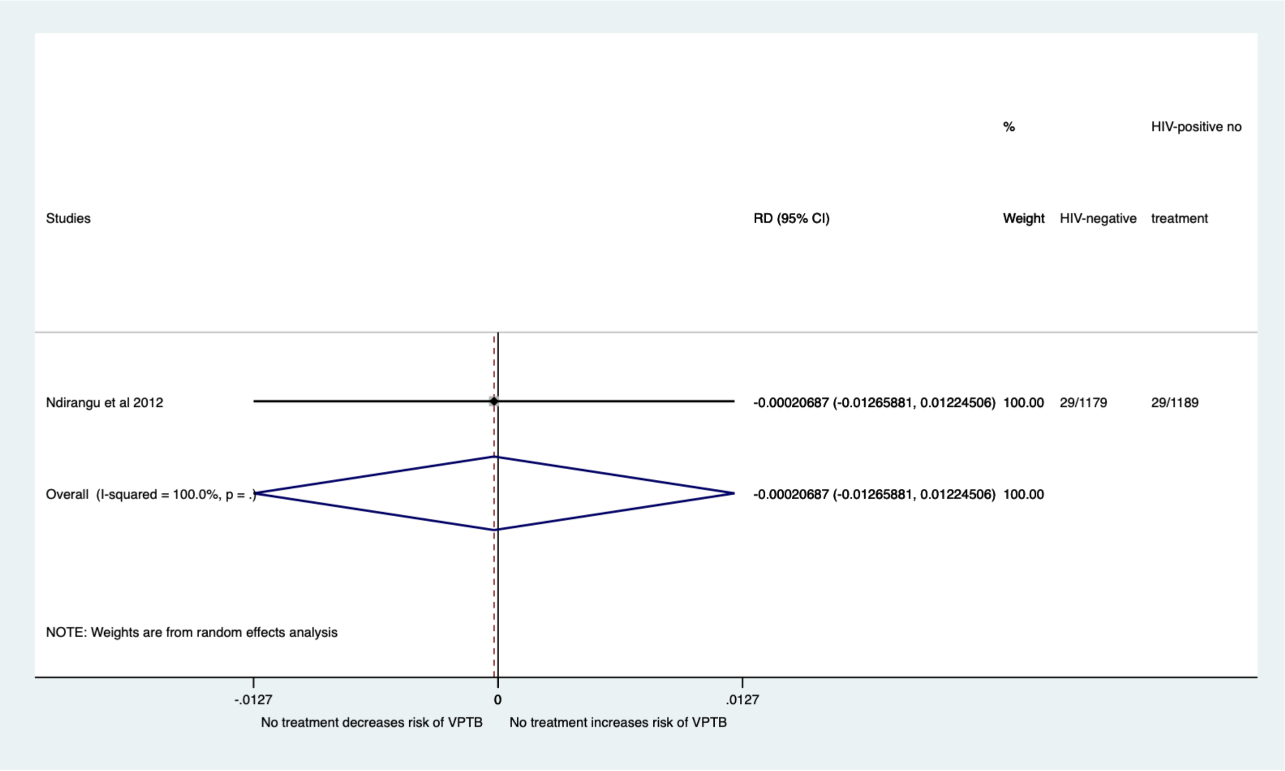

Figure 2: Very preterm birth (VPTB) in women living with HIV (WLHIV) receiving no treatment vs. HIV-negative women

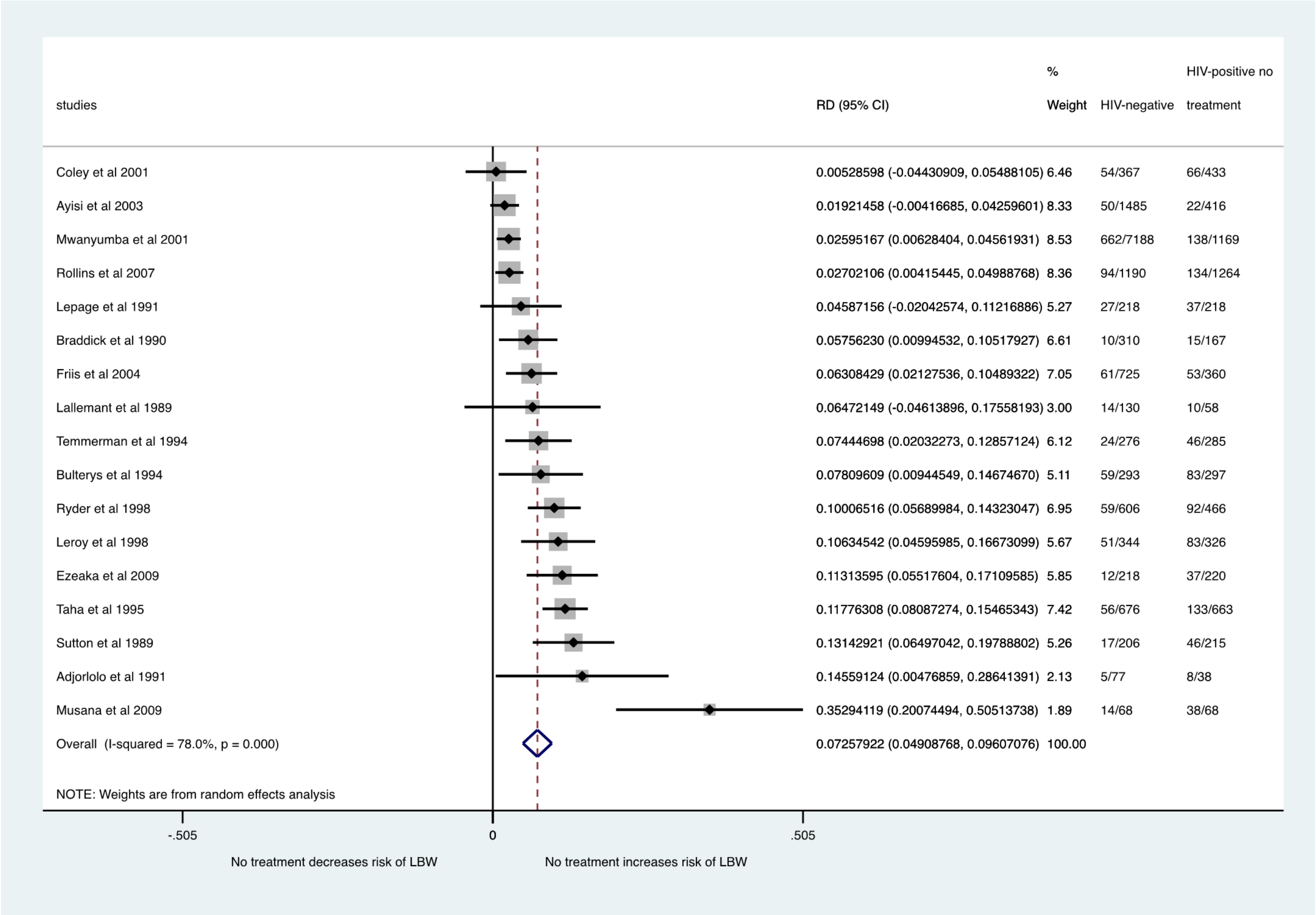

Figure 3: Low birth weight (LBW) in women living with HIV (WLHIV) receiving no treatment vs. HIV-negative women

Peters test for small-study effects: p = 0.110

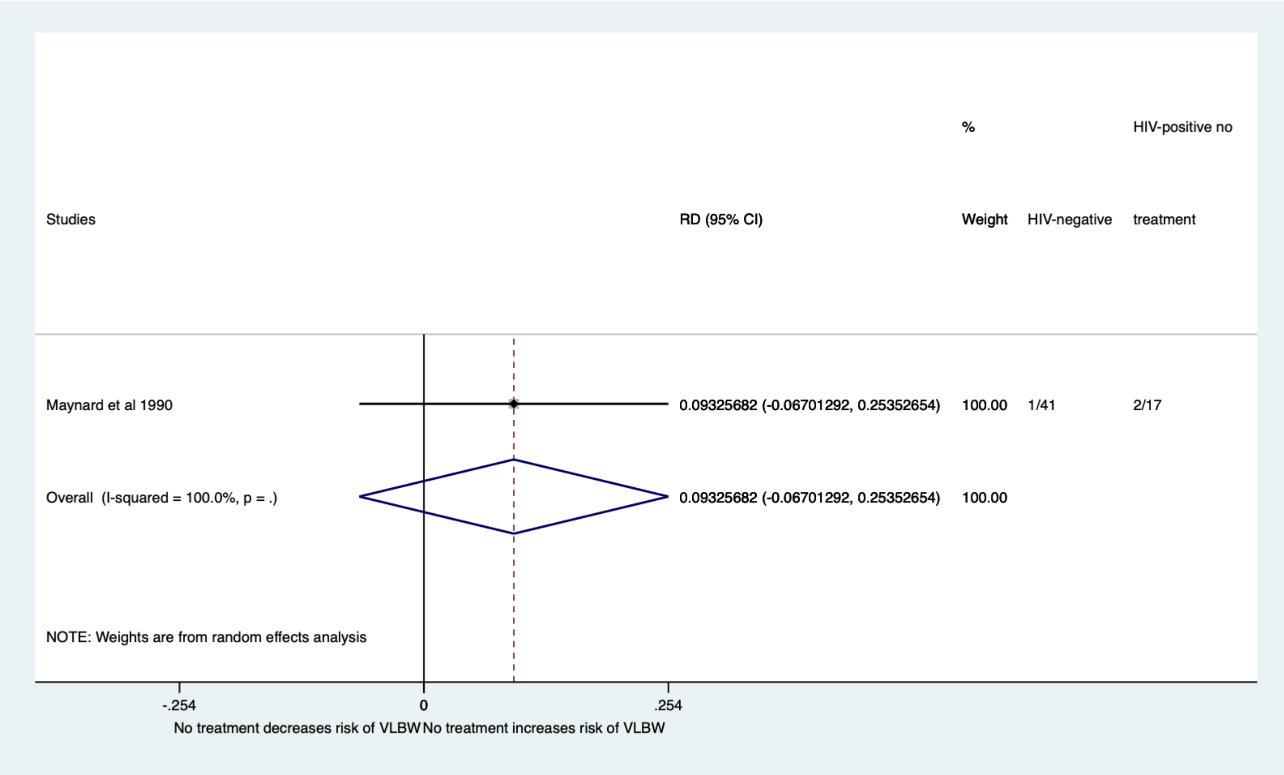

Figure 4: Very low birth weight (VLBW) in women living with HIV (WLHIV) receiving no treatment vs. HIV-negative women

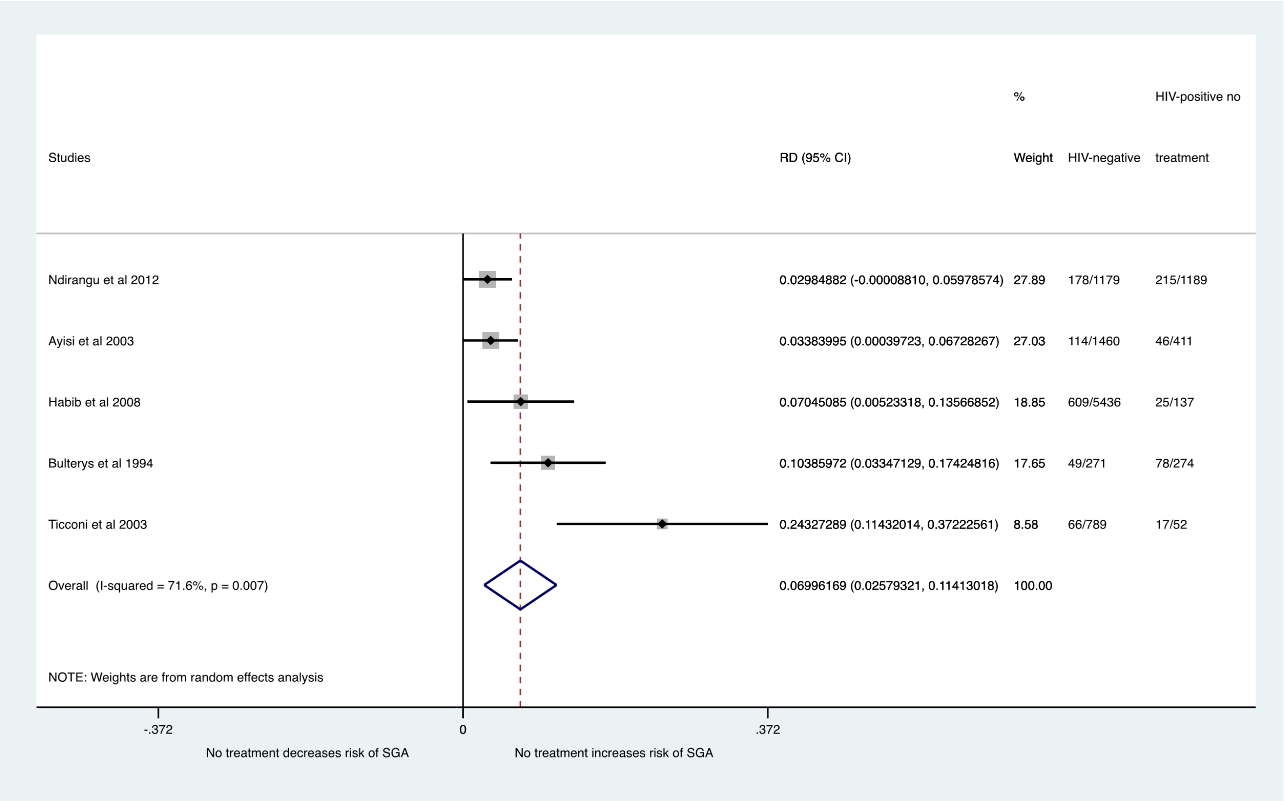

Figure 5: Small for gestational age (SGA) in women living with HIV (WLHIV) receiving no treatment vs. HIV-negative women

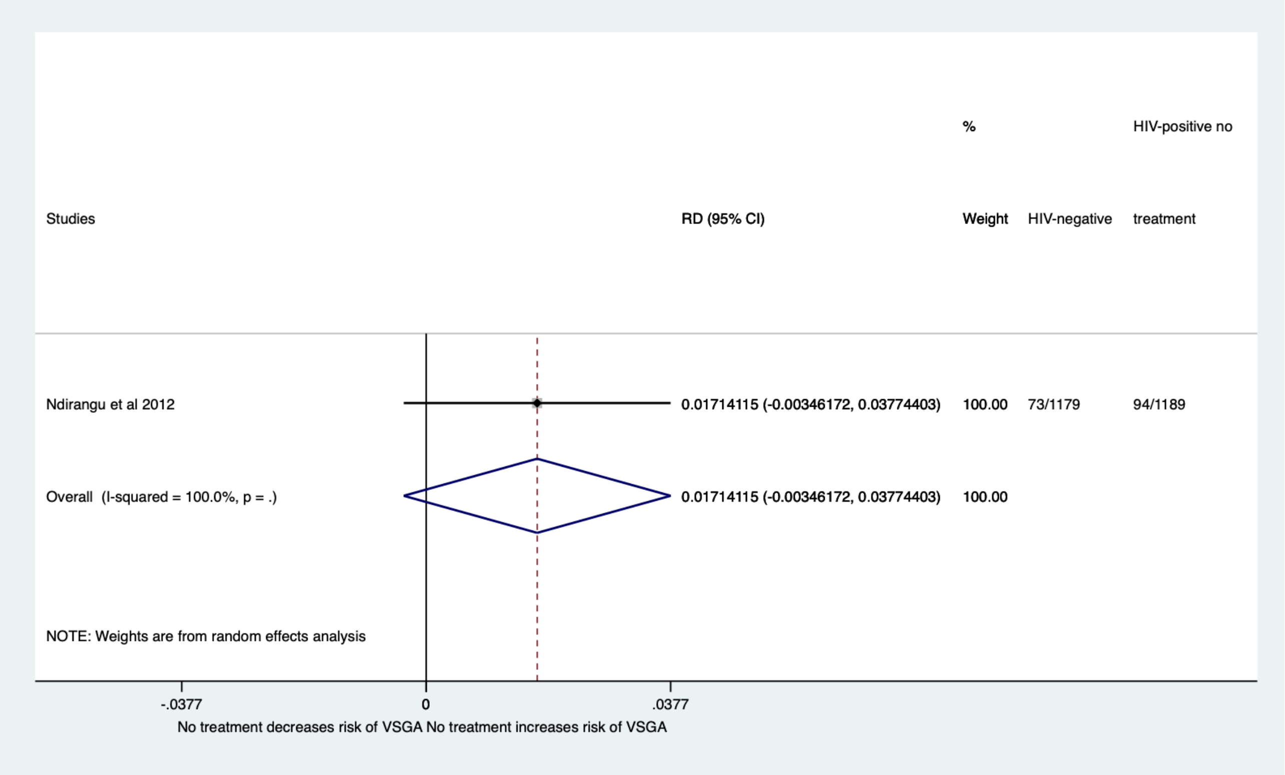

Figure 6: Very small for gestational age (VSGA) in women living with HIV (WLHIV) receiving no treatment vs. HIV-negative women

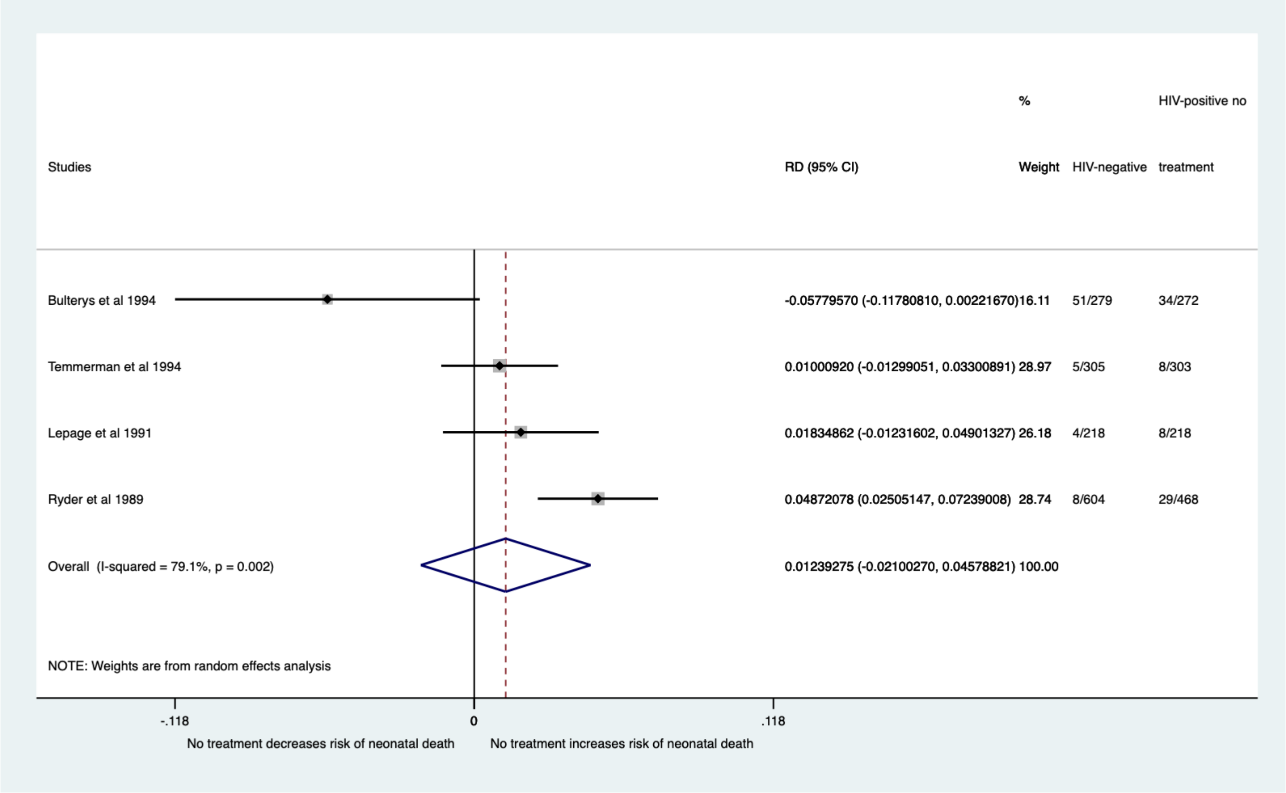

Figure 7: Neonatal death (NND) in women living with HIV (WLHIV) receiving no treatment vs. HIV-negative women

Women living with HIV receiving monotherapy vs HIV-negative women

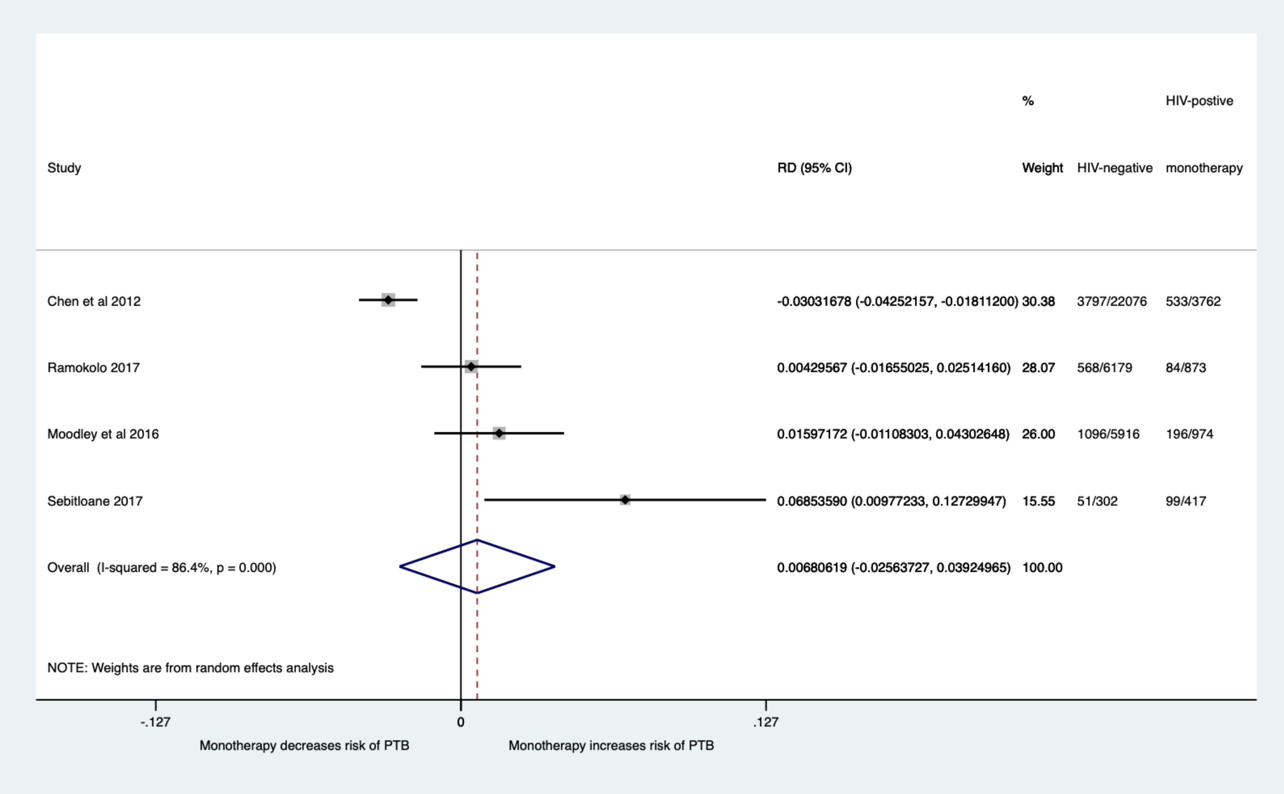

Figure 8: Preterm birth (PTB) in women living with HIV (WLHIV) receiving monotherapy vs. HIV-negative women

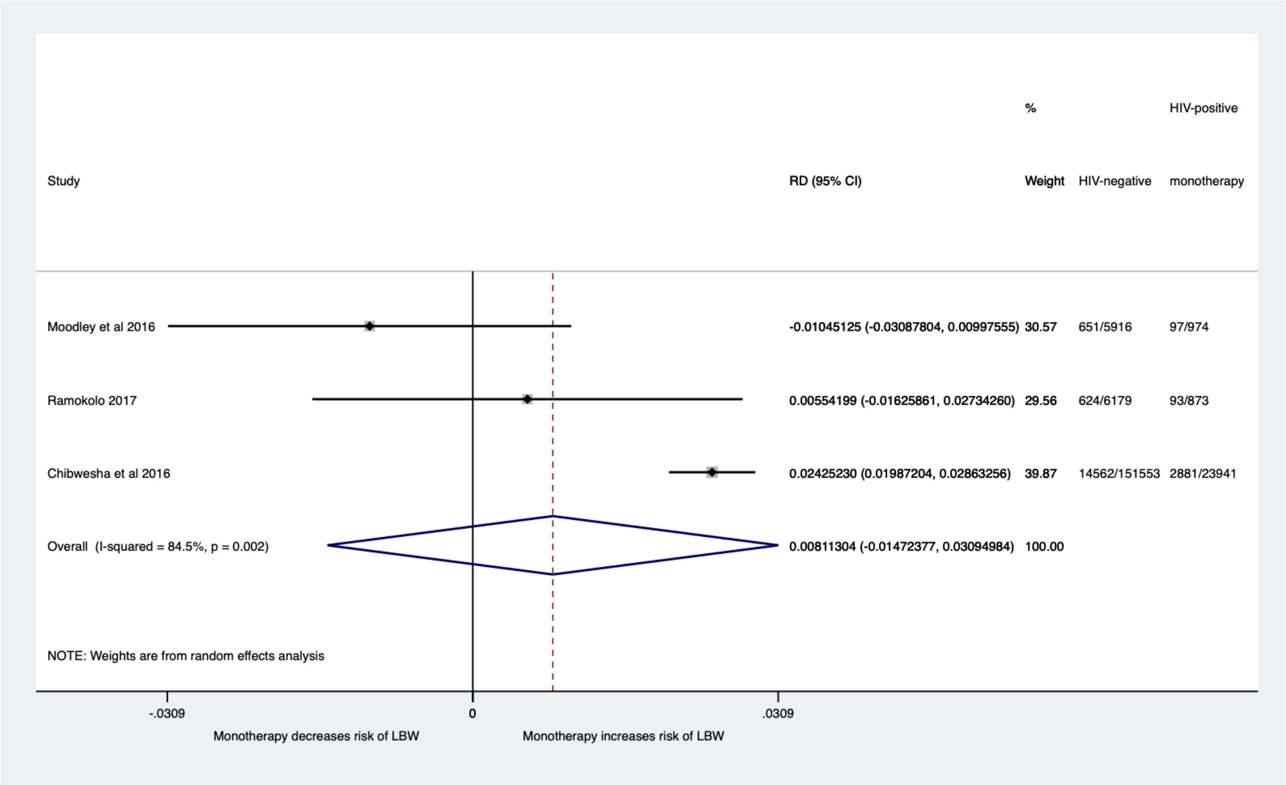

Figure 9: Low birth weight (LBW) in women living with HIV (WLHIV) receiving monotherapy vs. HIV-negative women

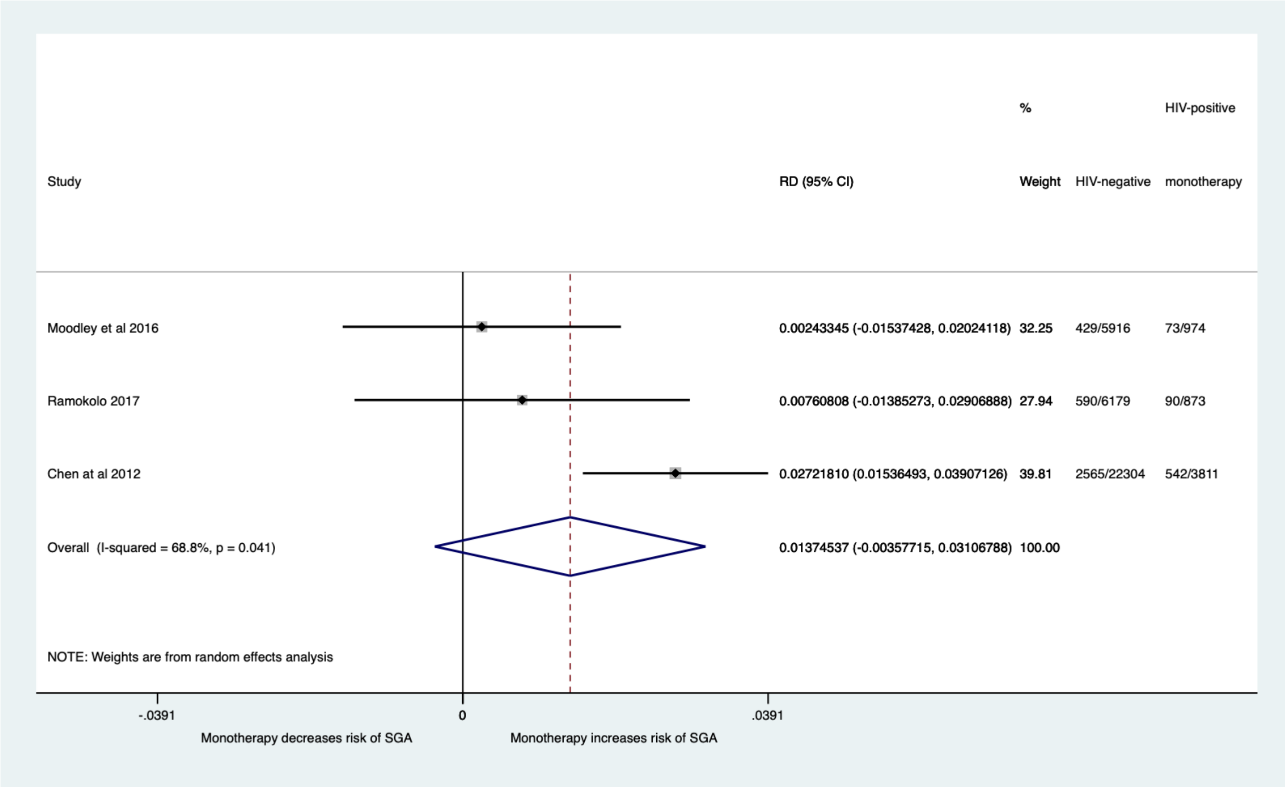

Figure 10: Small for gestational age (SGA) in women living with HIV (WLHIV) receiving monotherapy vs. HIV-negative women

Women living with HIV receiving antenatal cART vs HIV-negative women

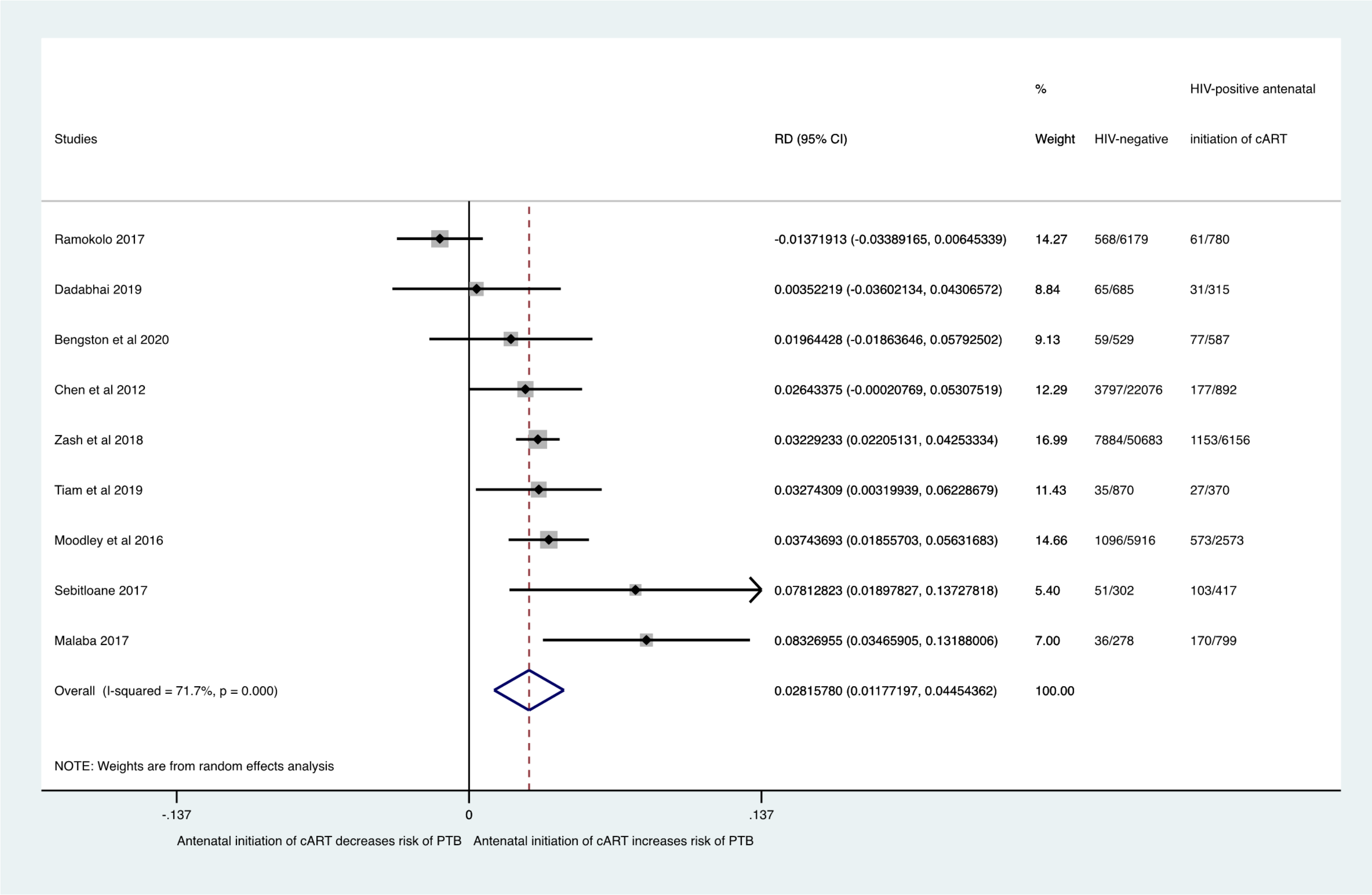

Figure 11: Preterm birth (PTB) in women living with HIV (WLHIV) receiving antenatal initiation of cART vs. HIV-negative women

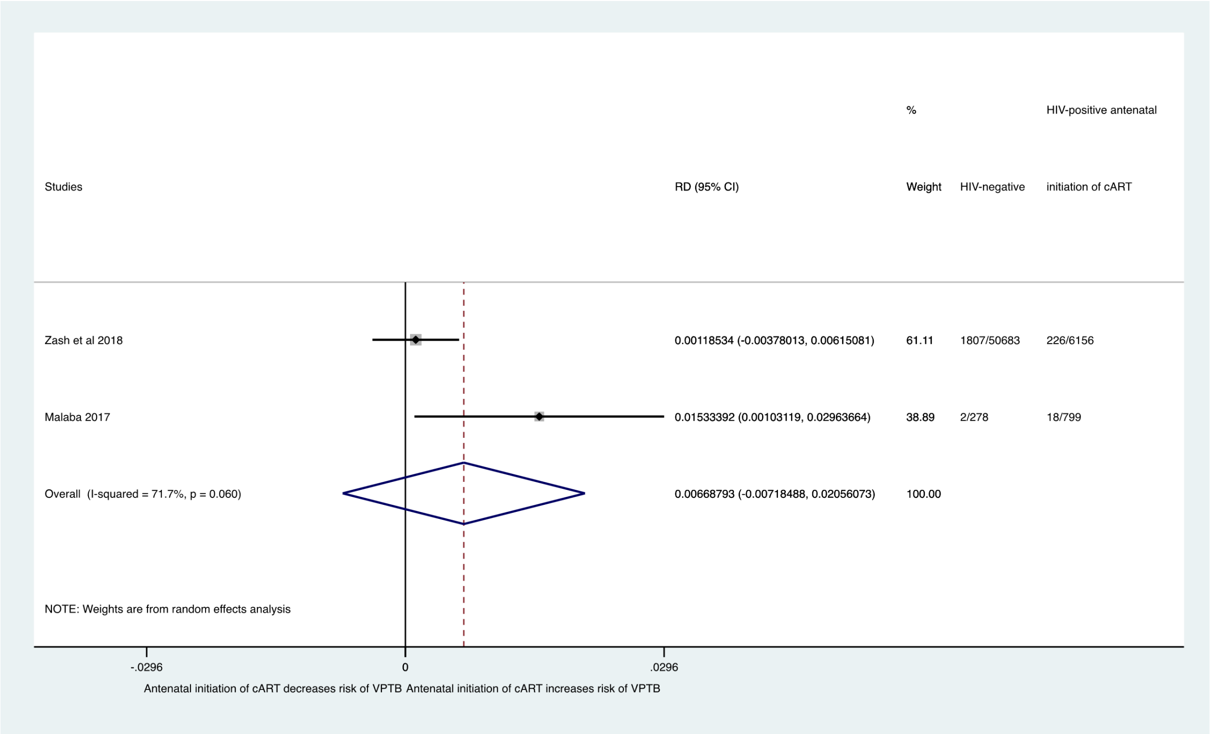

Figure 12: Very preterm birth (VPTB) in women living with HIV (WLHIV) receiving antenatal initiation of cART vs. HIV-negative women

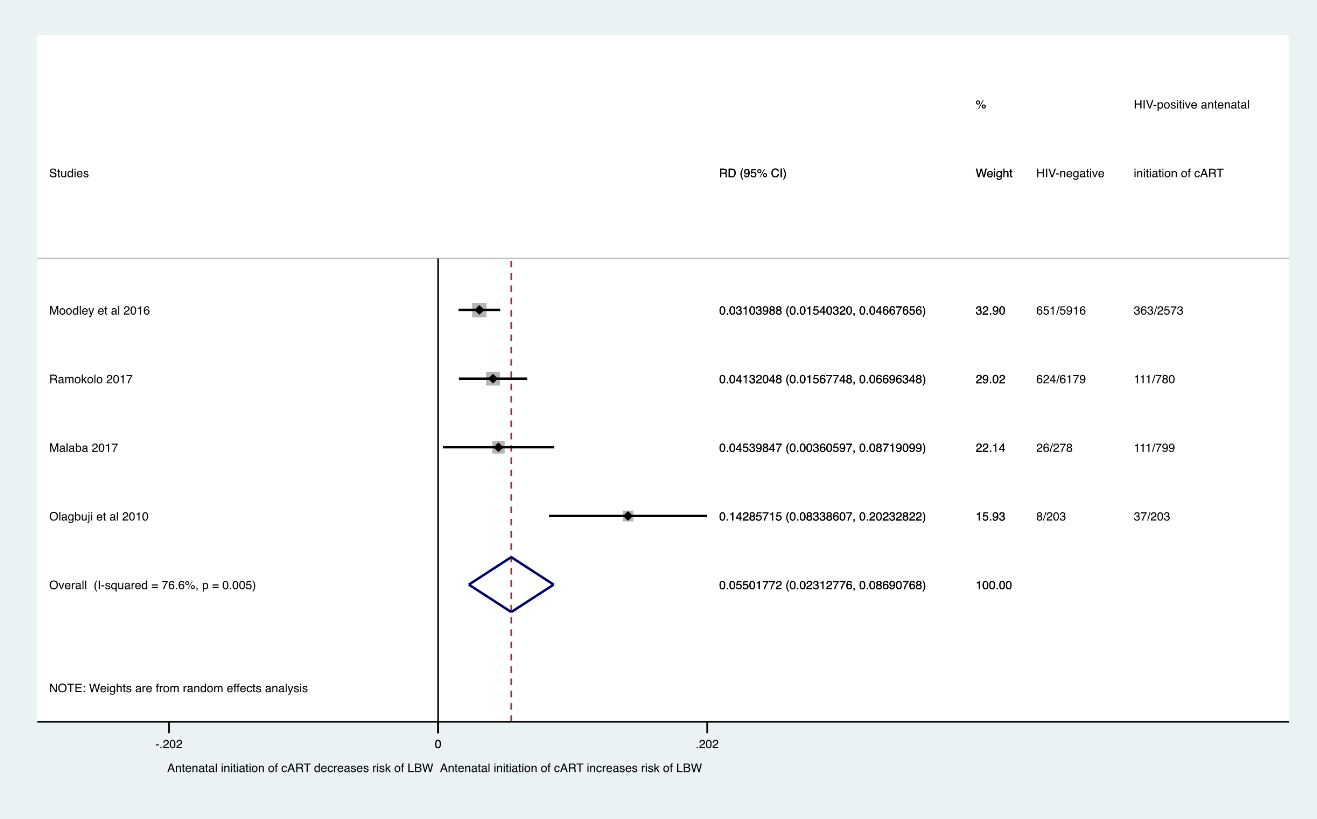

Figure 13: Low birth weight (LBW) in women living with HIV (WLHIV) receiving antenatal initiation of cART vs. HIV-negative women

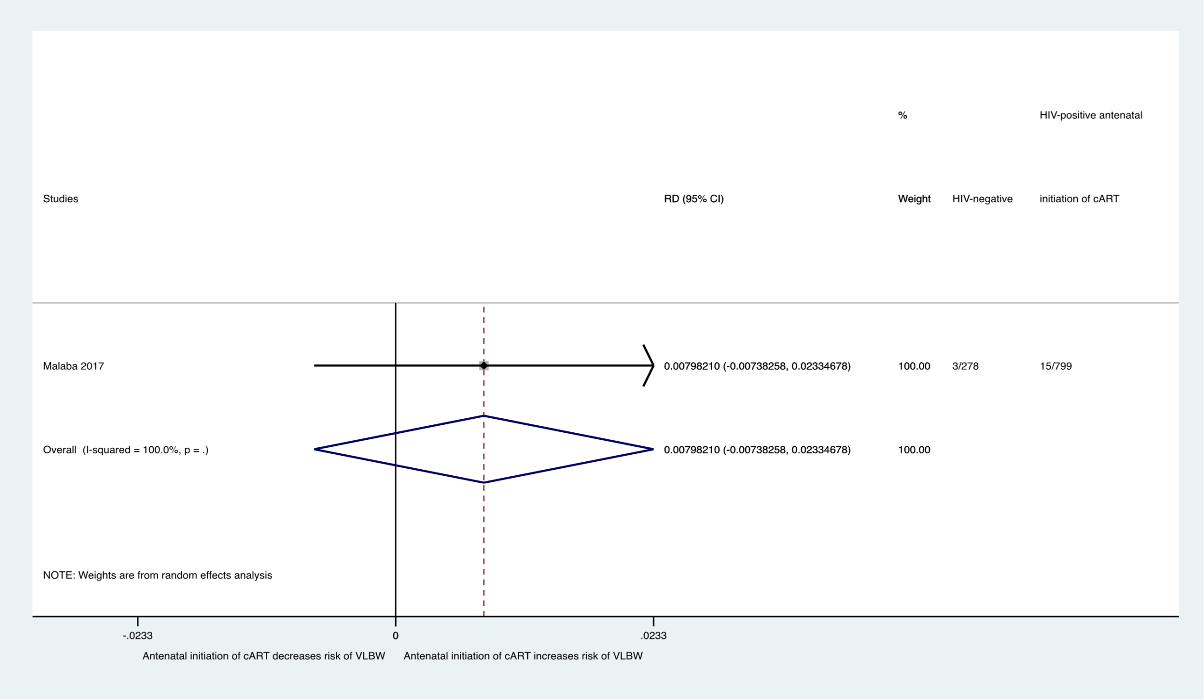

Figure 14: Very low birth weight (VLBW) in women living with HIV (WLHIV) receiving antenatal initiation of cART vs. HIV-negative women

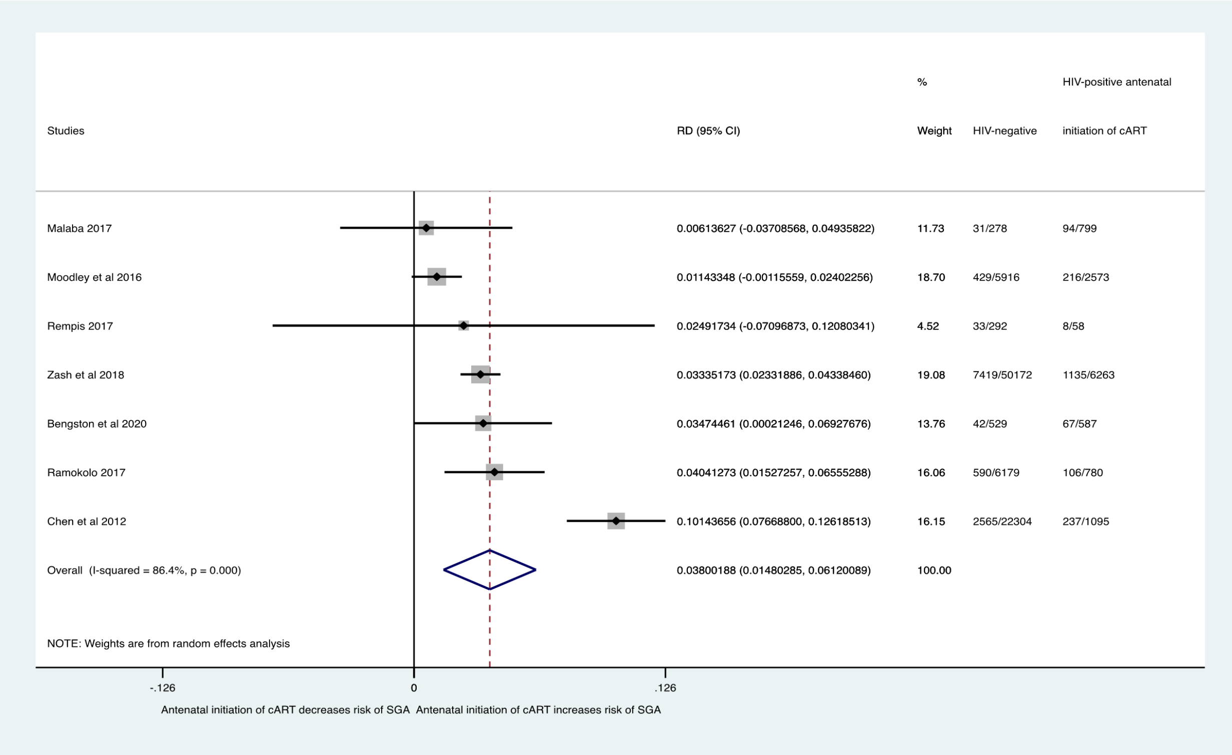

Figure 15: Small for gestational age (SGA) in women living with HIV (WLHIV) receiving antenatal initiation of cART vs. HIV-negative women

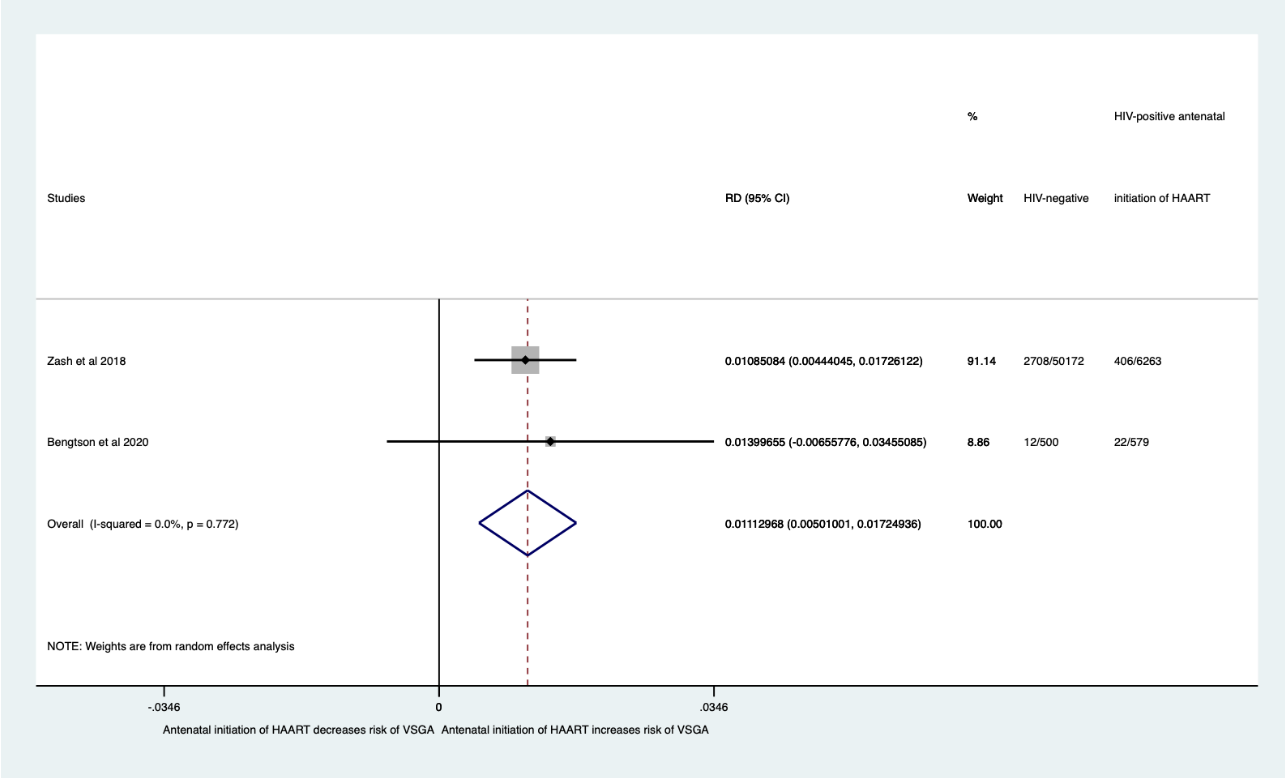

Figure 16: Very small for gestational age (VSGA) in women living with HIV (WLHIV) receiving antenatal initiation of cART vs. HIV-negative women

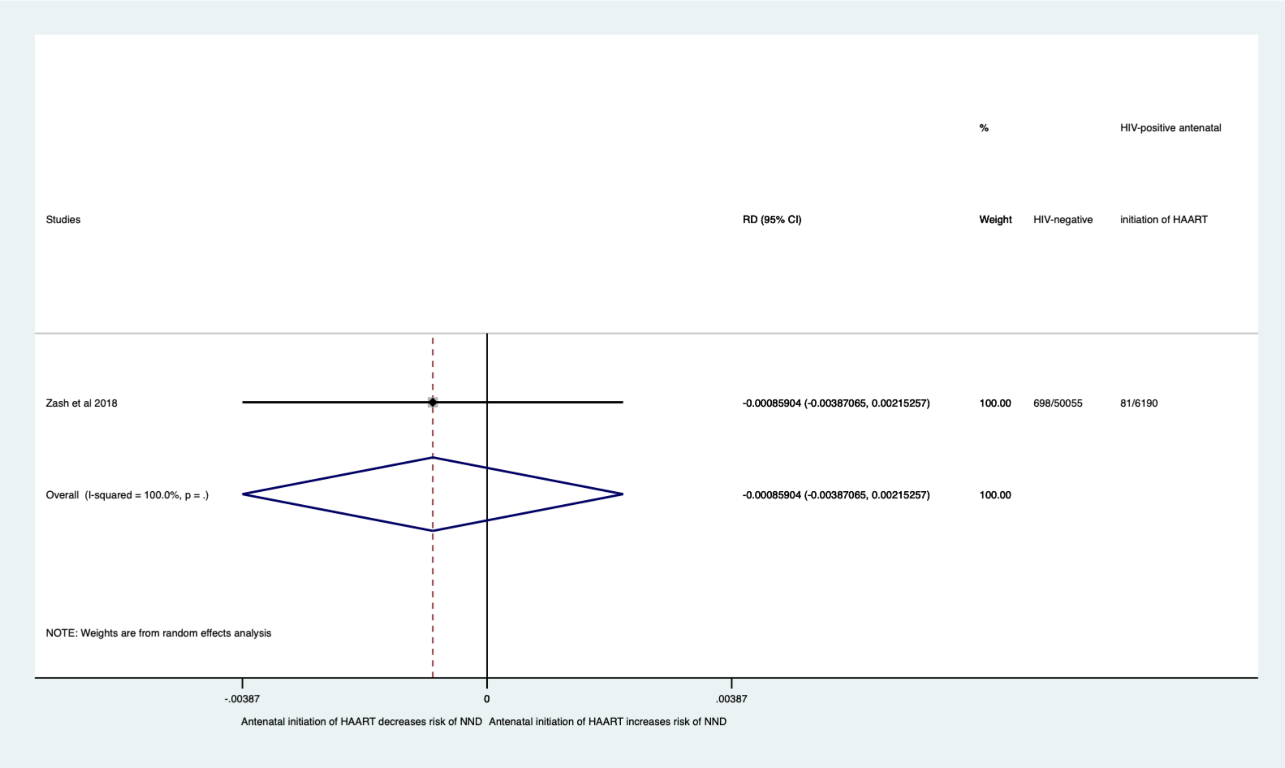

Figure 17: Neonatal death (NND) in women living with HIV (WLHIV) receiving antenatal initiation of cART vs. HIV-negative women

Women living with HIV receiving preconception cART vs HIV-negative women

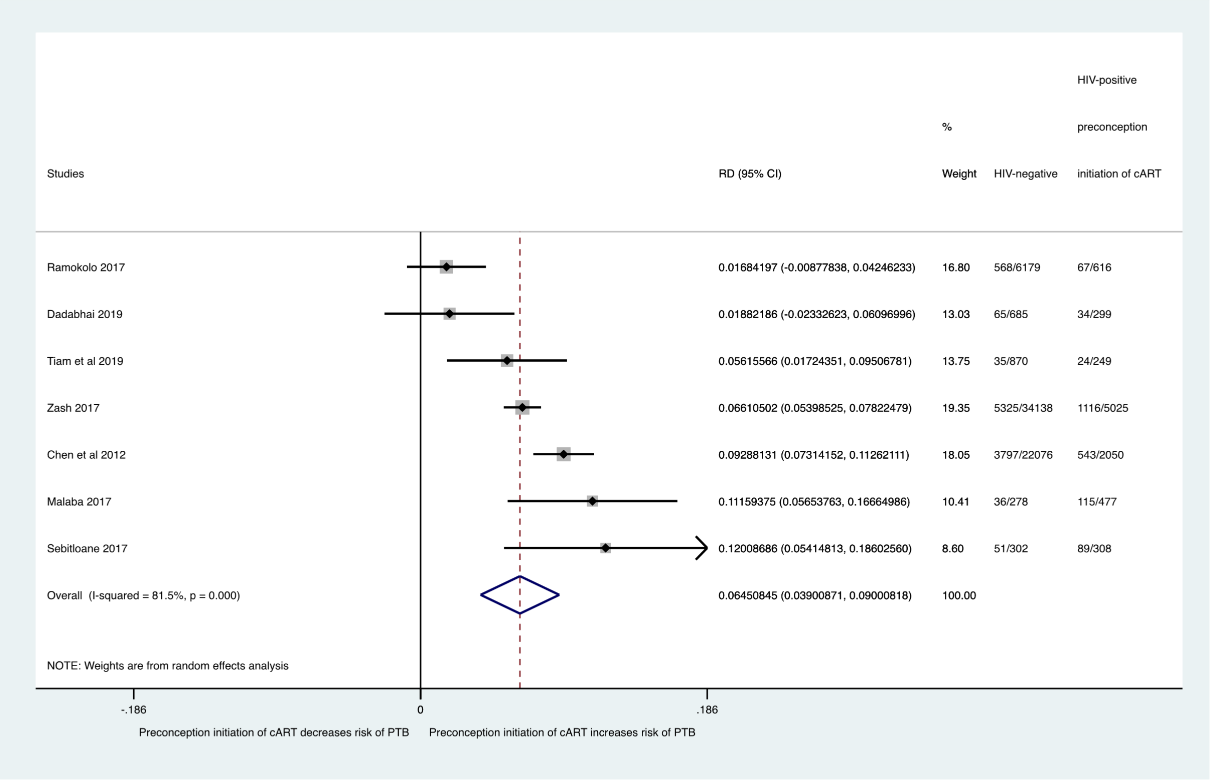

Figure 18: Preterm birth (PTB) in women living with HIV (WLHIV) receiving preconception initiation of cART vs. HIV-negative women

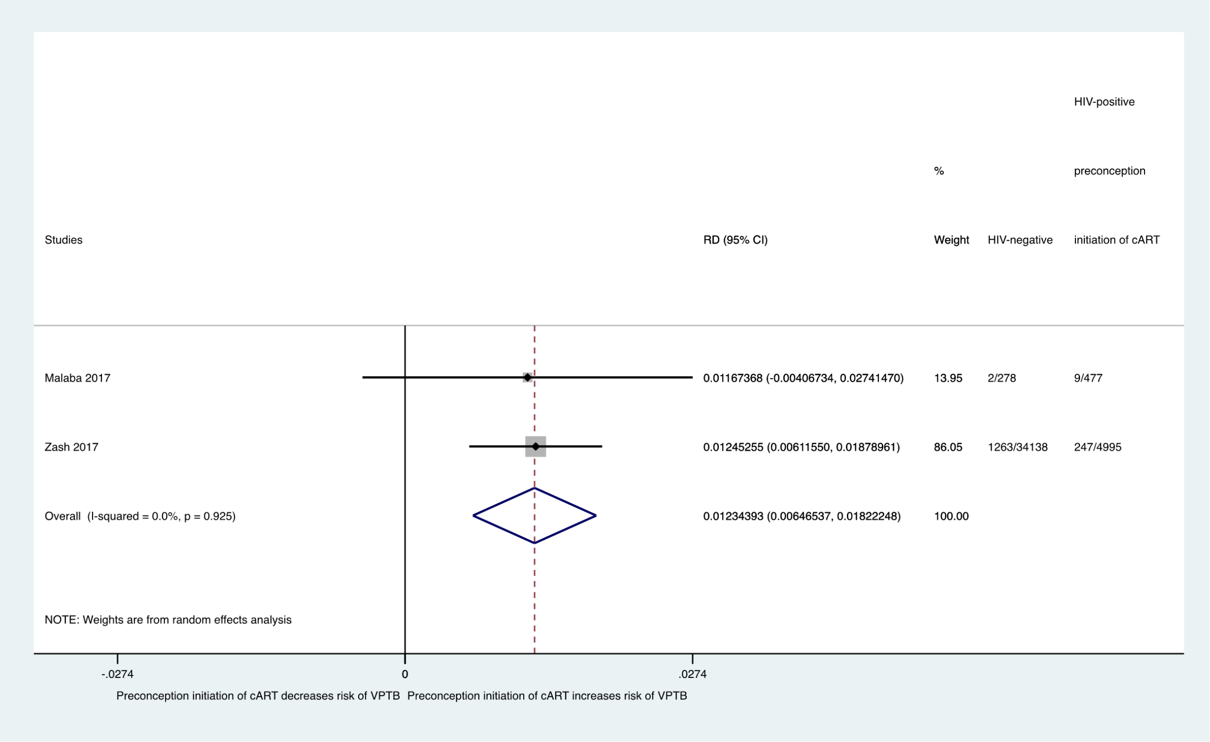

Figure 19: Very preterm birth (VPTB) in women living with HIV (WLHIV) receiving preconception initiation of cART vs. HIV-negative women

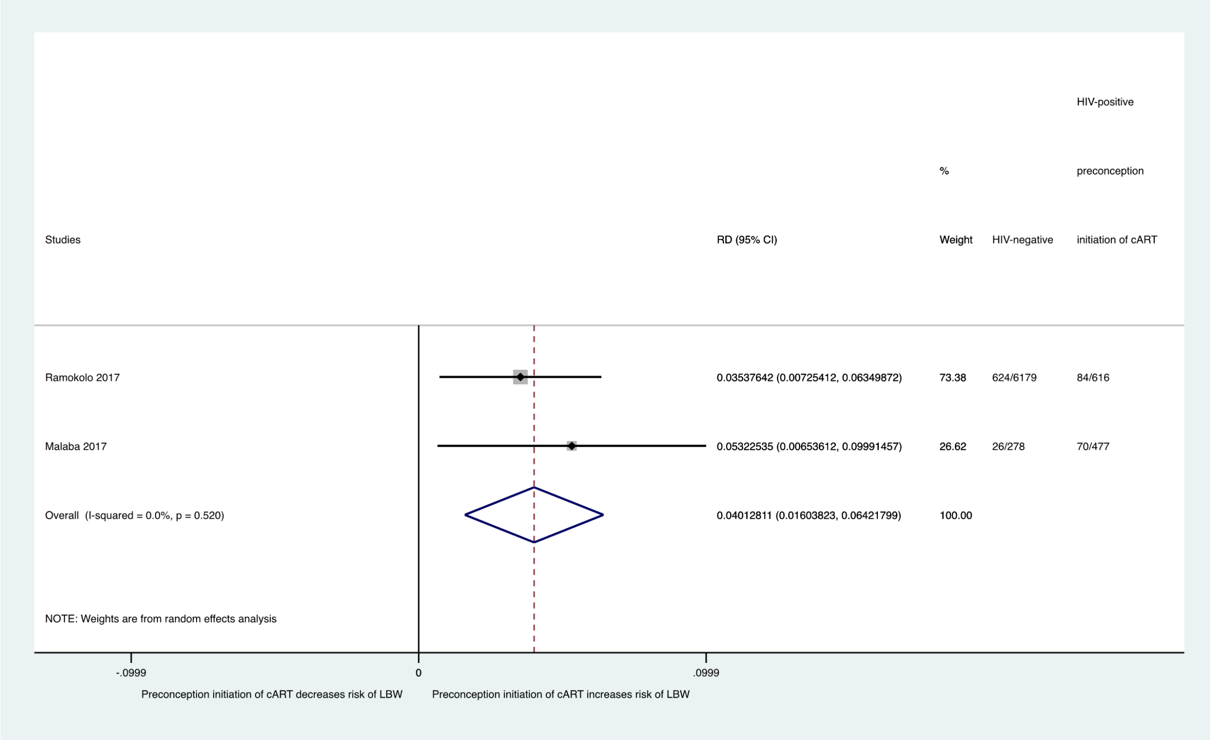

Figure 20: Low birth weight (LBW) in women living with HIV (WLHIV) receiving preconception initiation of cART vs. HIV-negative women

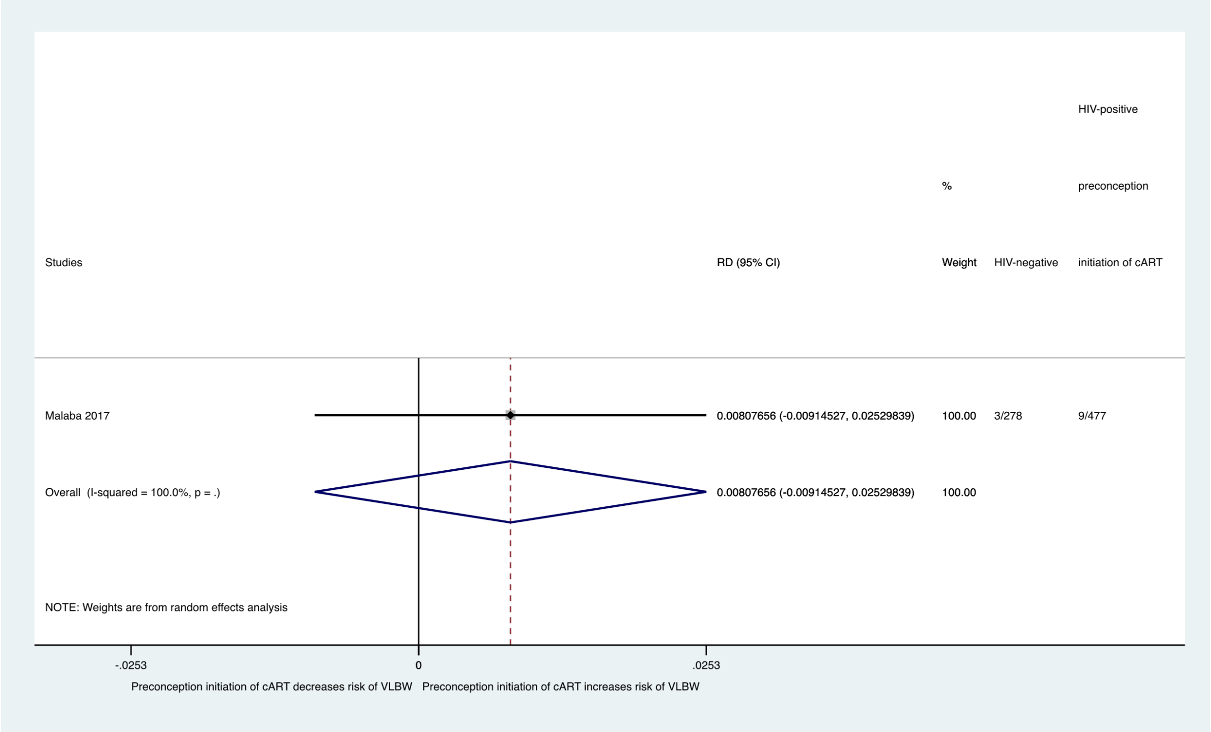

Figure 21: Very low birth weight (VLBW) in women living with HIV (WLHIV) receiving preconception initiation of cART vs. HIV-negative women

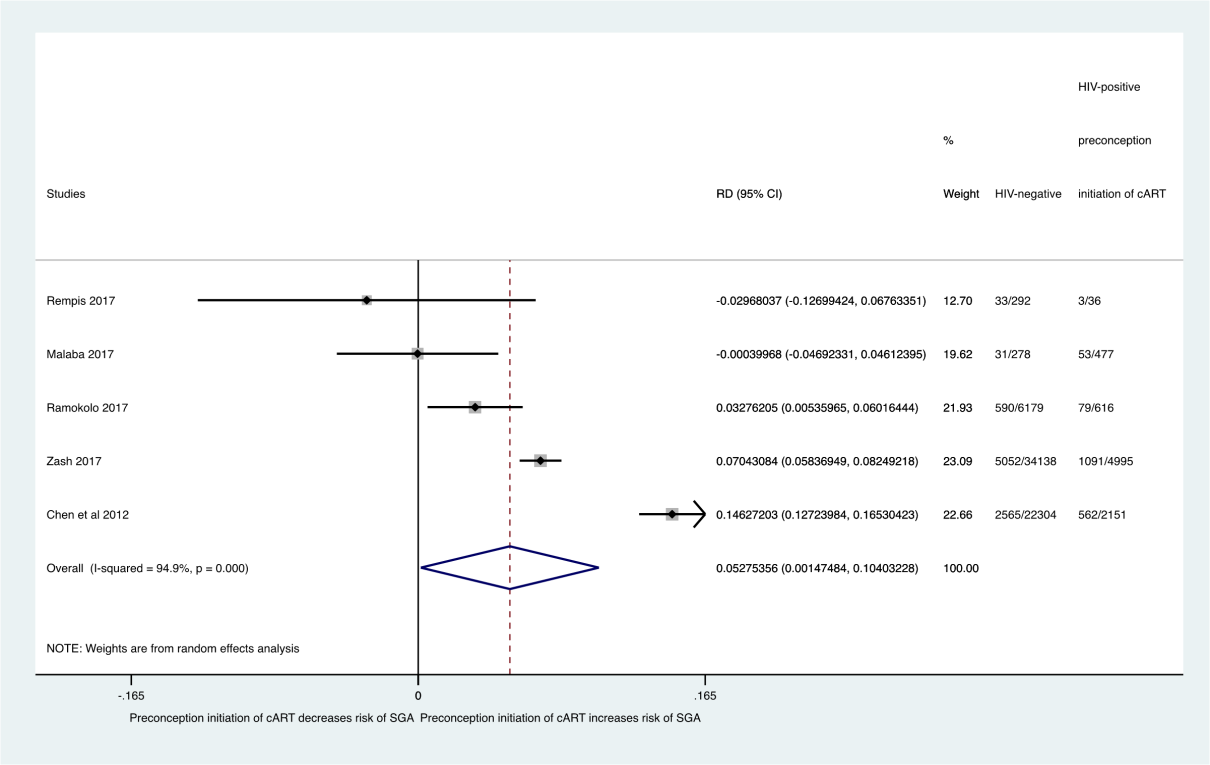

Figure 22: Small for gestational age (SGA) in women living with HIV (WLHIV) receiving preconception initiation of cART vs. HIV-negative women

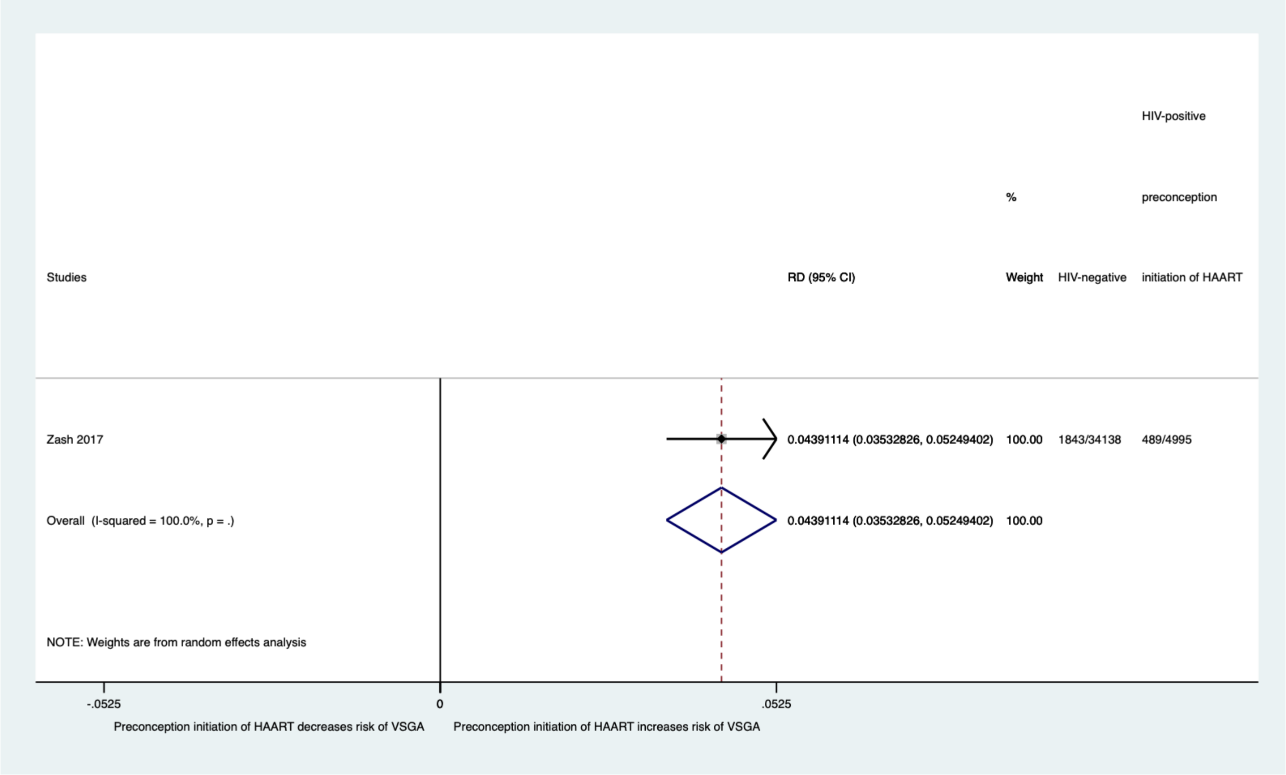

Figure 23: Very small for gestational age (VSGA) in women living with HIV (WLHIV) receiving preconception initiation of cART vs. HIV-negative women

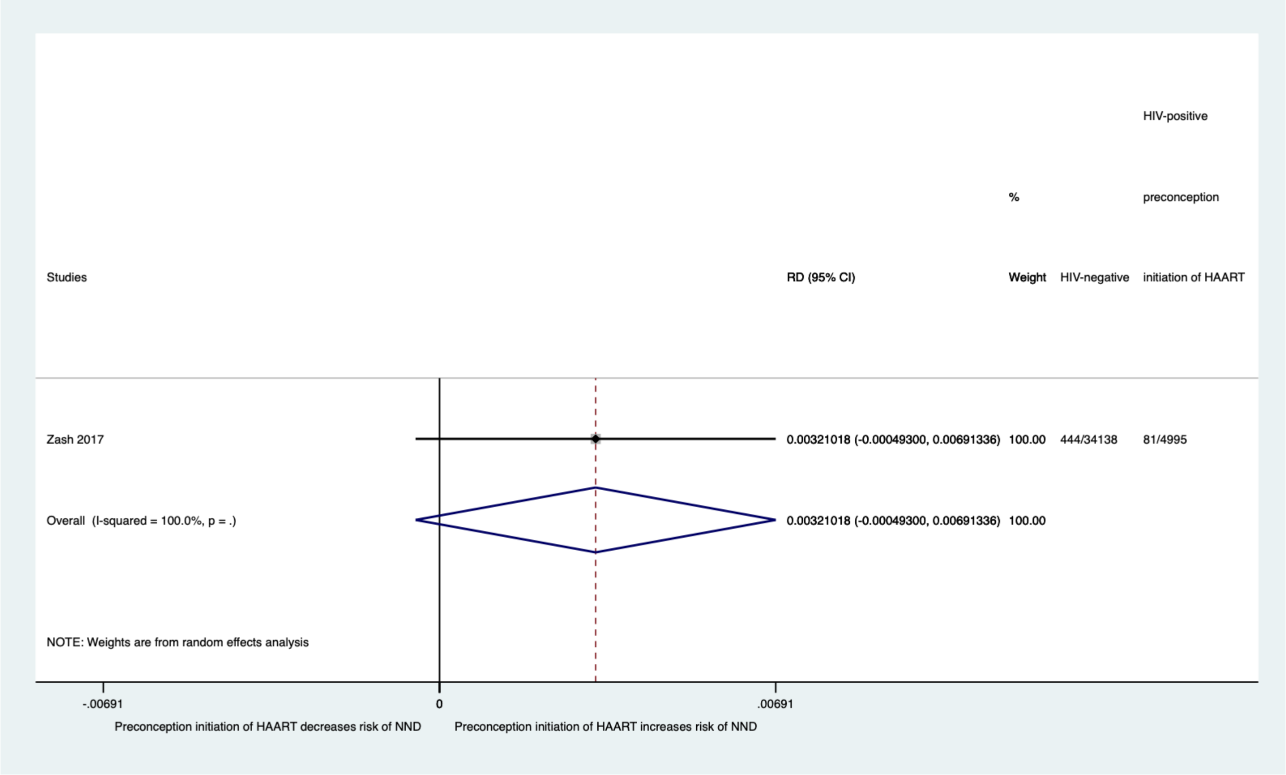

Figure 24: Neonatal death (NND) in women living with HIV (WLHIV) receiving preconception initiation of cART vs. HIV-negative women

Supplementary Figure 25

Subgroup analysis of women living with HIV receiving no treatment

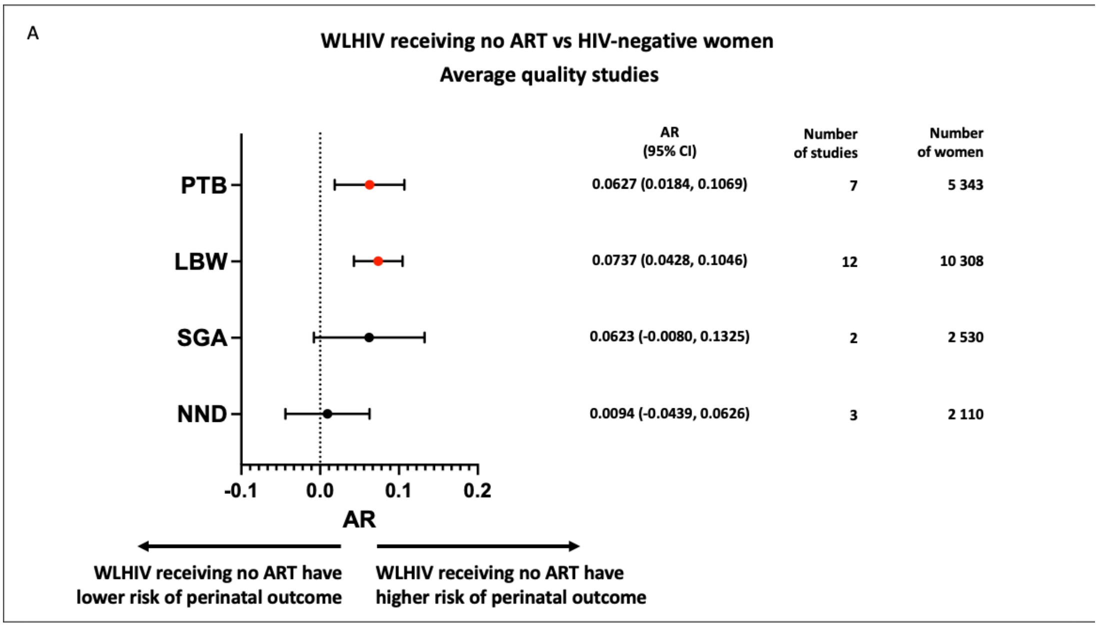

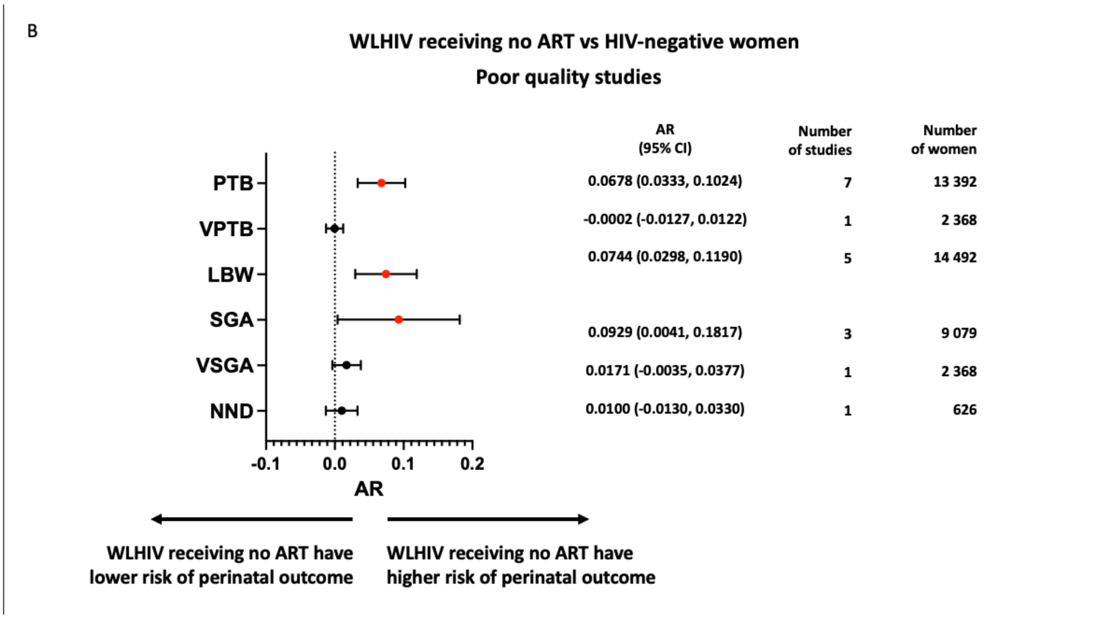

Results of random-effects meta-analyses to calculate attributable risk (i.e. risk difference) of perinatal outcomes associated with women living with HIV (WLHIV) receiving no ART, compared to HIV-negative women, for subgroups of average quality studies (A) and poor quality studies (B). Attributable risk and 95% confidence interval and numbers of studies and women included in the analysis of each perinatal outcome for each comparison are displayed.

Abbreviations: AR = attributable risk, ART = antiretroviral therapy, CI = confidence interval, low birth weight (LBW), neonatal death (NND), preterm birth (PTB), small for gestational age (SGA), very PTB (VPTB), very LBW (VLBW), very SGA (VSGA), WLHIV= women living with HIV.

Supplementary Figure 26

Subgroup analysis of women living with HIV receiving monotherapy

A

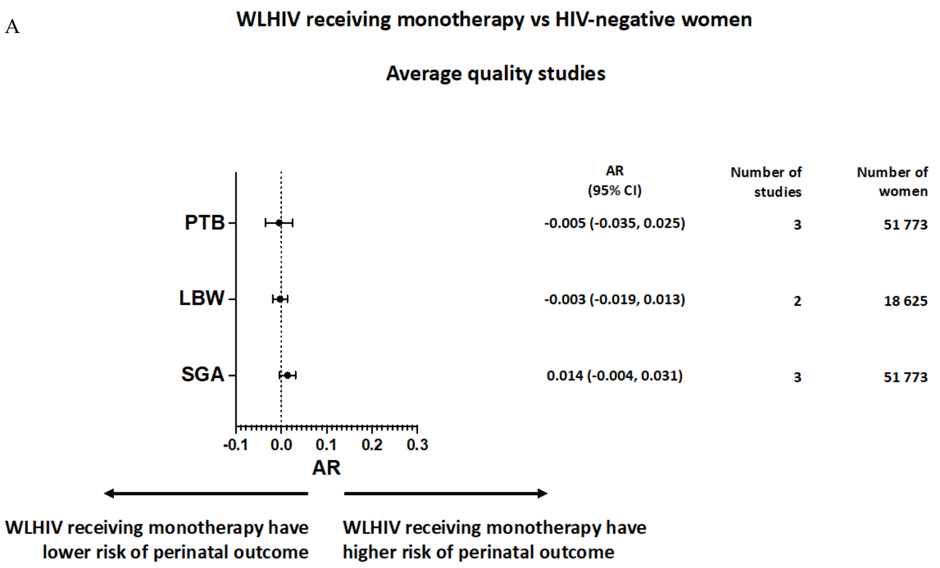

B

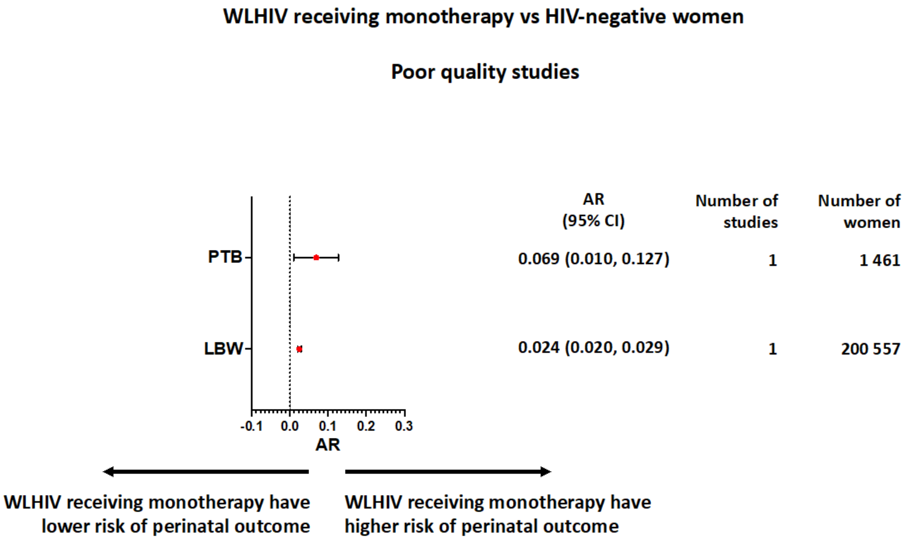

Results of random-effects meta-analyses to calculate attributable risk (i.e. risk difference) of perinatal outcomes associated with women living with HIV (WLHIV) receiving monotherapy, compared to HIV-negative women, for subgroups of average quality studies (A) and poor quality studies (B). Attributable risk and 95% confidence interval and numbers of studies and women included in the analysis of each perinatal outcome for each comparison are displayed.

Abbreviations: AR = attributable risk, CI = confidence interval, low birth weight (LBW), preterm birth (PTB), small for gestational age (SGA), WLHIV= women living with HIV.

Supplementary Figure 27

Subgroup analyses of women living with HIV receiving antenatal cART

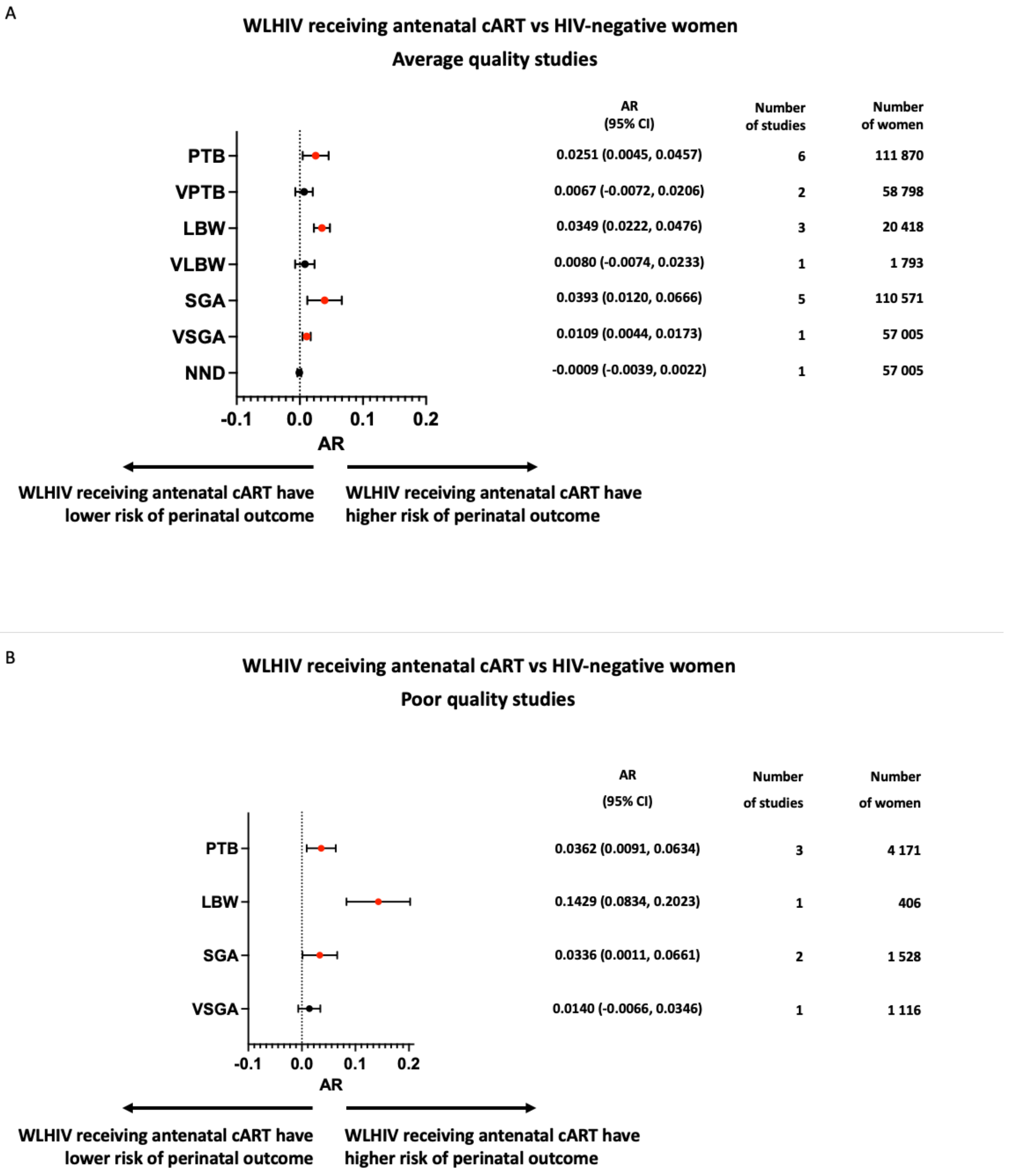

Results of random-effects meta-analyses to calculate attributable risk (i.e. risk difference) of perinatal outcomes associated with women living with HIV (WLHIV) receiving antenatal cART, compared to HIV-negative women, for subgroups of average quality studies (A) and poor quality studies (B). Attributable risk and 95% confidence interval and numbers of studies and women included in the analysis of each perinatal outcome for each comparison are displayed.

Abbreviations: AR = attributable risk, cART = combination antiretroviral therapy, CI = confidence interval, low birth weight (LBW), neonatal death (NND), preterm birth (PTB), small for gestational age (SGA), very PTB (VPTB), very LBW (VLBW), very SGA (VSGA), WLHIV= women living with HIV.

Supplementary Figure 28

Subgroup analysis of women living with HIV receiving preconception cART

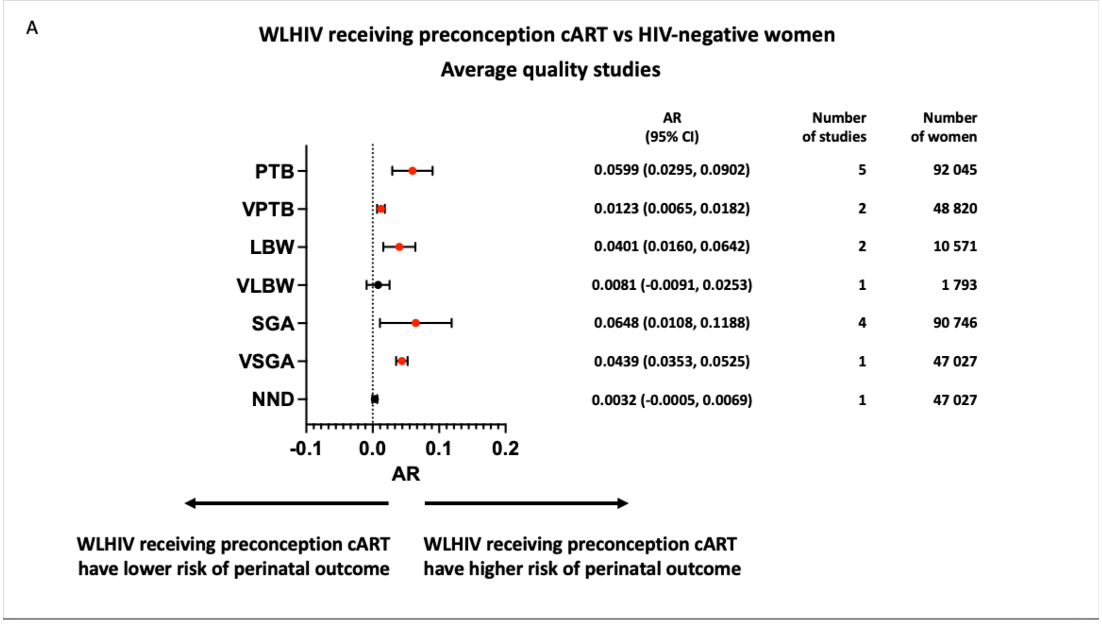

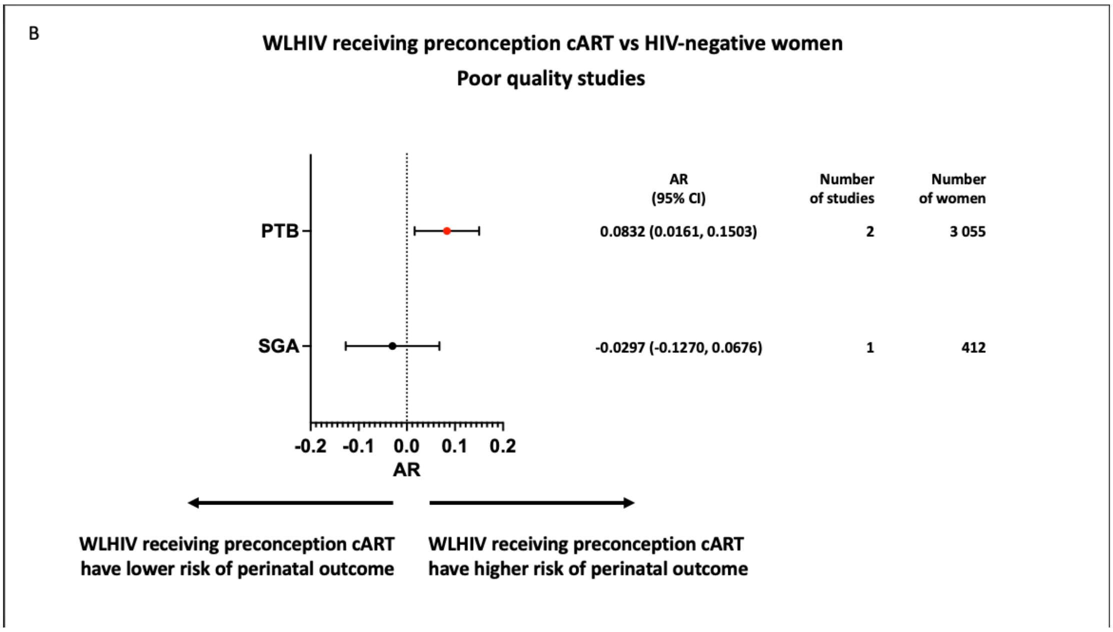

Results of random-effects meta-analyses to calculate attributable risk (i.e. risk difference) of perinatal outcomes associated with women living with HIV (WLHIV) receiving preconception cART, compared to HIV-negative women, for subgroups of average quality studies (A) and poor quality studies (B). Attributable risk and 95% confidence interval and numbers of studies and women included in the analysis of each perinatal outcome for each comparison are displayed.

Abbreviations: AR = attributable risk, cART = combination antiretroviral therapy, CI = confidence interval, low birth weight (LBW), neonatal death (NND), preterm birth (PTB), small for gestational age (SGA), very PTB (VPTB), very LBW (VLBW), very SGA (VSGA), WLHIV= women living with HIV.
